# Bridged [2.2.1] bicyclic phosphine oxide facilitates catalytic γ-umpolung addition–Wittig olefination†
†Electronic supplementary information (ESI) available. CCDC 1489646. For ESI and crystallographic data in CIF or other electronic format see DOI: 10.1039/c7sc04381c


**DOI:** 10.1039/c7sc04381c

**Published:** 2018-01-18

**Authors:** Kui Zhang, Lingchao Cai, Zhongyue Yang, K. N. Houk, Ohyun Kwon

**Affiliations:** a Department of Chemistry and Biochemistry , University of California , Los Angeles , California 90095-1569 , USA . Email: ohyun@chem.ucla.edu

## Abstract

A novel bridged [2.2.1] bicyclic phosphine oxide, devised to circumvent the waste generation and burdens of purification that are typical of reactions driven by the generation of phosphine oxides, has been prepared in three steps from commercially available cyclopent-3-ene-1-carboxylic acid. The performance of this novel phosphine oxide was superior to those of current best-in-class counterparts, as verified experimentally through kinetic analysis of its silane-mediated reduction. It has been applied successfully in halide-/base-free catalytic γ-umpolung addition–Wittig olefinations of allenoates and 2-amidobenzaldehydes to produce 1,2-dihydroquinolines with good efficiency. One of the 1,2-dihydroquinoline products was converted to known antitubercular furanoquinolines.

## Abstract 2

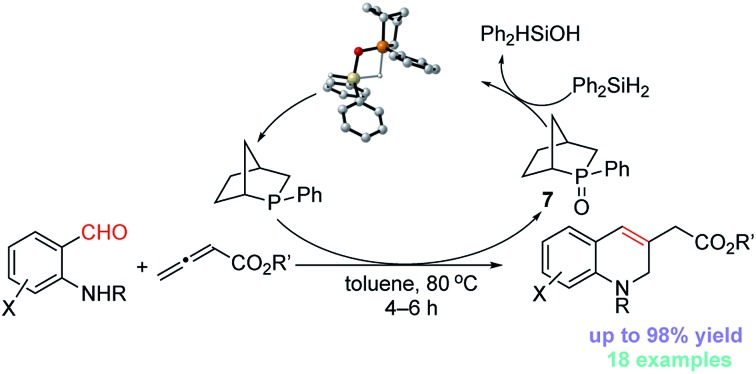
A bridged [2.2.1] bicyclic phosphine oxide has been created and applied successfully in halide-/base-free catalytic γ-umpolung addition–Wittig olefinations.

## Introduction

**Scheme 1:**
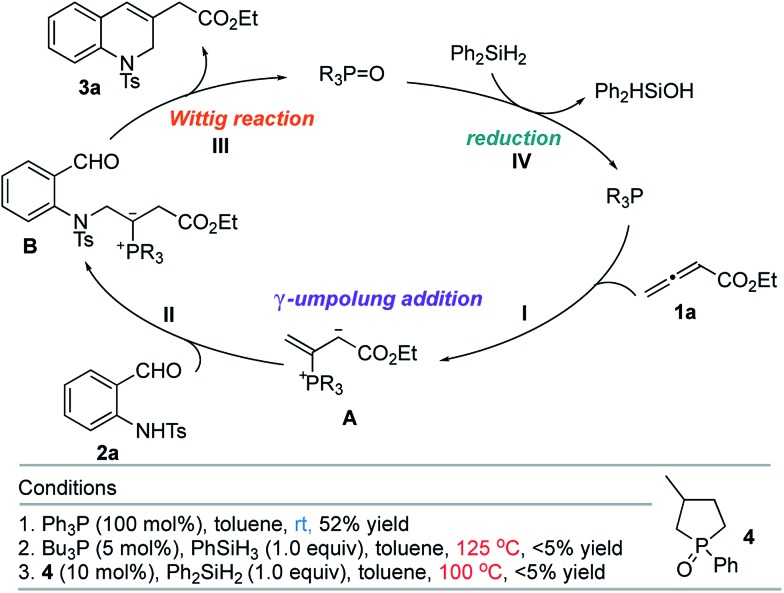
γ-Umpolung–Wittig reaction.

The classic Wittig reaction has been adopted widely for the construction of carbon–carbon double bonds since its discovery in 1953.^1^ The reaction is, however, far from ideal in terms of its environmental impact, requiring stoichiometric amounts of organic halide, base, and tertiary phosphine and producing stoichiometric amounts of base–halide salt and phosphine oxide wastes.^2^ Typically, the latter is not water-soluble, co-elutes with the olefin product during chromatographic separation, and (for a solid product) readily precipitates with the product, impeding its purification, particularly in large-scale processes (*e.g.*, those conducted on industrial scale).^3^ Traditionally, phosphorus ylides are prepared from corresponding phosphonium halides using stoichiometric amounts of base. Although less prevalent, ylides can also be prepared by simply mixing activated alkenes, acetylenes, and allenes with tertiary phosphines.^4^ In fact, halogen- and base-free Wittig olefinations between activated alkenes and aldehydes predate the venerable Morita–Baylis–Hillman reaction by several years.4*a* More recently, Werner, Lin, and Voituriez each reported halogen- and base-free catalytic Wittig olefinations.^5^ We also envisioned a halide- and base-free γ-umpolung–Wittig process.^6^ In our reaction, phosphine adds to the allenoate **1a** to form the zwitterionic intermediate **A** (Scheme 1). Deprotonation of the sulfonamide **2a** and γ-addition produces the ylide **B**, which, through a Wittig reaction, generates the 1,2-dihydroquinoline **3a** and produces the phosphine oxide (condition 1; steps **I** → **II** → **III**). To minimize the generation of the phosphine oxide byproduct, we turned our attention to the recently developed catalytic Wittig conditions.

In 2009, O'Brien and coworkers reported the first phosphine oxide-catalyzed Wittig reaction for the synthesis of alkenes, with the phosphine oxide byproduct reduced by a silane *in situ* back to the phosphine.7*a* This strategy has since been employed to promote Wittig, Appel, Staudinger, and Mitsunobu reactions, as well as other reactions that are thermodynamically driven by virtue of the formation of phosphine oxide.^7^–^9^ When we applied catalytic Bu_3_P5*b* or phospholane oxide **4** 7*a* under the reported reaction conditions, the desired dihydroquinoline was obtained in less than 5% yield (conditions 2 and 3; steps **I** → **II** → **III** → **IV**). Presumably, the allenoate **1a** oligomerized under the high temperatures. Consequently, we directed our study to the development of new phosphine oxides that can be reduced under milder conditions to realize the γ-umpolung–Wittig reaction in a catalytic mode.

## Results and discussion

**Fig. 1:**
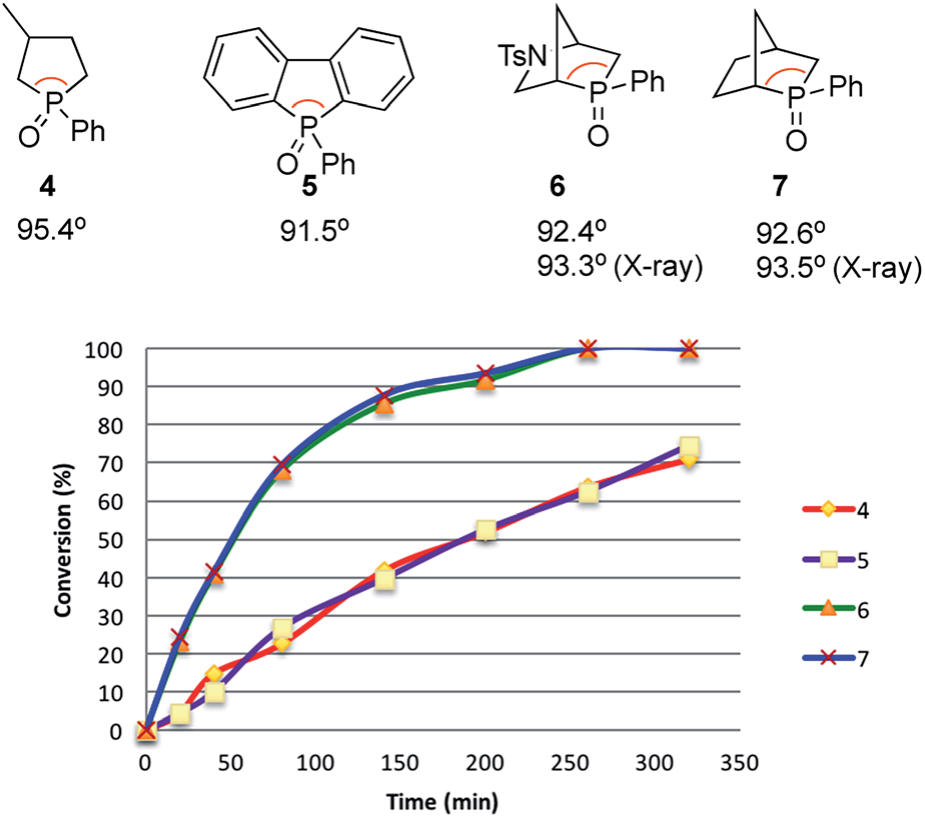
Diphenylsilane-mediated reductions of the phosphine oxides **4–7** and their computed C–P–C angles.

**Scheme 2:**
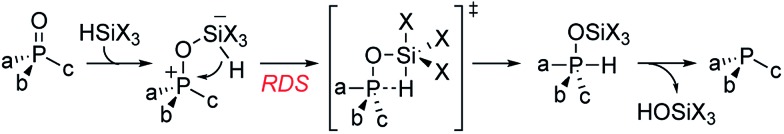
Silane-mediated reduction of a phosphine oxide.

Recently reported catalytic Wittig reactions have been mainly promoted by phospholane oxide **4** and dibenzophosphole oxide **5** (Fig. 1), which are reduced faster than larger-ring phosphacycloalkane oxides or acyclic counterparts.^7^,^8^ The currently accepted mechanism for silane-mediated reduction of phosphine oxides to phosphines is based on the studies of Horner and Mislow (Scheme 2).^10^ Krenske demonstrated computationally that the rate-determining step (RDS) of the reduction is an intramolecular hydride transfer from silicon to phosphorus after coordination of the phosphine oxide to the silane.^11^ The transition state (TS) for the hydride transfer features a four-membered P–O–Si–H ring, in which both the P and Si atoms are centers of two trigonal bipyramids bridged by O and H atoms. Consequently, we surmised that a phosphine oxide with an a–P–b angle close to 90° may undergo facile silane-mediated reduction, due to minimal structural deformation upon proceeding to the TS.^12^ Previously, we reported the invention of the [2.2.1] bicyclic phosphine oxide **6**, whose C–P–C angle in the X-ray structure was 93.3° (Fig. 1).^13^ Relative to the phospholane oxide **4** and the dibenzophosphole oxide **5**, compound **6** is reduced faster by a silane (*vide infra*). Accordingly, we designed the [2.2.1] bicyclic phosphine oxide **7**, which possesses a scaffold similar to that of **6**.^14^ Computational modeling predicted a C–P–C angle of 92.6°—much closer to 90° than the corresponding value (95.4°) of its non-bridged counterpart **4**.^15^,^16^ Despite its smaller endocyclic C–P–C angle (91.5°), the triarylphosphine oxide **5** is reduced at a rate similar to that of the dialkylarylphosphine oxide **4**, because more electron rich alkyl-substituted phosphine oxides are reduced faster than their aryl-substituted counterparts.5*b*

**Scheme 3:**
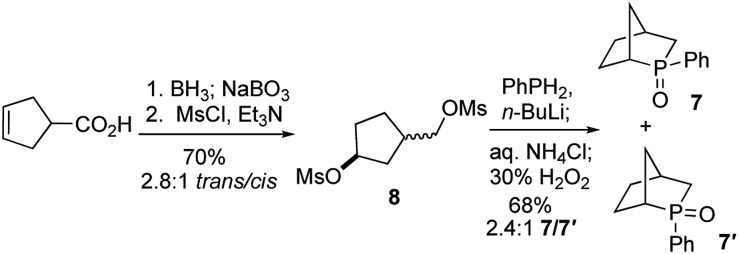
Synthesis of the phosphine oxide **7**.

To test our hypothesis, we synthesized the phosphine oxide **7**. As depicted in Scheme 3, reduction, hydroboration, and oxidation of commercially available cyclopent-3-ene-1-carboxylic acid, followed by bismesylation, produced the dimesylate **8** with 2.8 : 1 *trans*-to-*cis* selectivity, in 70% yield over two steps. A 2.4 : 1 mixture of the *exo*- and *endo-P*-phenylphosphine oxides **7** (*δ*_P_ = 55.8) and **7′** (*δ*_P_ = 57.4) was obtained in 68% isolated yield after bisalkylation with dilithium phenylphosphide and subsequent oxidative workup.^17^

With the phosphine oxide **7** in hand, we compared the efficiencies of the diphenylsilane-mediated reductions of the phosphine oxides **4–7**. The conversions of the phosphine oxides to the phosphines (diphenylsilane, 20 equiv.; CD_3_CN; 80 °C; 0.2 mol L^–1^) were monitored using ^31^P NMR spectroscopy (Fig. 1).^18^ At 20 min, the conversions of **4–7** were 4.5, 4.5, 23.2, and 24.3%, respectively. The reductions progressed steadily, with the phosphine oxides **6** and **7** reaching full conversion after 280 min; in contrast, the phosphine oxides **4** and **5** were not fully converted into their phosphines even after 6 h. The kinetic profiles of **6** and **7** were similar, as were those of **4** and **5**. The initial rates for **4** and **7** were 0.37 and 1.42 min^–1^, respectively.^15^ The ratio of these two initial rates (*r***_4_** : *r***_7_** = 1 : 4) corresponds to a 0.8 kcal mol^–1^ difference in activation free energies, based on the Boltzmann distribution. Computational studies were conducted to rationalize the reactivity difference between **4** and **7**. The TSs for the reductions of **4** and **7** by diphenylsilane were located using the B3LYP method in Gaussian 09, with single point energy computed under B3LYP method with Grimme D3 correction. The calculated activation free energies for substrates **4** and **7** were 30.1 and 29.3 kcal mol^–1^, respectively—a difference of 0.8 kcal mol^–1^, consistent with the experimental observation.^15^

Having confirmed the ready reducibility of the phosphine oxide **7** experimentally, our attention turned to applying this bridged bicyclic phosphine oxide to halide-/base-free catalytic γ-umpolung–Wittig olefination. Initially, we employed ethyl allenoate (**1a**) and *o*-(*p*-toluenesulfonamido)benzaldehyde (**2a**) as model reactants to identify suitable reaction conditions for the catalytic γ-umpolung–Wittig olefination (Table 1). We used diphenylsilane as the reducing reagent in the reactions catalyzed by the various phosphines or phosphine oxides at a reaction temperature of 80 °C (entries 1–6). Little to no catalytic turnover occurred when applying triphenylphosphine or tributylphosphine, presumably due to the lack of ring strain.^19^ The phosphine oxides **4–7** produced the dihydroquinoline product in yields of 62, 23, 76, and 81%, respectively. Notably, the reaction employing **7** took only 4 h to reach completion, while that of **4** was sluggish (24 h). Much starting material remained after 48 h for the reaction performed using the phosphine oxide **5**. Diphenylsilane was more efficient (81%) as a reducing agent than were phenylsilane and triphenylsilane (74 and 0%, respectively) under otherwise identical reaction conditions (entries 7 and 8). A survey of reaction temperatures revealed that the yield did not improve when run at either higher (oligomerization of allenoate) or lower temperature (entries 9 and 10). The reaction concentration also had a discernible effect on the reaction efficiency, with 0.03 M being optimal (entries 11 and 12). Finally, with decreased loading of the phosphine oxide **7** to 20 and 15 mol%, the reactions were prolonged to 12 and 29 h, respectively, with diminished product yields (entries 13 and 14). To our delight, the reaction time shortened to 4 h and the yield improved to 88% when 15 mol% of the phosphine oxide **7** was pre-reduced by diphenylsilane prior to addition of the reactants (entry 15). In addition, here we added the allenoate **1a** using a syringe pump to minimize its oligomerization. The yield declined to 75% when 10 mol% of the phosphine oxide **7** was employed in the same manner (entry 16). Thus, the standard reaction conditions were established: **1a** (0.6 mmol, 3 equiv.), **2a** (0.2 mmol), Ph_2_SiH_2_ (0.4 mmol, 2 equiv.) as the reducing agent, and the phosphine oxide **7** (0.03 mmol, 0.15 equiv.) as the catalyst at 80 °C.

**Table 1:** Optimization of catalytic γ-umpolung/Wittig reaction^*a*^

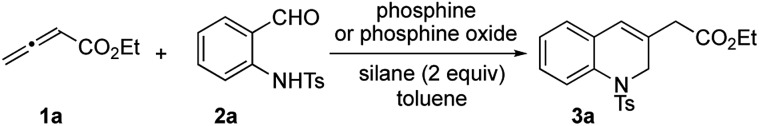						
Entry	Phosphine (oxide)	*C* (mol L^–1^)	Silane	Temp. (°C)	Time (h)	Yield^*b*^ (%)
1	PBu_3_ (30%)	0.05	Ph_2_SiH_2_	80	24	—
2	PPh_3_ (30%)	0.05	Ph_2_SiH_2_	80	24	39
3	**4** (30%)	0.05	Ph_2_SiH_2_	80	24	62
4^*c*^	**5** (30%)	0.05	Ph_2_SiH_2_	80	48	23
5	**6** (30%)	0.05	Ph_2_SiH_2_	80	12	76
6	**7** (30%)	0.05	Ph_2_SiH_2_	80	4	81
7	**7** (30%)	0.05	PhSiH_3_	80	4	74
8	**7** (30%)	0.05	Ph_3_SiH	80	24	—
9	**7** (30%)	0.05	Ph_2_SiH_2_	100	3	42
10	**7** (30%)	0.05	Ph_2_SiH_2_	70	24	38
11	**7** (30%)	0.1	Ph_2_SiH_2_	80	2	47
12	**7** (30%)	0.03	Ph_2_SiH_2_	80	6	83
13	**7** (20%)	0.03	Ph_2_SiH_2_	80	12	82
14	**7** (15%)	0.03	Ph_2_SiH_2_	80	29	75
15^*d*^ ^,^^*e*^	**7** (15%)	0.03	Ph_2_SiH_2_	80	4	88
16^*d*^ ^,^^*e*^	**7** (10%)	0.03	Ph_2_SiH_2_	80	13	75

^*a*^Performed in a vial containing **1a** (0.6 mmol), **2a** (0.2 mmol), a silane (0.4 mmol), and a phosphine or phosphine oxide in toluene.

^*b*^Isolated yield.

^*c*^Much starting material (**2a**) remained after 2 days.

^*d*^ **7** was reduced by Ph_2_SiH_2_ in toluene before adding **1a** and **2a**.

^*e*^ **1a** was added using a syringe pump to prohibit oligomerization.

To track the evolution of the phosphine oxide catalyst **7** during the catalytic cycle, the reaction mixture was examined spectroscopically over the course of the entire process under the optimized conditions. ^31^P NMR spectroscopy revealed that the resting state of catalyst was the phosphine oxide.^15^ This observation is in agreement with our premise that the RDS of the whole catalytic cycle is the reduction of the phosphine oxide to the phosphine, and that the overall reaction efficiency reflects that of the phosphine oxide's reducibility.

**Scheme 4:**
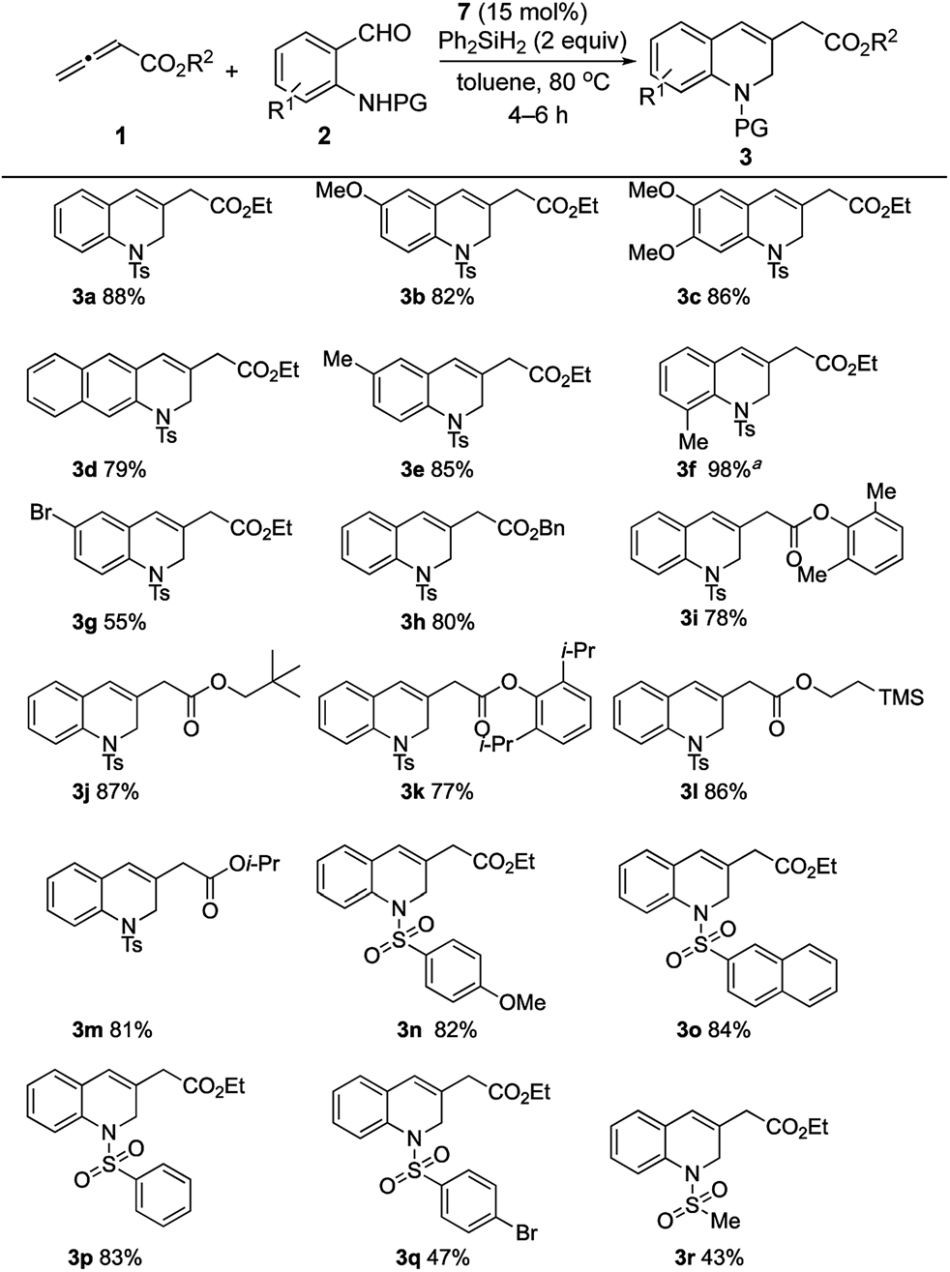
Synthesis of 1,2-dihydroquinolines. General conditions: **7** (30 μmol) and Ph_2_SiH_2_ (0.4 mmol) were stirred in toluene (0.5 mL) at 80 °C for 1 h, followed by addition of neat **2** (0.2 mmol), then **1** (0.6 mmol, 0.11 M in toluene), using a syringe pump, over 2 h. The isolated yields are shown. ^*a*^Reaction was complete within 20 h.

The generality of this catalytic γ-umpolung–Wittig olefination was probed using various allenoates and *o*-sulfonamidobenzaldehydes (Scheme 4).^20^ Electron-donating substituents on the benzaldehyde ring led to the 1,2-dihydroquinoline derivatives **3a–3f** all being generated in high yields when coupling with the allenoate **1a**. When using 2-(4-toluenesulfonamido)-3-methylbenzaldehyde, a prolonged reaction time was required, presumably because of steric effects; the reaction was, however, clean, furnishing the desired product **3f** in quantitative yield. When 5-bromo-2-(4-toluenesulfonamido)benzaldehyde, bearing an electron-withdrawing bromine substituent, was used, the yield was 55% (**3g**).^21^ The compatibility of an aryl bromide with the current reaction is especially notable in light of its suitability for further functionalization of the dihydroquinoline products.

We then studied the allenoate substrate scope by treating **2a** with a series of allenoates, which all afforded their corresponding 1,2-dihydroquinolines (**3h–3m**) in high yields. γ-Phenylallenoate and α-methylallenoate did not produce the desired products, presumably due to increased steric hindrance.^21^ Subsequently, various protecting groups of the amide were probed. *N*-(2-Formylphenyl)-4-methoxybenzenesulfonamide, *N*-(2-formylphenyl)naphthalene-2-sulfonamide, and *N*-(2-formylphenyl)benzenesulfonamide produced their desired products **3n**, **3o**, and **3p**, respectively, in good yields (82–84%). A sulfonamide with an electron-withdrawing 4-bromo group gave its expected product **3q** in moderate yield, presumably because of the diminished nucleophilicities of its sulfonamide unit during the γ-umpolung addition.^22^ The reaction of *N*-(2-formylphenyl)methanesulfonamide and ethyl allenoate gave **3r** in 43% yield; we suspect that the acidic methyl protons of the methanesulfonamide might have had a deleterious effect on the overall reaction efficiency.

**Scheme 5:**
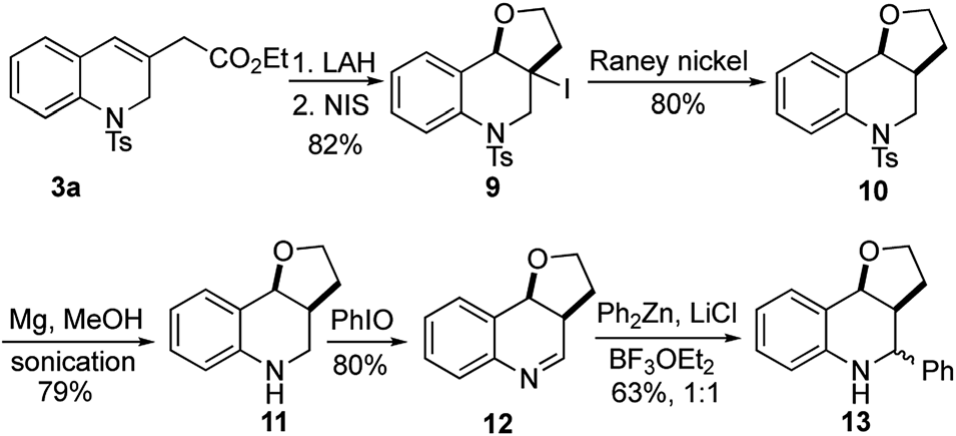
Synthesis of antitubercular furanoquinolines **13**.

The utility of this γ-umpolung/Wittig reaction was further explored in the synthesis of the furanoquinolines **13**, which have shown antitubercular activity (Scheme 5).^23^ For this synthesis, we reduced the ester **3a** with lithium aluminum hydride (LAH), followed by iodoetherification with *N*-iodosuccinimide (NIS), to obtain the furan **9** in 82% yield over two steps. The core structure **10** was obtained after deiodination with RANEY® Ni. Compound **10** was treated with Mg in MeOH under sonication to reductively cleave the tosyl group. Subsequent iodosobenzene-mediated oxidation yielded the imine **12** in 80% yield. The synthetic target **13** was obtained in 63% yield as a 1 : 1 mixture of diastereoisomers after nucleophilic addition of diphenylzinc.

## Conclusions

We have developed a new bridged [2.2.1] bicyclic phosphine oxide **7** that displays exceptional reduction efficiency in the presence of diphenylsilane. Although the catalytic Wittig reaction has been developing rapidly for several years, there remains room for the development of phosphine oxide structures to improve the efficiency of their silane-mediated reductions. The utility of the bridged bicyclic phosphine oxide **7** was exemplified in catalytic halide- and base-free γ-umpolung–Wittig reactions of allenoates and *o*-sulfonamidebenzaldehydes, providing 1,2-dihydroquinolines. One of the γ-umpolung–Wittig reaction products, **3a**, was elaborated in the synthesis of the antitubercular furanoquinolines **13**. Further refinement of the phosphine oxide scaffold and studies of the applications of these phosphine oxides in other catalytic processes are underway in our laboratories.

## Conflicts of interest

There are no conflicts to declare.

## Supplementary Material

Supplementary information

Crystal structure data
