# Supplementary material for: Bridged [2.2.1] bicyclic phosphine oxide facilitates catalytic γ-umpolung addition–Wittig olefination
Source: Chem Sci. 2018 Jan 18;9(7):1867–72. doi: 10.1039/c7sc04381c (PMC5909331; doi:10.1039/c7sc04381c)

Electronic Supplementary Material (ESI) for Chemical Science.
This journal is © The Royal Society of Chemistry 2018

Supporting Information

Bridged [2.2.1] Bicyclic Phosphine Oxide Facilitates Catalytic γ-Umpolung Addition–Wittig Olefination

Kui Zhang,‡ Lingchao Cai,‡ Zhongyue Yang, K. N. Houk, and Ohyun Kwon*

Department of Chemistry and Biochemistry, University of California, Los Angeles, California 90095-1569, United States

Table of Contents

1. General Information..2
2. Computational Study..2
    2.1 Computational Methods..2
    2.2 Phosphine Oxide Structures and C–P–C Angles..3
    2.3 Geometries and Energies of the Phosphine Oxides **4**, **7** and Diphenylsilane..4
    2.4 Transition State Geometries and Relative Free Energies for Reduction of the Phosphine Oxide **4**..7
    2.5 Transition State Geometries and Relative Free Energies for Reduction of the Phosphine Oxide **7**..13
    2.6 Benchmark of Reactants and Transition States for the Phosphine Oxides **4**, **7**, and **S2** Using High-Level DFT Methods Combined With 6-311+G(d,p) Basis Set........20
    2.7 Distortion–Interaction Analysis of the Activation Barriers for Diphenylsilane-Mediated Reductions of Substrates **4**, **7**, and **S2**..21
    2.8 Geometries and Energies of Phosphine Oxides **7′**, **S1** and **S2**..22
    2.9 Transition State Geometries and Energies for the Phosphine Oxides **4**, **7**, **7′**, **S1**, and **S2**..24
    2.10 Geometries and Energies of Phosphine Oxides **5**, **6**, **7a**, Ph_3PO, and Bu_3PO..28
3. Synthesis and Reduction of the Phosphine Oxides **7** and **7′**..31
4. NMR Spectroscopic Studies of Silane-Mediated Reduction of the Phosphine Oxides **4–7′**, **S1** and **S2**..**Error! Bookmark not defined.**
5. Kinetics Profiles, Trend Lines, and Fitting Equations for Substrates **4–7′**, **S2**, and **7a** 40
6. NMR Spectroscopic Studies of the γ-Umpolung–Wittig Reaction..41
7. Preparation of Substrates..42
    7.1 Allenoates..42
    7.2 General Procedure for Preparation of Substrates **2**..43
8. γ-Umpolung/Wittig Reactions..43
9. Synthesis of the Furanoquinoline **13**..48
10. References..51
11. ^1H, ^{13}C, and ^{31}P NMR Spectra..52

1. General Information

All reactions were performed in dry solvents under an Ar atmosphere and anhydrous conditions. Toluene was freshly distilled over CaH_2 prior to use. All other reagents were used as received from commercial sources. Reactions were monitored through thin layer chromatography (TLC) on 0.25-mm SiliCycle silica gel plates and visualized under UV light. Flash column chromatography (FCC) was performed using SiliCycle Silica-P Flash silica gel (60-Å pore size, 40–63 μm). IR spectra were recorded using a Jasco FT-IR 4100 spectrometer. NMR spectra were recorded using Bruker Avance-300 and Bruker Avance-500 instruments, calibrated to $CD(H)Cl_3$ as the internal reference (7.26 and 77.0 ppm for ^1H and ^{13}C NMR spectra, respectively). ^1H NMR spectral data are reported in terms of chemical shift (δ, ppm), multiplicity, coupling constant (Hz), and integration. ^{13}C NMR spectral data are reported in terms of chemical shift (δ, ppm) and multiplicity, with the coupling constant (Hz) in the case of J_{CP} coupling. The following abbreviations indicate the multiplicities: s, singlet; d, doublet; t, triplet; q, quartet; m, multiplet. High-resolution mass spectra were recorded using a Waters LCT Premier XE time-of-flight instrument controlled by MassLynx 4.1 software. Samples were infused through direct loop injection from a Waters Acquity UPLC into the multi-mode ionization source. The lock mass standard for accurate mass determination was leucine enkephalin (Sigma L9133). X-ray crystallographic data were collected using a Bruker SMART CCD-based diffractometer equipped with a low-temperature apparatus operated at 100 K.

2. Computational Study

2.1 Computational Methods

All density functional theory (DFT) computations were performed using Gaussian09. The conformational search for the transition states (TSs) for the reduction of the phosphine oxides **4** and **7** was conducted using the B3LYP/6-31G(d) level of theory, combined with single-point energy calculations at the B3LYP-D3/6-311+G(d,p) level in the gas phase. More detail is provided in Sections 2.4 and 2.5. Based on the most optimal conformation, the free energy profiles for substrates **4, 5, 6, 7**, **7′**, **7a**, **S1**, **S2**, Ph_3PO, and Bu_3PO and the TS free energy profiles for their reduction were studied (see each section for details). Geometry optimization was performed at the B3LYP/6-31G(d) level of theory in implicit acetonitrile using the SMD model [abbreviated as SMD(CH_3CN)-B3LYP/6-31G(d) in the following discussion]. Normal vibrational mode analysis at the same level confirmed that optimized structures were minima or TSs. The frequencies were corrected using quasi-harmonic approximation, which elevated the lower frequencies (<100 cm^{-1}) to 100 cm^{-1}, to overcome the problem of anharmonicity. Single-point energy calculations were conducted using SMD(CH_3CN)-B3LYP-D3/6-311+G(d,p) based on the optimized structures. The selection of the method is further elaborated in the benchmark presented in Section 2.6.

2.2 Phosphine Oxide Structures and C–P–C Angles

Phosphine oxide	X-ray structure	Computational structure	TS geometry for reduction
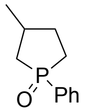 **4**		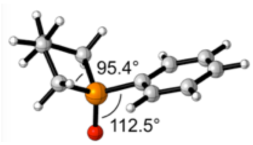	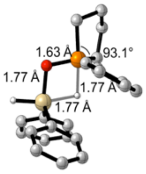 $\Delta G^{\ddagger}$ = 30.1 kcal/mol
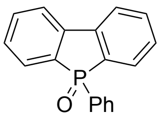 **5**		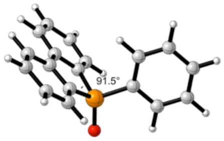	
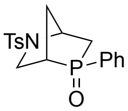 **6**	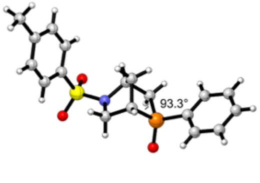	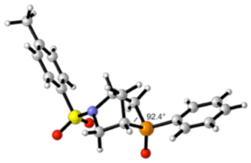	
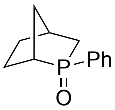 **7**	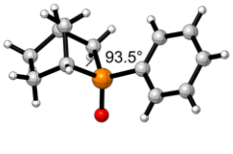	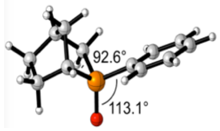	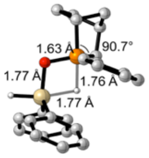 $\Delta G^{\ddagger}$ = 29.3 kcal/mol
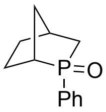 **7′**		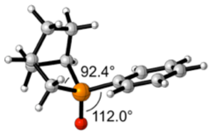	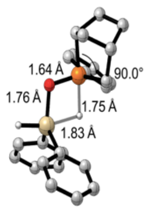 $\Delta G^{\ddagger}$ = 29.5 kcal/mol
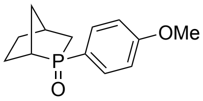 **7a**		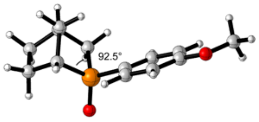	

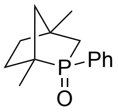 **S1**		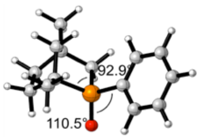	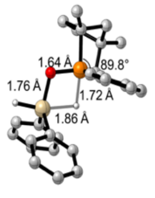 $\Delta G^{\ddagger}$ = 30.4 kcal/mol
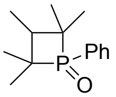 **S2**		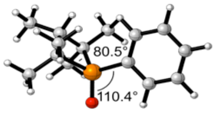	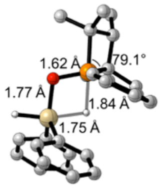 $\Delta G^{\ddagger}$ = 30.0 kcal/mol
Ph_3PO		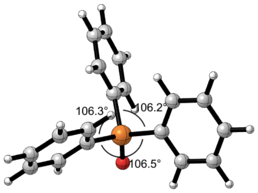	
Bu_3PO		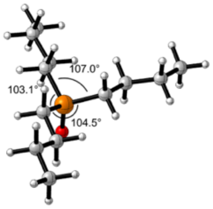	

2.3 Geometries and Energies of the Phosphine Oxides 4, 7 and Diphenylsilane

The geometry optimization was performed at the B3LYP/6-31G(d) level of theory in implicit acetonitrile using the SMD(CH_3CN)-B3LYP/6-31G(d) method. Normal vibrational mode analysis at the same level confirmed that optimized structures were minima or TSs. The frequencies were corrected using quasi-harmonic approximation, as explained in Section 2.1. Single-point energy calculations were conducted using SMD(CH_3CN)-B3LYP-D3/6-311+G(d,p) based on the optimized structures. The selection of the method is further elaborated in the benchmark presented in Section 2.6.

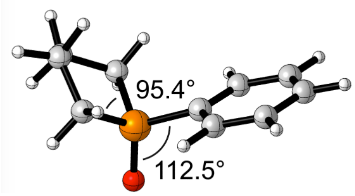

4

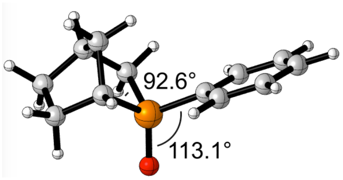

7

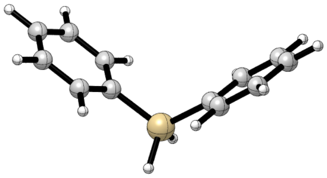

Ph_2SiH_2

Figure S1. Optimized structures of the phosphine oxides **4** and **7** and Ph_2SiH_2, determined using the SMD(CH3CN)-B3LYP /6-31G(d) method in the implicit solvent CH_3CN.

Compound **4**
P 0.878470 -0.023149 0.700086
C 1.749194 -1.380544 -0.208922
O 1.018366 -0.058895 2.209635
C 1.761080 1.357957 -0.163135
H 1.114684 1.795090 -0.931499
H 2.023936 2.145576 0.549627
H 2.441989 -1.833253 0.511063
H 1.074642 -2.165228 -0.561008
C 2.521661 -0.684137 -1.347573
H 3.363775 -1.297243 -1.687190
C 2.986323 0.685213 -0.816326
H 3.405305 1.310972 -1.611898
H 1.856684 -0.535219 -2.208064
H 3.774079 0.539321 -0.065823
C -0.871054 0.001938 0.159605
C -1.538638 1.221301 -0.041267
C -1.596752 -1.195663 0.048297
C -2.898219 1.241616 -0.358012
H -1.000731 2.161163 0.049833
C -2.955851 -1.174296 -0.269842
H -1.104765 -2.150748 0.211381
C -3.608103 0.044039 -0.475575
H -3.402014 2.191886 -0.513895
H -3.503837 -2.108597 -0.358592
H -4.665565 0.060131 -0.725785
Sum of electronic and thermal Free Energies= -805.345445
SCF Energies= -805.51815
Compound **7**
P 0.458073 0.087410 0.760871
O 0.616758 -0.011116 2.264192
C -1.286562 0.019143 0.217853
C -2.045883 1.193608 0.093113
C -1.908882 -1.222264 0.006736
C -3.398729 1.128293 -0.244882
H -1.585672 2.163841 0.259932
C -3.261243 -1.285468 -0.334056
H -1.342291 -2.143809 0.107679
C -4.007433 -0.110801 -0.461905
H -3.975630 2.044153 -0.341524
H -3.731569 -2.251017 -0.500095
H -5.059547 -0.160848 -0.729471
C 1.263223 1.542558 -0.072838
C 2.264780 0.881604 -1.055785
C 1.395390 -1.128331 -0.271698
H 1.751455 2.173753 0.676551
H 0.529555 2.150566 -0.609913
C 1.523349 -0.358678 -1.606730
H 2.131838 -0.917745 -2.327751
H 0.562430 -0.119041 -2.076569
C 3.438487 0.231133 -0.280806
H 4.279896 0.049237 -0.957786
H 3.800912 0.876345 0.526791
C 2.855450 -1.120271 0.249081
H 3.394362 -1.973703 -0.176236
H 2.908594 -1.206599 1.338433
H 2.598722 1.588474 -1.820953
H 0.940119 -2.118980 -0.324183
Sum of electronic and thermal Free Energies= -882.749786
SCF Energies= -882.957595
Ph2SiH2
Si 0.000000 0.000000 1.529666
H 1.216383 0.000000 2.389403
H -1.216383 0.000000 2.389403
C 0.000000 1.580863 0.504829
C 1.206601 2.185049 0.102507
C -1.206601 2.185049 0.102507
C 1.208914 3.344048 -0.677079
H 2.158490 1.752017 0.403347
C -1.208914 3.344048 -0.677079
H -2.158490 1.752017 0.403347
C 0.000000 3.924881 -1.069108
H 2.152212 3.795070 -0.974809
H -2.152212 3.795070 -0.974809
H 0.000000 4.828354 -1.673599
C 0.000000 -1.580863 0.504829
C -1.206601 -2.185049 0.102507
C 1.206601 -2.185049 0.102507
C -1.208914 -3.344048 -0.677079
H -2.158490 -1.752017 0.403347
C 1.208914 -3.344048 -0.677079
H 2.158490 -1.752017 0.403347
C 0.000000 -3.924881 -1.069108
H -2.152212 -3.795070 -0.974809

H 2.152212 -3.795070 -0.974809
H 0.000000 -4.828354 -1.673599

Sum of electronic and thermal Free Energies= -753.853436

SCF Energies= -754.016389

2.4 Transition State Geometries and Relative Free Energies for Reduction of the Phosphine Oxide 4

A conformational search on the TS structure was performed for reduction of the phosphine oxide **4**. TS structure optimization and frequency calculation were conducted using the B3LYP/6-31G(d) method in the gas phase. Single-point energies were calculated using the B3LYP-D3/6-311+G(d,p) method in the gas phase. Nine geometries for the TS of substrate **4** were considered, as displayed in Figure S2. Each featured a P–H–Si–O four-center TS with P and Si being centers of two trigonal bipyramids. Hydride and oxygen were placed at the axial and equatorial positions, respectively, of both trigonal bipyramids. This arrangement is consistent with the optimal TS structure reported by Krenske.[1] In Figure S2, the configurations are labeled "TS_XY," where X and Y indicate the indexes for various arrangements of ligands centered on Si and P, respectively. In addition, a TS was also searched for with hydride in the equatorial position as the initial input. Nevertheless, the optimized structures all ended up with hydride in the axial position.

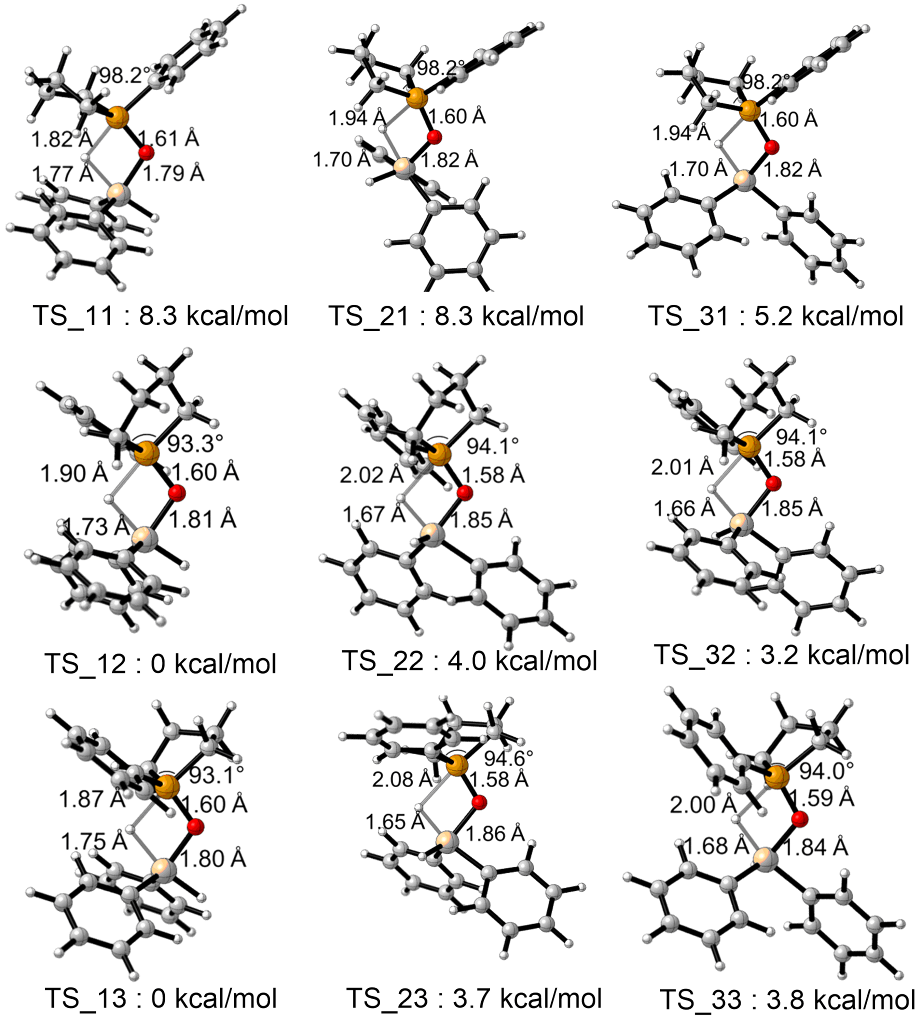

Figure S2. Nine geometries considered for the TSs of the reduction of the phosphine oxide **4** and their relative free energies (in kcal/mol).

TS1_phenyl11
P 1.283995 -0.043295 0.373769
C 1.293867 -1.522063 1.496101
O 0.623760 0.002117 -1.098132
Si -1.154385 0.023784 -0.921065
C 1.275519 1.280161 1.680153
H -1.320133 0.037262 -2.420364
H 2.272889 1.719391 1.774421
H 0.558470 2.067922 1.439336
H 0.327548 -2.025810 1.445272
H 2.074847 -2.236131 1.225239
H -0.504454 -0.012750 0.722944
C 1.559007 -0.895332 2.887491
H 1.191887 -1.552664 3.683267
C 0.903134 0.500702 2.960667
H 1.235335 1.045533 3.851243
H 2.641753 -0.790707 3.032074
H -0.184587 0.399067 3.025224
C -2.009095 1.669996 -0.502777
C -2.380223 2.525104 -1.556265
C -2.331952 2.077094 0.804637
C -3.035280 3.735526 -1.317336
H -2.152552 2.236173 -2.579581
C -2.996698 3.278545 1.052559
H -2.063017 1.437083 1.642327
C -3.346745 4.114167 -0.010979
H -3.304780 4.380532 -2.150320
H -3.242194 3.564727 2.072732
H -3.859974 5.053759 0.178768
C -2.074504 -1.594903 -0.530566
C -2.436300 -1.995191 0.768795
C -2.454900 -2.432323 -1.594642
C -3.147331 -3.173423 0.998924
H -2.160319 -1.367406 1.613659
C -3.155222 -3.620673 -1.373287
H -2.199177 -2.146766 -2.612331
C -3.504680 -3.992970 -0.074736
H -3.422894 -3.454299 2.012868
H -3.430720 -4.253154 -2.213897
H -4.053262 -4.915119 0.101131
C 3.039489 -0.000312 -0.257598
C 3.741810 1.208495 -0.377774
C 3.664660 -1.174225 -0.707879
C 5.033447 1.241816 -0.906209
H 3.279606 2.143619 -0.073745
C 4.956825 -1.145785 -1.232661
H 3.138343 -2.124027 -0.668412
C 5.647825 0.062993 -1.328612
H 5.556776 2.190705 -0.989272
H 5.420986 -2.068223 -1.571539
H 6.655104 0.086384 -1.735646
Sum of electronic and thermal Free Energies= -1559.106681
SCF Energies= -1559.463792
TS1_phenyl21
P 1.523374 -0.505355 -0.503954
C 2.216834 -0.868609 -2.182879
O 0.354833 0.557464 -0.255037
Si -1.206896 -0.093376 -0.921605
C 1.643322 -2.213341 0.208439
H 2.475854 -2.261328 0.916956
H 0.720188 -2.490033 0.721791
H 1.414187 -0.842002 -2.921530
H 2.983509 -0.144104 -2.468818
H -0.070700 -1.310349 -1.258728
C 2.807364 -2.289780 -2.010606
H 2.888870 -2.793967 -2.979412
C 1.930466 -3.095494 -1.027437
H 2.429316 -4.023461 -0.727282
H 3.823813 -2.207862 -1.604966
H 0.986016 -3.366571 -1.507383
C -2.166179 -1.327271 0.171985
C -2.957707 -0.904172 1.255160
C -2.121052 -2.709510 -0.092073
C -3.658090 -1.818219 2.046697
H -3.033166 0.155795 1.480915
C -2.833967 -3.627386 0.680116
H -1.518662 -3.069949 -0.923788
C -3.600832 -3.182267 1.759650
H -4.254695 -1.462841 2.883639
H -2.789989 -4.688176 0.443662
H -4.151052 -3.894232 2.370189
C 2.870975 0.450782 0.333724
C 3.241645 0.189845 1.661412
C 3.493612 1.518520 -0.333567
C 4.219248 0.957718 2.296527
H 2.759903 -0.606952 2.220701
C 4.473293 2.284079 0.297483
H 3.204212 1.770818 -1.349998
C 4.842387 2.002278 1.613987
H 4.490601 0.738934 3.325742
H 4.943942 3.104161 -0.237967

H 5.606691 2.597511 2.106105
H -1.531869 -0.172382 -2.388952
C -2.083400 1.592847 -0.590813
C -1.769621 2.474140 0.461807
C -3.141766 1.964519 -1.438464
C -2.482225 3.658562 0.662822
H -0.945491 2.236450 1.129533
C -3.863159 3.145066 -1.246059
H -3.405766 1.318793 -2.275282
C -3.534293 3.997114 -0.190823
H -2.213895 4.320760 1.483376
H -4.676441 3.402536 -1.921181
H -4.089250 4.919795 -0.037779
Sum of electronic and thermal Free Energies= -1559.102075
SCF Energies= -1559.457845
TS1_phenyl31
P -1.519381 -0.536121 -0.467823
C -1.600752 -2.167735 0.406893
O -0.372731 0.557907 -0.253088
Si 1.196172 -0.070298 -0.925188
C -2.173108 -1.084633 -2.113182
H -3.207045 -0.744467 -2.229495
H -1.572319 -0.678343 -2.929889
H -0.591211 -2.550274 0.562478
H -2.096666 -2.081467 1.376735
H 0.079983 -1.309830 -1.242647
C -2.421222 -3.043141 -0.573879
H -2.209605 -4.105070 -0.409701
C -2.112080 -2.625763 -2.028388
H -2.826503 -3.077400 -2.725358
H -3.490518 -2.894949 -0.376238
H -1.111346 -2.964353 -2.310718
C -2.899986 0.442563 0.284834
C -3.728674 1.254790 -0.504408
C -3.090042 0.451150 1.676044
C -4.730481 2.034367 0.075265
H -3.590182 1.298448 -1.581053
C -4.092366 1.227641 2.257345
H -2.444818 -0.139054 2.320855
C -4.919044 2.017926 1.457420
H -5.360725 2.656251 -0.554662
H -4.223794 1.217698 3.335970
H -5.701338 2.621612 1.909297
C 2.049958 1.627438 -0.594559
C 1.727482 2.503381 0.459710
C 3.099426 2.015113 -1.446151
C 2.423343 3.698090 0.658663
H 0.909045 2.253701 1.130088
C 3.803984 3.206098 -1.256034
H 3.369598 1.373972 -2.284554
C 3.466810 4.052641 -0.198963
H 2.148275 4.356021 1.480416
H 4.610448 3.476037 -1.934452
H 4.008464 4.983491 -0.047774
C 2.191169 -1.293609 0.149388
C 2.988545 -0.863621 1.225449
C 2.168621 -2.674496 -0.124630
C 3.716745 -1.770038 2.000544
H 3.047422 0.196019 1.457758
C 2.909432 -3.584330 0.630745
H 1.560694 -3.040015 -0.950323
C 3.681968 -3.132725 1.703511
H 4.317649 -1.409624 2.832253
H 2.882885 -4.643920 0.386439
H 4.253885 -3.838514 2.301125
H 1.505518 -0.139904 -2.395566
Sum of electronic and thermal Free Energies= -1559.102142
SCF Energies= -1559.457844
TS1_phenyl12
P -0.737467 -1.509379 0.515324
C -1.457720 -2.904620 1.526221
O 0.071155 -0.728635 1.653342
Si 1.097534 0.533992 0.857739
C 0.052734 -2.661455 -0.705806
H 1.641781 0.907493 2.218574
H -0.321968 -2.398798 -1.700461
H 1.126740 -2.460255 -0.717401
H -0.735497 -3.151035 2.313388
H -2.396165 -2.616830 2.007975
H 0.230012 -0.186015 -0.449992
C -1.603115 -4.070317 0.536982
H -1.781142 -5.022201 1.049208
C -0.301853 -4.106758 -0.290282
H -0.392698 -4.757400 -1.166759
H -2.463477 -3.897871 -0.124030
H 0.506412 -4.513788 0.329128
C 0.388015 2.230141 0.366707
C 0.512189 3.304577 1.266274
C -0.219220 2.490564 -0.874511
C 0.054098 4.583692 0.942384

H 0.977321 3.134625 2.234652
C -0.672686 3.766386 -1.210098
H -0.341991 1.676794 -1.585385
C -0.538466 4.817288 -0.299345
H 0.160190 5.396493 1.657224
H -1.134827 3.942214 -2.178883
H -0.895476 5.811865 -0.556372
C 2.729809 -0.016470 0.041764
C 2.860141 -0.255588 -1.338499
C 3.883040 -0.155453 0.834489
C 4.084387 -0.612661 -1.905218
H 1.985719 -0.153634 -1.978300
C 5.109965 -0.526247 0.278768
H 3.817268 0.033523 1.903546
C 5.213388 -0.753830 -1.094206
H 4.160212 -0.781166 -2.977114
H 5.984907 -0.633458 0.915765
H 6.167687 -1.037854 -1.531285
C -2.228956 -0.660242 -0.100647
C -2.823413 -0.960060 -1.333934
C -2.821971 0.297871 0.734960
C -3.994202 -0.309180 -1.724842
H -2.380925 -1.695216 -1.999683
C -3.989721 0.948207 0.337025
H -2.362879 0.544798 1.687633
C -4.578862 0.645727 -0.891489
H -4.446517 -0.548148 -2.683512
H -4.434758 1.696212 0.987044
H -5.488124 1.154099 -1.200074

Sum of electronic and thermal Free Energies= -1559.115356

SCF Energies= -1559.471055

TS1_phenyl22

P -1.457475 -1.278731 -0.074380
C -2.158765 -2.602358 1.025802
O 0.068303 -1.250618 0.352243
Si 1.035484 -0.260744 -0.877272
C -1.998364 -2.106404 -1.638849
H -2.653489 -1.415196 -2.178487
H -1.118524 -2.257652 -2.269314
H -1.352415 -3.315189 1.233449
H -2.508858 -2.198531 1.979522
H -0.537580 -0.056824 -1.393032
C -3.267371 -3.260846 0.187112
H -3.578150 -4.222272 0.609913
C -2.704437 -3.423177 -1.239820
H -3.486156 -3.685127 -1.960692
H -4.157633 -2.617833 0.169200
H -1.974091 -4.241161 -1.246030
C 1.206467 1.631602 -0.669455
C 2.266089 2.213790 0.051627
C 0.283102 2.505603 -1.273677
C 2.395859 3.600071 0.166147
H 3.004615 1.575371 0.527113
C 0.414334 3.891152 -1.177740
H -0.555772 2.087465 -1.825130
C 1.471775 4.443497 -0.451460
H 3.223017 4.020546 0.733587
H -0.311053 4.540378 -1.663045
H 1.573710 5.523040 -0.367627
C -2.415961 0.208268 0.347820
C -3.670322 0.479575 -0.217284
C -1.899849 1.067659 1.328843
C -4.394898 1.599515 0.191506
H -4.088288 -0.170405 -0.980484
C -2.628956 2.185702 1.732437
H -0.923840 0.871839 1.761344
C -3.876239 2.453598 1.166216
H -5.363847 1.804611 -0.255303
H -2.215737 2.851421 2.484681
H -4.440983 3.326875 1.480554
C 2.666241 -0.933815 -0.078590
C 2.859979 -1.154537 1.298735
C 3.759785 -1.195286 -0.922031
C 4.080464 -1.604286 1.806764
H 2.037844 -0.980507 1.988984
C 4.987106 -1.643727 -0.426591
H 3.649006 -1.050723 -1.996108
C 5.150969 -1.849780 0.943522
H 4.196835 -1.767379 2.876303
H 5.812255 -1.836213 -1.109040
H 6.102132 -2.202009 1.335937
H 1.323876 -0.783091 -2.262936

Sum of electronic and thermal Free Energies= -1559.108886

SCF Energies= -1559.463563

TS1_phenyl32

P -1.535436 -0.323738 0.703217
C -2.279702 -0.216736 2.404018
O -0.162201 0.447395 0.877270
Si 0.851750 0.254341 -0.661380
C -1.639101 -2.170735 0.629615

H -2.260968 -2.438474 -0.230952
H -0.640653 -2.566144 0.428701
H -1.453010 -0.194832 3.123716
H -2.866112 0.696516 2.535519
H -0.535819 -0.590229 -1.024763
C -3.099771 -1.508795 2.552878
H -3.371671 -1.698570 3.596802
C -2.236491 -2.647672 1.973249
H -2.810894 -3.570997 1.843136
H -4.038621 -1.424931 1.988787
H -1.421429 -2.871740 2.671875
C -2.728636 0.535181 -0.367680
C -3.849628 -0.101491 -0.918112
C -2.535917 1.907445 -0.588771
C -4.761120 0.624245 -1.685360
H -4.017569 -1.163117 -0.762157
C -3.449794 2.626439 -1.358868
H -1.669756 2.408753 -0.166959
C -4.562844 1.987891 -1.908109
H -5.624256 0.120810 -2.112067
H -3.287690 3.686638 -1.531666
H -5.272131 2.549846 -2.509286
C 2.107819 1.548317 0.039660
C 2.428981 1.718666 1.400517
C 2.791223 2.363234 -0.879697
C 3.386782 2.645034 1.818999
H 1.913223 1.121942 2.148616
C 3.752712 3.292394 -0.474319
H 2.561861 2.274019 -1.940885
C 4.054044 3.435578 0.880504
H 3.610954 2.753808 2.878219
H 4.262135 3.906817 -1.213645
H 4.798780 4.159267 1.203668
C 1.879811 -1.350206 -0.791934
C 3.119540 -1.508306 -0.145661
C 1.423148 -2.424360 -1.579913
C 3.862366 -2.685152 -0.270759
H 3.514818 -0.697283 0.459409
C 2.167286 -3.595953 -1.725300
H 0.466812 -2.335075 -2.091832
C 3.389979 -3.731612 -1.063587
H 4.814145 -2.781303 0.246650
H 1.794271 -4.403339 -2.351598
H 3.969842 -4.645682 -1.167153
H 0.644290 0.959008 -1.977448
Sum of electronic and thermal Free Energies= -1559.109814
SCF Energies= -1559.464818
TS1_phenyl13
P -0.617211 -1.576054 -0.460756
C 0.330406 -2.623119 0.741039
O 0.123289 -0.748729 -1.619451
Si 1.008501 0.624629 -0.858103
C -1.165025 -3.057734 -1.476788
H 1.538203 1.019239 -2.217463
H -2.177109 -3.343269 -1.165000
H -1.192700 -2.811299 -2.540685
H 1.382114 -2.353719 0.594393
H 0.077779 -2.340603 1.766647
H 0.185947 -0.161485 0.466841
C 0.064345 -4.098820 0.392673
H 0.897721 -4.733516 0.713536
C -0.170063 -4.177405 -1.128232
H -0.541167 -5.163448 -1.429410
H -0.832114 -4.452963 0.918439
H 0.777685 -4.012524 -1.656961
C 2.670285 0.273284 0.005870
C 3.849371 0.259343 -0.760911
C 2.797417 0.072227 1.392527
C 5.099038 0.045911 -0.174179
H 3.785615 0.422132 -1.834337
C 4.042486 -0.128492 1.989610
H 1.903518 0.077960 2.012936
C 5.198074 -0.146645 1.204383
H 5.994286 0.033592 -0.791519
H 4.113951 -0.270947 3.065564
H 6.169295 -0.308519 1.665823
C 0.114277 2.244472 -0.416312
C -0.531419 2.469140 0.812513
C 0.133570 3.303489 -1.342295
C -1.123856 3.696433 1.110213
H -0.575078 1.664420 1.542560
C -0.463818 4.533237 -1.056179
H 0.626574 3.161451 -2.301291
C -1.092952 4.732478 0.173456
H -1.613510 3.845467 2.069903
H -0.436899 5.334672 -1.790891
H -1.557897 5.688848 0.401165
C -2.191478 -0.891287 0.146018
C -2.871210 0.038730 -0.652125
C -2.760595 -1.312712 1.355752
C -4.104070 0.544533 -0.238330

H -2.431547 0.377401 -1.585244
C -3.993977 -0.804215 1.763119
H -2.248610 -2.034251 1.986864
C -4.667369 0.124957 0.967381
H -4.620042 1.272091 -0.858421
H -4.427050 -1.133353 2.703837
H -5.626752 0.521618 1.287894
Sum of electronic and thermal Free Energies= -1559.115092
SCF Energies= -1559.470997
TS1_phenyl23
P -1.562475 0.335950 0.723817
C -1.516122 2.184954 0.748146
O -0.201251 -0.466852 0.826282
Si 0.801161 -0.268420 -0.732874
C -2.235515 0.182731 2.449985
H -3.323401 0.057793 2.408013
H -1.805858 -0.683519 2.958703
H -0.458442 2.462306 0.694066
H -2.001124 2.601611 -0.137456
H -0.526258 0.648086 -1.056739
C -2.161959 2.625250 2.078863
H -1.773543 3.598737 2.396841
C -1.881607 1.525062 3.119219
H -2.455125 1.678766 4.039637
H -3.246174 2.736309 1.948632
H -0.818846 1.534297 3.392059
C 1.926344 1.277552 -0.787402
C 3.184140 1.320504 -0.158476
C 1.526577 2.423165 -1.501792
C 3.998579 2.453609 -0.230979
H 3.536021 0.452173 0.390985
C 2.340847 3.552713 -1.595030
H 0.557692 2.424446 -1.997771
C 3.580920 3.572490 -0.952404
H 4.963309 2.459162 0.271251
H 2.009125 4.417505 -2.165611
H 4.216229 4.452835 -1.015423
C -2.839668 -0.375096 -0.363835
C -3.446145 -1.580703 0.021875
C -3.231970 0.239970 -1.560842
C -4.431642 -2.159481 -0.778323
H -3.143599 -2.077236 0.939699
C -4.231543 -0.330191 -2.346693
H -2.736798 1.147787 -1.889601
C -4.830349 -1.531199 -1.958587
H -4.886346 -3.099228 -0.478053
H -4.532399 0.155645 -3.270553
H -5.600915 -1.979734 -2.579531
H 0.586928 -0.902090 -2.086205
C 1.981588 -1.662533 -0.084900
C 2.308907 -1.892849 1.265792
C 2.602851 -2.490090 -1.036118
C 3.213416 -2.887303 1.644207
H 1.840684 -1.287771 2.038001
C 3.510494 -3.487895 -0.671192
H 2.366270 -2.355409 -2.090822
C 3.819455 -3.689343 0.674310
H 3.443909 -3.040237 2.696705
H 3.971661 -4.110325 -1.435164
H 4.522289 -4.466362 0.966126
Sum of electronic and thermal Free Energies= -1559.109690
SCF Energies= -1559.46417
TS1_phenyl33
P 1.466094 -1.277062 -0.062722
C 1.931863 -2.130232 -1.637119
O -0.062458 -1.214628 0.365643
Si -1.000905 -0.205904 -0.855336
C 2.094019 -2.656603 1.023607
H 3.103096 -2.402505 1.369570
H 1.453296 -2.781478 1.899676
H 0.985349 -2.354446 -2.141559
H 2.484103 -1.449502 -2.289848
H 0.589342 -0.026905 -1.358541
C 2.689717 -3.417105 -1.251622
H 2.583141 -4.182330 -2.028085
C 2.137155 -3.892317 0.106163
H 2.748569 -4.694411 0.533793
H 3.762757 -3.204980 -1.154730
H 1.123587 -4.291726 -0.025658
C -1.132666 1.691020 -0.659422
C -0.202118 2.543278 -1.283510
C -2.170857 2.297833 0.072477
C -0.306688 3.931643 -1.196445
H 0.620793 2.105399 -1.843618
C -2.272779 3.687156 0.179213
H -2.914672 1.676682 0.562425
C -1.342695 4.508723 -0.458403
H 0.422884 4.563792 -1.697722
H -3.083302 4.127033 0.755849
H -1.423421 5.590542 -0.381246

C	2.483153	0.164667	0.364947
C	1.977398	1.117073	1.259535
C	3.788792	0.299435	-0.129275
C	2.770574	2.196796	1.648399
H	0.963859	1.024445	1.635575
C	4.574962	1.383150	0.260893
H	4.197399	-0.433473	-0.820497
C	4.067239	2.332459	1.150526
H	2.368703	2.936233	2.335060
H	5.583174	1.485500	-0.131034
H	4.680920	3.176539	1.452742
C	-2.654266	-0.835671	-0.069080
C	-2.859891	-1.060770	1.305816
C	-3.751402	-1.059688	-0.918781
C	-4.094965	-1.478807	1.805798
H	-2.035529	-0.916417	2.000281
C	-4.992921	-1.476590	-0.431475
H	-3.632366	-0.910526	-1.991381
C	-5.168372	-1.687332	0.936570
H	-4.220402	-1.646168	2.873646
H	-5.820270	-1.640948	-1.118583
H	-6.130874	-2.014867	1.322653
H	-1.280723	-0.728583	-2.241259

Sum of electronic and thermal Free Energies= -1559.109142

SCF Energies= -1559.46401

2.5 Transition State Geometries and Relative Free Energies for Reduction of the Phosphine Oxide 7

A conformational search was performed on the TS structure for reduction of the phosphine oxide **7**. TS structure optimization and frequency calculation were conducted using the B3LYP/6-31G(d) method in the gas phase. Single-point energies were calculated using the B3LYP-D3/6-311+G(d,p) method in the gas phase. Nine geometries for the TS of the diphenysilane-mediated reduction of substrate **7** were considered, as displayed in Figure S3. Each featured a P–H–Si–O four-center TS with P and Si being centers of two trigonal bipyramids. Hydride and oxygen were placed in the axial and equatorial positions, respectively, for both trigonal bipyramids, consistent with the optimal transition structure reported by Krenske.[1] In Figure S3, the configurations are labeled "P4_TS_XY," where X and Y indicate indexes for various arrangements of ligands centered on Si and P, respectively. In addition, TSs were searched for with hydride in the equatorial position as the initial input, but the optimized structures all ended up with hydride in the axial position.

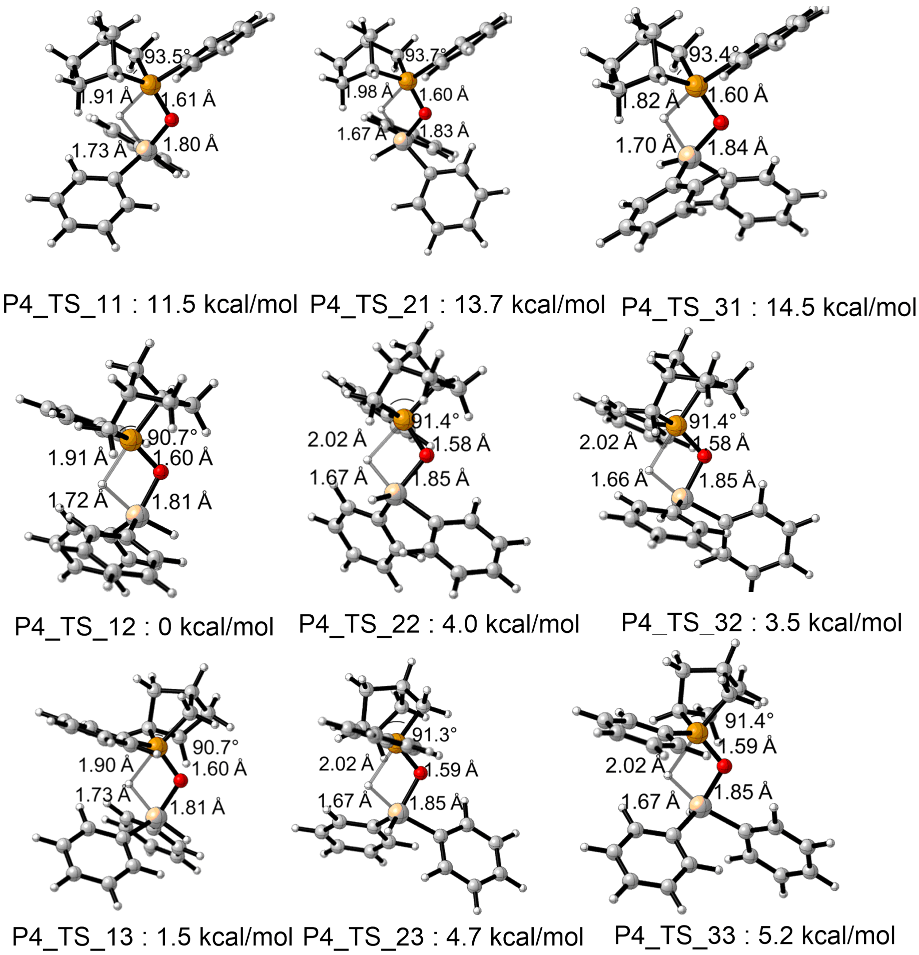

Figure S3. Nine geometries considered for the TS of the reduction of the phosphine oxide **7** and their relative free energies (in kcal/mol).

P4_TS1_phenyl11

P	-1.289736	0.011603	0.216712
O	-0.561445	-0.002798	-1.219818
Si	1.221465	-0.075710	-1.020812
H	0.581953	0.029965	0.588277
C	-2.998600	-0.036385	-0.529680
C	-3.629859	-1.255716	-0.814999
C	-3.641519	1.149874	-0.916408
C	-4.875209	-1.289604	-1.444882
H	-3.147515	-2.195953	-0.562029
C	-4.887645	1.119314	-1.542788
H	-3.168920	2.112063	-0.739886
C	-5.511064	-0.101521	-1.804860
H	-5.345036	-2.246472	-1.656474
H	-5.368146	2.050608	-1.830740
H	-6.482085	-0.126452	-2.291919
C	-1.325969	-1.354377	1.489804
C	-1.526382	-0.555281	2.811103
C	-1.513084	1.371386	1.501599
H	-0.404012	-1.937254	1.471265
H	-2.173368	-2.023798	1.318549
C	-2.476854	0.613252	2.456241
H	-2.704295	1.233196	3.331624
H	-3.421357	0.300494	2.004134
C	-0.245302	0.210373	3.236634
H	-0.281488	0.423408	4.310733
H	0.660676	-0.372403	3.049815
C	-0.278538	1.534271	2.407466
H	-0.444281	2.398886	3.062330
H	0.639795	1.711742	1.848461
H	-1.902896	-1.216720	3.597745
H	-1.894313	2.301666	1.077510
C	2.071050	-1.720775	-0.573185
C	2.449294	-2.591017	-1.611739

C 2.387048 -2.110755 0.740952
C 3.106451 -3.796033 -1.352244
H 2.225652 -2.316838 -2.639915
C 3.053318 -3.307025 1.010395
H 2.110956 -1.461195 1.568729
C 3.411761 -4.156298 -0.039096
H 3.382289 -4.451873 -2.174702
H 3.292493 -3.578473 2.036141
H 3.925722 -5.092173 0.166484
C 2.219983 1.519194 -0.743505
C 2.915093 1.801671 0.446378
C 2.319863 2.453592 -1.789106
C 3.673974 2.963891 0.589600
H 2.872595 1.094271 1.272005
C 3.057996 3.631389 -1.646780
H 1.816530 2.252740 -2.732445
C 3.739556 3.887693 -0.456514
H 4.213790 3.150718 1.515117
H 3.109192 4.342744 -2.467818
H 4.323485 4.798130 -0.345242
H 1.347524 -0.159431 -2.524851
Sum of electronic and thermal Free Energies= -1636.472667
SCF Energies= -1636.864173
P4_TS1_phenyl21
P 1.442947 -0.259848 -0.265274
O 0.146796 0.673524 -0.211725
Si -1.319840 -0.221327 -0.835503
H -0.066960 -1.315330 -0.990739
C 2.576390 0.967249 0.538730
C 2.832009 0.939104 1.917026
C 3.123470 2.014437 -0.220422
C 3.627041 1.917453 2.517438
H 2.399872 0.162325 2.541406
C 3.922211 2.988868 0.376594
H 2.918649 2.083623 -1.285081
C 4.179343 2.941005 1.747988
H 3.810778 1.878513 3.587736
H 4.337939 3.789423 -0.229264
H 4.801095 3.700537 2.213963
C -2.351499 1.409764 -0.773961
C -2.160222 2.458610 0.146325
C -3.405157 1.554883 -1.693566
C -2.985439 3.585541 0.155276
H -1.343260 2.399215 0.860726
C -4.237779 2.676434 -1.693687
H -3.576335 0.774403 -2.434145
C -4.029810 3.697198 -0.765182
H -2.810662 4.380365 0.877485
H -5.043638 2.756387 -2.420175
H -4.671872 4.575010 -0.762045
C 1.759841 -1.866588 0.630543
C 2.641659 -2.633208 -0.400612
C 2.428853 -0.767563 -1.778045
H 0.826398 -2.377433 0.870724
H 2.320562 -1.695958 1.554147
C 3.549392 -1.562107 -1.052636
H 4.236895 -2.001108 -1.784893
H 4.137112 -0.978545 -0.338700
C 1.825086 -3.129777 -1.624115
H 2.351743 -3.960588 -2.106627
H 0.834408 -3.489758 -1.335693
C 1.745653 -1.895436 -2.578770
H 2.328805 -2.070134 -3.491682
H 0.723286 -1.655317 -2.872613
H 3.187080 -3.440154 0.098448
H 2.757619 0.085247 -2.374774
C -2.187424 -1.364462 0.420657
C -2.996697 -0.861121 1.454754
C -2.047372 -2.762579 0.334493
C -3.622488 -1.711836 2.369916
H -3.146466 0.211448 1.542728
C -2.685123 -3.620818 1.230995
H -1.431434 -3.185683 -0.457009
C -3.470740 -3.094267 2.259226
H -4.235603 -1.293798 3.164976
H -2.568603 -4.697652 1.131010
H -3.963066 -3.757834 2.966116
H -1.642322 -0.553812 -2.269880
Sum of electronic and thermal Free Energies= -1636.469544
SCF Energies= -1636.859902
P4_TS1_phenyl31
P -1.312578 -0.067586 -0.531674
O 0.022229 0.651729 -0.007153
Si 1.445704 -0.239261 -0.754215
H 0.038055 -0.953742 -1.378481
C -2.436961 1.020124 0.487985
C -2.823659 2.283592 0.016307
C -2.834926 0.645753 1.780424
C -3.599706 3.137584 0.801153
H -2.507306 2.620603 -0.967383

C -3.612696 1.496678 2.567186
H -2.534529 -0.314165 2.190985
C -4.001376 2.744261 2.078186
H -3.886556 4.111879 0.414294
H -3.910982 1.184170 3.564491
H -4.608009 3.406951 2.689401
C 2.555297 1.316758 -0.663085
C 2.342108 2.400078 0.212131
C 3.696441 1.367860 -1.482899
C 3.232716 3.472440 0.272848
H 1.461709 2.405315 0.847569
C 4.594399 2.436340 -1.428303
H 3.885452 0.554763 -2.181561
C 4.363568 3.493105 -0.547303
H 3.044133 4.295722 0.958406
H 5.468559 2.446416 -2.075460
H 5.056790 4.329845 -0.502370
C 1.872165 -1.503249 0.585781
C 1.536685 -1.294611 1.936375
C 2.561308 -2.685637 0.261387
C 1.873324 -2.225926 2.920402
H 1.009601 -0.387042 2.221543
C 2.897502 -3.623770 1.239838
H 2.841166 -2.874860 -0.772894
C 2.553739 -3.395022 2.573347
H 1.607417 -2.039299 3.958392
H 3.429600 -4.530739 0.962501
H 2.816767 -4.121928 3.337976
H 2.057539 -0.792799 -2.026106
C -2.019076 0.071045 -2.255888
C -2.785130 -1.274798 -2.399415
C -2.093778 -1.760018 -0.226036
H -1.229120 0.192883 -3.000314
H -2.709287 0.916456 -2.327717
C -3.416205 -1.541606 -1.010887
H -4.015631 -2.459758 -1.005043
H -4.040018 -0.723978 -0.640475
C -1.819612 -2.483679 -2.542305
H -2.334898 -3.314970 -3.035791
H -0.942355 -2.236614 -3.147321
C -1.433874 -2.861821 -1.076014
H -1.876542 -3.825236 -0.793632
H -0.356436 -2.935515 -0.921394
H -3.509606 -1.214341 -3.217605
H -2.198036 -2.003295 0.832021

Sum of electronic and thermal Free Energies= -1636.468170

SCF Energies= -1636.858786

P4_TS1_phenyl12

P 0.426879 -1.416218 -0.265052
O -0.210035 -0.584803 -1.473512
Si -0.884449 0.960090 -0.807050
H -0.193931 0.183391 0.566782
C 2.087375 -0.868824 0.251462
C 2.553340 -0.969314 1.569066
C 2.928959 -0.343269 -0.739553
C 3.847609 -0.559123 1.888015
H 1.910382 -1.355840 2.354326
C 4.219934 0.073151 -0.413651
H 2.569181 -0.243341 -1.759685
C 4.682813 -0.037431 0.898016
H 4.200599 -0.642311 2.912309
H 4.861059 0.488690 -1.185937
H 5.688625 0.286932 1.150081
C -0.592602 -2.258894 1.043403
C -0.545103 -3.762965 0.684421
C 0.769797 -3.063443 -1.091616
H -1.598795 -1.831178 1.032370
H -0.160480 -2.043652 2.026481
C 0.880399 -3.998920 0.133298
H 1.038852 -5.041379 -0.168378
H 1.674699 -3.725654 0.837790
C -1.419810 -4.038243 -0.564038
H -1.648161 -5.107017 -0.636903
H -2.376039 -3.506148 -0.519311
C -0.530886 -3.574612 -1.763255
H -0.291124 -4.421314 -2.416911
H -1.007008 -2.809397 -2.379559
H -0.813539 -4.383801 1.545077
H 1.637141 -3.036843 -1.755251
C 0.220419 2.475620 -0.482648
C 0.313633 3.471784 -1.471554
C 0.917047 2.689013 0.719887
C 1.063937 4.632347 -1.269614
H -0.212833 3.334909 -2.413390
C 1.664452 3.847509 0.933073
H 0.875136 1.930654 1.498089
C 1.740261 4.823233 -0.063874
H 1.120229 5.386351 -2.051414
H 2.190897 3.989915 1.874195
H 2.325138 5.725636 0.098291
C -2.589865 0.900701 0.043477

C -2.757433 0.794998 1.436180
C -3.752069 1.003602 -0.741828
C -4.024303 0.795603 2.021540
H -1.876806 0.715835 2.070674
C -5.025098 0.992925 -0.166572
H -3.655524 1.095862 -1.821238
C -5.163633 0.890169 1.218234
H -4.125418 0.722566 3.102085
H -5.907444 1.067647 -0.798020
H -6.152628 0.885129 1.670210
H -1.347853 1.321090 -2.201400
Sum of electronic and thermal Free Energies= -1636.490851
SCF Energies= -1636.881983
P4_TS1_phenyl22
P -1.536589 -0.707643 -0.103742
O -0.035925 -0.990107 0.319814
Si 1.109051 -0.198369 -0.902669
H -0.391287 0.301989 -1.424351
C -2.155575 0.941837 0.344197
C -3.198891 1.580799 -0.339907
C -1.573679 1.568045 1.456465
C -3.657364 2.826474 0.088194
H -3.651424 1.120422 -1.212966
C -2.034241 2.815202 1.877720
H -0.750693 1.089887 1.979028
C -3.077346 3.445004 1.197484
H -4.464875 3.315285 -0.449866
H -1.570817 3.296620 2.734083
H -3.433450 4.417375 1.526088
C -2.239180 -1.435469 -1.659200
C -3.179250 -2.565284 -1.170227
C -2.521008 -1.891064 0.950640
H -1.415056 -1.787735 -2.286054
H -2.765412 -0.658032 -2.222600
C -3.822830 -2.018659 0.125751
H -4.513873 -2.738984 0.579624
H -4.363007 -1.075282 -0.014540
C -2.347040 -3.757478 -0.633668
H -2.965241 -4.660411 -0.591143
H -1.491243 -3.982450 -1.278742
C -1.912966 -3.310236 0.800531
H -2.348895 -3.969120 1.560130
H -0.830353 -3.315591 0.941868
H -3.891811 -2.852078 -1.949792
H -2.634724 -1.553520 1.983206
C 1.640894 1.623061 -0.675393
C 2.808084 1.978722 0.026939
C 0.891982 2.668022 -1.248508
C 3.207265 3.311579 0.151541
H 3.417047 1.202574 0.480417
C 1.290586 4.000532 -1.140131
H -0.022416 2.428960 -1.786064
C 2.450898 4.326933 -0.434725
H 4.112301 3.555698 0.703189
H 0.694075 4.785036 -1.600521
H 2.761941 5.365005 -0.342393
C 2.570877 -1.183900 -0.099496
C 2.716987 -1.421682 1.281049
C 3.583591 -1.679640 -0.938253
C 3.816541 -2.110785 1.796435
H 1.951277 -1.067947 1.967572
C 4.690174 -2.369805 -0.435614
H 3.503548 -1.527747 -2.014022
C 4.809605 -2.587664 0.937223
H 3.898742 -2.279732 2.868259
H 5.455247 -2.740360 -1.114607
H 5.666262 -3.126689 1.335169
H 1.304970 -0.757783 -2.290270
Sum of electronic and thermal Free Energies= -1636.485162
SCF Energies= -1636.874452
P4_TS1_pheny32
P 1.485888 0.260388 0.359769
O 0.098373 -0.414006 0.721653
Si -0.981619 -0.447898 -0.784859
H 0.403469 0.282364 -1.344424
C 2.583018 -0.837629 -0.586676
C 3.601871 -0.371384 -1.428597
C 2.403740 -2.219253 -0.417795
C 4.432800 -1.276090 -2.088834
H 3.746862 0.693352 -1.585107
C 3.236261 -3.118872 -1.083785
H 1.607375 -2.587822 0.222372
C 4.252365 -2.650033 -1.917606
H 5.217911 -0.905887 -2.742299
H 3.085845 -4.186725 -0.952636
H 4.898882 -3.352211 -2.436540
C 1.653905 2.072296 -0.006684
C 2.359151 2.658854 1.241855
C 2.320835 0.471718 2.014210
H 0.660840 2.490413 -0.192449

H 2.236977 2.202000 -0.924514
C 3.360802 1.568648 1.688970
H 3.922287 1.864886 2.583067
H 4.083111 1.289237 0.913493
C 1.375603 2.697560 2.438203
H 1.745724 3.381573 3.209228
H 0.383281 3.052042 2.140576
C 1.353578 1.225484 2.964077
H 1.741180 1.172893 3.987769
H 0.353152 0.787791 2.970359
H 2.808689 3.631960 1.021152
H 2.714288 -0.463726 2.417994
C -2.224942 -1.582762 0.172250
C -2.492203 -1.515717 1.553669
C -2.953635 -2.530220 -0.567775
C -3.440531 -2.341902 2.160490
H -1.942242 -0.808865 2.169907
C -3.906158 -3.362010 0.026542
H -2.767333 -2.624872 -1.636831
C -4.153033 -3.269254 1.396616
H -3.622182 -2.265873 3.230688
H -4.451066 -4.084339 -0.577556
H -4.890537 -3.915657 1.866759
C -1.981490 1.146741 -1.114194
C -3.193325 1.426590 -0.456817
C -1.533575 2.083836 -2.065165
C -3.917961 2.590328 -0.727239
H -3.580223 0.720713 0.272488
C -2.259758 3.239920 -2.354403
H -0.598798 1.897862 -2.590679
C -3.454634 3.499398 -1.678968
H -4.848540 2.783242 -0.198381
H -1.894462 3.939036 -3.103458
H -4.020404 4.402612 -1.894951
H -0.862810 -1.356674 -1.981837

Sum of electronic and thermal Free Energies= -1636.485130

SCF Energies= -1636.875578

P4_TS1_phenyl13

P -0.230799 -1.506270 -0.415891
O 0.279765 -0.532714 -1.582060
Si 0.674517 1.076978 -0.855968
H 0.161617 0.122985 0.487641
C -1.933631 -1.244174 0.180037
C -2.937259 -0.985429 -0.763023
C -2.262349 -1.313707 1.540024
C -4.255994 -0.798032 -0.347254
H -2.687293 -0.913656 -1.818199
C -3.584258 -1.142420 1.949141
H -1.488488 -1.489422 2.281818
C -4.582214 -0.882770 1.006885
H -5.026650 -0.583920 -1.082389
H -3.833379 -1.202723 3.005088
H -5.610074 -0.740720 1.329042
C -0.353651 -3.116778 -1.398502
C 0.604587 -4.085105 -0.667954
C 0.901957 -2.319709 0.816289
H -0.074689 -2.945211 -2.441408
H -1.380295 -3.496645 -1.374300
C 0.394632 -3.779104 0.831362
H 1.027462 -4.399549 1.478115
H -0.645127 -3.892169 1.158691
C 2.076908 -3.652208 -0.882375
H 2.756855 -4.480820 -0.656931
H 2.265105 -3.349872 -1.917493
C 2.284506 -2.475463 0.123086
H 3.023488 -2.735262 0.888795
H 2.632996 -1.556513 -0.352624
H 0.420597 -5.127597 -0.946531
H 0.939673 -1.792385 1.769037
C 2.357565 1.331719 0.003955
C 3.473783 1.706076 -0.765555
C 2.542116 1.206653 1.392850
C 4.720660 1.937092 -0.178943
H 3.361580 1.820131 -1.841379
C 3.779344 1.449239 1.989952
H 1.696992 0.919417 2.015327
C 4.875292 1.811053 1.202077
H 5.568905 2.218974 -0.798538
H 3.892118 1.355949 3.067848
H 5.842834 1.994428 1.663301
C -0.700065 2.337739 -0.478477
C -1.385984 2.398476 0.747346
C -1.023909 3.301632 -1.450494
C -2.345930 3.379213 0.997726
H -1.165822 1.660584 1.514899
C -1.988703 4.283050 -1.211980
H -0.509111 3.282713 -2.408395
C -2.651179 4.324600 0.015564
H -2.859162 3.406099 1.956376
H -2.221894 5.014799 -1.982117
H -3.402058 5.087811 0.206436

H 1.057112 1.570785 -2.234134
Sum of electronic and thermal Free Energies= -1636.488699
SCF Energies= -1636.879622
P4_TS1_phenyl23
P -1.528638 0.182743 0.497976
O -0.107921 -0.453748 0.810949
Si 0.926720 -0.459581 -0.719097
H -0.460899 0.320170 -1.214189
C -2.614186 -0.876213 -0.502772
C -2.595409 -2.255807 -0.251387
C -3.469404 -0.361464 -1.486048
C -3.428043 -3.108640 -0.977047
H -1.921287 -2.663692 0.497081
C -4.305981 -1.217507 -2.200739
H -3.474735 0.701710 -1.707461
C -4.286715 -2.591236 -1.947835
H -3.401576 -4.177494 -0.784592
H -4.966104 -0.812247 -2.962603
H -4.934938 -3.256614 -2.511268
C -2.302870 0.232031 2.209619
C -2.596111 1.732490 2.446339
C -1.804778 2.004009 0.274143
H -1.617290 -0.196517 2.945356
H -3.229375 -0.351488 2.219803
C -3.103694 2.243829 1.080008
H -3.351706 3.312200 1.098289
H -3.976560 1.695999 0.707323
C -1.275713 2.525824 2.617238
H -1.471294 3.492654 3.092987
H -0.558931 1.992588 3.249990
C -0.748417 2.721941 1.159774
H -0.732922 3.781741 0.884126
H 0.263636 2.340615 1.009217
H -3.296497 1.882023 3.273897
H -1.797181 2.313132 -0.770250
C 1.952013 1.112954 -1.082530
C 3.199910 1.356375 -0.479894
C 1.486019 2.068598 -2.005710
C 3.941068 2.503926 -0.774826
H 3.601482 0.635398 0.226314
C 2.228762 3.206302 -2.321965
H 0.522462 1.911886 -2.486808
C 3.459265 3.430710 -1.699529
H 4.899033 2.669250 -0.287065
H 1.848370 3.920116 -3.049427
H 4.037842 4.320730 -1.935648
C 2.191571 -1.618142 0.181446
C 2.502732 -1.568625 1.554337
C 2.885499 -2.565667 -0.590941
C 3.459472 -2.412516 2.122536
H 1.979699 -0.862183 2.194229
C 3.845641 -3.415355 -0.035315
H 2.665106 -2.646673 -1.654686
C 4.136300 -3.340234 1.327277
H 3.675489 -2.350081 3.187266
H 4.362548 -4.137646 -0.663632
H 4.880064 -4.000429 1.767482
H 0.732827 -1.357678 -1.912681
Sum of electronic and thermal Free Energies= -1636.484131
SCF Energies= -1636.873372
P4_TS1_phenyl33
P 1.507508 -0.809450 0.094189
O -0.026954 -1.059186 0.416344
Si -1.084067 -0.250589 -0.865629
H 0.462201 0.104614 -1.379207
C 2.132168 0.858998 0.449256
C 1.674282 1.506425 1.605390
C 3.070961 1.493326 -0.375416
C 2.152694 2.775815 1.930554
H 0.932778 1.029146 2.240060
C 3.552970 2.757790 -0.039948
H 3.417454 1.010827 -1.284432
C 3.094595 3.400676 1.112133
H 1.784279 3.277284 2.820899
H 4.280611 3.244413 -0.683576
H 3.466294 4.389194 1.367105
C 2.333353 -1.938761 1.347674
C 3.115737 -2.951416 0.478033
C 2.369308 -1.663026 -1.310227
H 1.578188 -2.409144 1.982806
H 3.014176 -1.364127 1.984232
C 3.706531 -2.100421 -0.667502
H 4.294189 -2.699611 -1.373779
H 4.332347 -1.271815 -0.317342
C 2.138218 -3.883893 -0.283862
H 2.659437 -4.783677 -0.627307
H 1.308329 -4.211094 0.350359
C 1.652192 -3.028443 -1.498341
H 1.984933 -3.463900 -2.446615
H 0.564528 -2.937721 -1.551603

H 3.859493 -3.499034 1.065068
H 2.420393 -1.055891 -2.213218
C -1.469859 1.614552 -0.714043
C -0.682838 2.564067 -1.392350
C -2.560243 2.098408 0.032398
C -0.973911 3.927338 -1.342876
H 0.176496 2.224942 -1.966272
C -2.848759 3.463721 0.101597
H -3.197266 1.397870 0.564204
C -2.058421 4.382499 -0.589547
H -0.352212 4.635846 -1.885848
H -3.695573 3.808307 0.690879
H -2.284655 5.445246 -0.541844
C -2.635407 -1.085970 -0.057998
C -2.813407 -1.304617 1.321878
C -3.690053 -1.476116 -0.901403
C -3.982686 -1.872966 1.832001
H -2.018361 -1.033006 2.012095
C -4.865905 -2.044350 -0.404321
H -3.588354 -1.337209 -1.977139
C -5.015766 -2.244437 0.968380
H -4.087942 -2.029898 2.903688
H -5.661616 -2.334459 -1.087252
H -5.926714 -2.689129 1.362394
H -1.318027 -0.819671 -2.241878

Sum of electronic and thermal Free Energies= -1636.482963

SCF Energies= -1636.872531

2.6 Benchmark of Reactants and Transition States for the Phosphine Oxides 4, 7, and S2 Using High-Level DFT Methods Combined With 6-311+G(d,p) Basis Set

A benchmark study was performed to determine which method best reproduced the kinetics profile for the reduction of the phosphine oxides **4**, **7**, and **S2**. As displayed in Figure S5 (vide intra), the initial rates for **4**, **7**, and **S2** were 0.32, 1.42, and 1.12 min^{-1}, respectively, indicating that **4** was 0.81 kcal/mol higher in its barrier than **7**, and 0.70 kcal/mol higher than **S2**. Table S1 reveals that the barrier for **S2** was overestimated when using gas phase B3LYP/6-31G* optimization combined with single-point energy correction under either B3LYP-D3/6-311+G(d,p) or SMD(CH3CN)-M06-2X/6-311+G(d,p) in the implicit solvation model. Therefore, the TS was re-optimized with the SMD(CH3CN)-B3LYP/6-31G* method in implicit solvent, and single-point energy correction under SMD(CH3CN)-B3LYP-D3/6-311+G(d,p). The frequency was also corrected using the quasi-harmonic approximation, as described in Section 2.1. Notably, computation without frequency correction underestimated the barrier for **S2**, as exemplified by the values in parentheses. Consequently, the data calculated using SMD(CH3CN)-B3LYP-D3/6-311+G(d,p)//SMD(CH3CN)-B3LYP/6-31G* are reported in the manuscript.

Table S1. Benchmark of methods for the phosphine oxides **4**, **7**, and **S2** (energies in kcal/mol).

**4**	**7**	**S2**
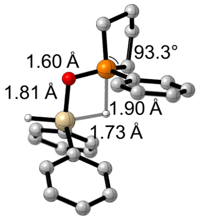	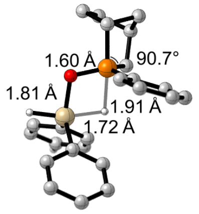	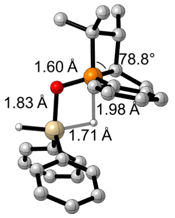
25.9	25.5	27.8
B3LYP-D3/6-311+G(d,p)//B3LYP/6-31G*		
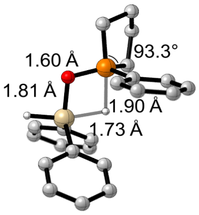	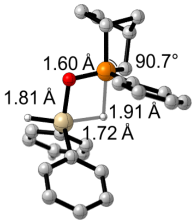	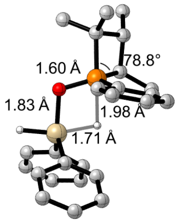
27.6	27.3	28.6
SMD(CH_3CN)-M06-2X/6-311+G(d,p)//B3LYP/6-31G*		
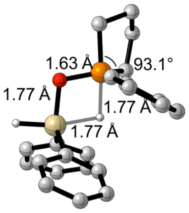	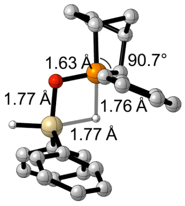	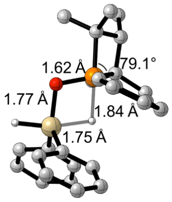
30.1 (27.4)	29.3 (26.9)	30.0 (26.5)

SMD(CH_3CN)-B3LYP-D3/6-311+G(d,p)//SMD(CH3CN)-B3LYP/6-31G*
The values in parentheses represent the free energy barriers before quasi-harmonic correction.

2.7 Distortion–Interaction Analysis of the Activation Barriers for Diphenylsilane-Mediated Reductions of Substrates 4, 7, and S2.

The activation energy for a bimolecular reaction can be decomposed into the distortion energy and the energy of interaction between distorted fragments. The distortion energy is the energy consumed to distort the reactants into the geometries they adopt in the TS without allowing interaction between the fragments. As revealed in Table S2, for substrate **4**, the distortion energy was 61.2 kcal/mol (43.2 kcal/mol for Ph_2SiH_2 and 18.0 kcal/mol for **4**), while the interaction energy was –47.1 kcal/mol. For substrate **7**, the distortion energy was 60.8 kcal/mol (43.5 kcal/mol for the Ph_2SiH_2 and 17.3 kcal/mol **7**), while the interaction energy was –47.6 kcal/mol. Thus, both distortion and interaction contributed to the energy gaps in the activation barriers for substrates **4** and **7**, with distortion of the phosphine oxide being more significant than that of the silane. For

substrate **S2**, the distortion energy was 58.6 kcal/mol (41.5 kcal/mol for Ph_2SiH_2 and 17.1 kcal/mol for **S2**), while the interaction energy was –45.1 kcal/mol.

Table S2. Distortion/interaction analysis for the reduction of phosphine oxides **4**, **7** and **S2** (energies in kcal/mol).

**4**	**7**	**S2**
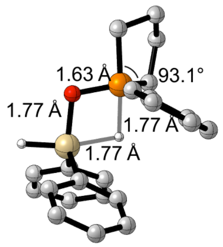	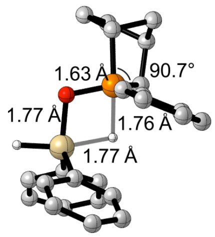	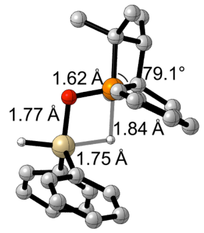
Distortion: 61.2 kcal/mol<br>43.2 kcal/mol for Ph_2SiH_2<br>18.0 kcal/mol for 4<br>Interaction: -47.1 kcal/mol	Distortion: 60.8 kcal/mol<br>43.5 kcal/mol for Ph_2SiH_2<br>17.3 kcal/mol for **7**<br>Interaction: -47.6 kcal/mol	Distortion: 58.6 kcal/mol<br>41.5 kcal/mol for Ph_2SiH_2<br>17.1 kcal/mol for **S2**<br>Interaction: -45.1 kcal/mol

2.8 Geometries and Energies of Phosphine Oxides 7′, S1, and S2.

The geometry optimization was performed at the B3LYP/6-31G(d) level of theory in implicit acetonitrile using the SMD(CH_3CN)-B3LYP/6-31G(d) method. Normal vibrational mode analysis at the same level confirmed that optimized structures were minima or TSs. The frequencies were corrected using quasi-harmonic approximation, as described in Section 2.1. Single-point energy calculations were conducted using SMD(CH_3CN)-B3LYP-D3/6-311+G(d,p) based on the optimized structures. The selection of the method is further elaborated in the benchmark described in Section 2.6.

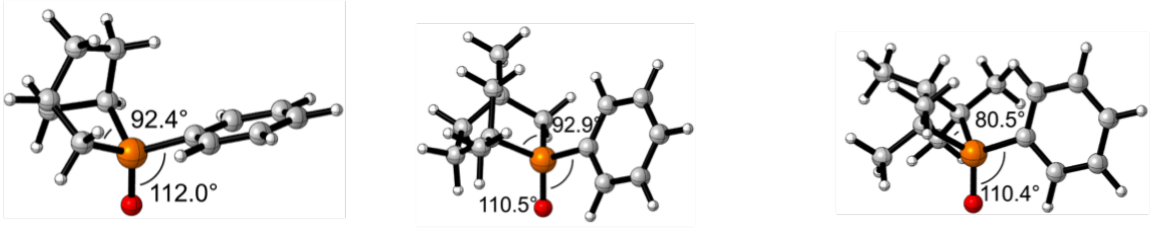

7’　　**S1**　　**S2**

Figure S4. Optimized structures of the phosphine oxides **7′**, **S1**, and **S2**, determined using the SMD(CH_3CN)-B3LYP /6-31G(d) method in the implicit solvent CH_3CN.

Compound **7′**
P 0.523907 0.949447 -0.098772
O 0.529238 2.422596 -0.458887
C -1.172736 0.276146 0.003898
C -1.840948 0.149228 1.231453
C -1.866792 -0.014246 -1.183772
C -3.174011 -0.266194 1.271598
H -1.327837 0.378895 2.160897
C -3.197400 -0.431784 -1.141534
H -1.367282 0.083826 -2.144604
C -3.852885 -0.558538 0.086755
H -3.680538 -0.361082 2.228403
H -3.722451 -0.657990 -2.065760
H -4.889411 -0.883518 0.119074
C 1.489709 0.504268 1.424984
C 2.622479 -0.409591 0.884325
C 1.573055 -0.134403 -1.174241
H 0.892985 0.018066 2.201594
H 1.891733 1.435955 1.839180
C 2.955668 0.135319 -0.524228
H 3.743621 -0.447572 -1.015881
H 3.251250 1.190578 -0.527636
C 2.062988 -1.814412 0.553493
H 2.880884 -2.539024 0.477123
H 1.378719 -2.174600 1.329311
C 1.358775 -1.631196 -0.829122
H 1.846919 -2.241697 -1.596909
H 0.304736 -1.922177 -0.810872
H 3.472010 -0.436697 1.573172
H 1.484744 0.106982 -2.236055
Sum of electronic and thermal Free Energies= -882.720843
SCF Energies= -882.928153
Compound **S1**
P -0.122568 -0.528891 -0.712714
O -0.068661 -1.682174 -1.693750
C 1.571252 0.035006 -0.288310
C 1.885850 1.322470 0.176625
C 2.606521 -0.901872 -0.444716
C 3.206038 1.660654 0.482514
H 1.110016 2.071958 0.297725
C 3.924657 -0.563113 -0.135762
H 2.374608 -1.893062 -0.823052
C 4.226645 0.719255 0.329275
H 3.436258 2.662134 0.836299
H 4.715011 -1.298291 -0.262611
H 5.253183 0.985719 0.566814
C -1.166886 0.930326 -1.176373
C -2.287816 0.956267 -0.096164
C -1.087681 -0.838976 0.858619
H -1.555602 0.796080 -2.191609
H -0.583115 1.857191 -1.161703
C -1.556634 0.590446 1.220842
H -2.249476 0.561081 2.072645
H -0.742516 1.279918 1.471926
C -3.202857 -0.289094 -0.286288
H -4.159690 -0.127149 0.222616
H -3.425561 -0.466910 -1.344433
C -2.424679 -1.468522 0.371420
H -2.964579 -1.850947 1.246361
H -2.263613 -2.310890 -0.307858
C -3.045954 2.279758 -0.082255
H -3.563888 2.451027 -1.034648
H -3.801009 2.288831 0.713993
H -2.368448 3.126423 0.084790
C -0.362149 -1.634638 1.936226
H -0.078782 -2.631950 1.577277
H 0.549656 -1.128058 2.272120
H -1.012481 -1.770132 2.810138
Sum of electronic and thermal Free Energies= -961.301605
SCF Energies= -961.561557
Compound **S2**
P -0.231952 0.002606 0.827520
O -0.241678 0.008132 2.339858
C 1.492251 -0.000251 0.198933
C 2.507954 0.005654 1.167746
C 1.848580 -0.007257 -1.160629
C 3.851701 0.004779 0.788287
H 2.235923 0.010959 2.219002
C 3.192226 -0.008172 -1.537991
H 1.086163 -0.012102 -1.932068
C 4.196173 -0.002068 -0.565240
H 4.627416 0.009443 1.549612
H 3.454005 -0.013592 -2.592683
H 5.241644 -0.002713 -0.862120
C -1.313660 -1.210696 -0.102949
C -1.819376 -0.003091 -0.986419
H -1.222093 -0.006730 -1.907251
C -1.312219 1.210331 -0.111808
C -2.370016 1.812322 0.828980
H -1.903089 2.531332 1.511475

H -3.125658 2.348955 0.242314
H -2.888010 1.067847 1.438710
C -0.652173 2.339498 -0.905211
H -1.411122 2.865313 -1.499894
H -0.188710 3.073937 -0.235052
H 0.116943 1.981490 -1.594386
C -3.291632 -0.003604 -1.389125
H -3.524385 0.879314 -1.996735
H -3.525515 -0.890401 -1.990624
H -3.960747 -0.000191 -0.523343
C -2.371980 -1.804417 0.842555
H -3.129023 -2.343534 0.259994
H -1.905895 -2.519791 1.529435
H -2.888102 -1.055014 1.447859
C -0.655162 -2.346605 -0.887964
H -0.192340 -3.076468 -0.212398
H -1.414869 -2.876023 -1.478465
H 0.114160 -1.994700 -1.580042

Sum of electronic and thermal Free Energies= -962.512983

SCF Energies= -962.791122

2.9 Transition State Geometries and Energies for the Phosphine Oxides 4, 7, 7′, S1, and S2

Based on the detailed computational exploration of the various TS geometries for **4** and **7**, the TS structures with the hydride occupying the axial position were demonstrated to be more favorable, consistent with the results reported by Krenske.[1] Consequently, in the following calculations of the phosphine oxides **4**, **7**, **7′**, **S1**, and **S2** in acetonitrile (Table S3), this configuration was adopted for direct calculations of energetics, without any redundant geometrical testing.

Table S3. Transition structures and activation free energies for the reduction of phosphine oxides **4**, **7**, **7′**, **S1**, and **S2** (energies in kcal/mol).

**4**	**7**	**7’**	**S1**	**S2**
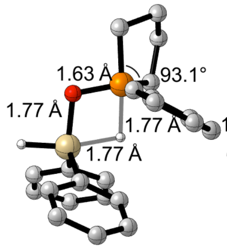	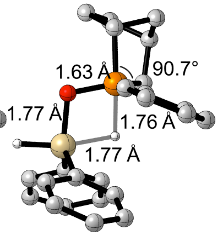	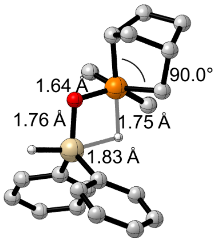	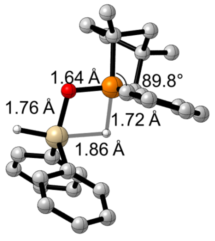	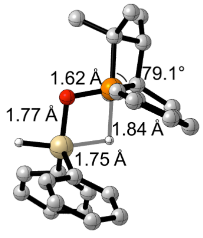
30.1	29.3	29.5	30.4	30.0

Compound **4**_TS
15 0.379298 -1.633311 -0.464546
6 0.998468 -3.097645 -1.445742
8 -0.305558 -0.805407 -1.688114
14 -1.039072 0.628041 -0.962340
6 -0.566719 -2.697979 0.720671
1 -1.486977 1.144788 -2.316768
1 -0.200764 -2.468602 1.726899
1 -1.616927 -2.395840 0.684894
1 0.276112 -3.306080 -2.244156
1 1.970737 -2.905378 -1.907624
1 -0.374598 -0.286636 0.394437
6 1.013461 -4.246532 -0.427579
1 1.149147 -5.219794 -0.911814
6 -0.328449 -4.169048 0.325531
1 -0.341971 -4.816966 1.208159
1 1.845103 -4.113733 0.278240
1 -1.133091 -4.502185 -0.341148
6 0.105062 2.054420 -0.455811
6 0.487013 3.012478 -1.412941
6 0.609671 2.202041 0.849962
6 1.347799 4.065505 -1.087197
1 0.113029 2.934075 -2.431836
6 1.460592 3.255266 1.187165
1 0.344920 1.471681 1.610103
6 1.836965 4.188296 0.215203
1 1.636490 4.786581 -1.848210
1 1.838793 3.343966 2.202780
1 2.507944 5.004180 0.472490
6 -2.734926 0.498562 -0.117765
6 -2.898579 0.158052 1.238996
6 -3.896373 0.789973 -0.858412
6 -4.161754 0.114659 1.832219
1 -2.023409 -0.079170 1.839388
6 -5.165923 0.741312 -0.274681
1 -3.810718 1.059298 -1.909113
6 -5.300747 0.404092 1.074132
1 -4.259588 -0.147537 2.883019
1 -6.046960 0.965930 -0.871118
1 -6.285953 0.365376 1.532227
6 1.943590 -0.933687 0.170074
6 2.217101 -0.833756 1.540291
6 2.860174 -0.423629 -0.759898
6 3.403605 -0.241574 1.973801
1 1.502946 -1.199153 2.272070
6 4.036484 0.188341 -0.321288
1 2.651970 -0.487770 -1.824682
6 4.311696 0.276299 1.045057
1 3.613357 -0.173687 3.037820
1 4.734910 0.594825 -1.047578
1 5.228241 0.749498 1.386666

Compound **7**_TS
P -0.029974 -1.428703 -0.209084
O -0.460675 -0.542318 -1.502874
Si -0.715941 1.100473 -0.906169
H -0.323025 0.146428 0.528687
C 1.695306 -1.181789 0.332539
C 2.071677 -1.177819 1.681583
C 2.652533 -0.928052 -0.659458
C 3.399087 -0.927520 2.032742
H 1.335365 -1.354543 2.460050
C 3.975785 -0.661478 -0.301964
H 2.363989 -0.918257 -1.706922
C 4.351138 -0.662758 1.043406
H 3.687197 -0.929335 3.080465
H 4.709692 -0.450555 -1.074875
H 5.380901 -0.455225 1.321244
C -1.239214 -2.072513 1.042781
C -1.405203 -3.572458 0.713462
C 0.076962 -3.121200 -1.011685
H -2.165038 -1.495130 0.959424
H -0.829350 -1.898483 2.043274
C -0.008361 -4.030657 0.234216
H 0.005060 -5.091980 -0.041019
H 0.782368 -3.856232 0.973690
C -2.254976 -3.746077 -0.568502
H -2.632736 -4.771993 -0.632582
H -3.121570 -3.076047 -0.577307
C -1.254599 -3.449960 -1.732723
H -1.115598 -4.340419 -2.356508
H -1.588179 -2.640789 -2.387316
H -1.798887 -4.123189 1.572636
H 0.968213 -3.241390 -1.632022
C 0.838212 2.128305 -0.552222
C 1.487134 2.785675 -1.613444
C 1.410176 2.238928 0.729357
C 2.667983 3.507033 -1.411069
H 1.072259 2.726521 -2.617698
C 2.581405 2.966484 0.943232
H 0.943325 1.729616 1.568807
C 3.218223 3.597724 -0.130792
H 3.157146 3.994998 -2.250785

H 3.005353 3.031972 1.942366
H 4.137848 4.154755 0.030756
C -2.341294 1.567846 -0.042157
C -2.570444 1.377238 1.334277
C -3.368670 2.169882 -0.793796
C -3.766604 1.773215 1.935820
H -1.801457 0.907805 1.943246
C -4.572247 2.563378 -0.201143
H -3.229189 2.334286 -1.860185
C -4.772980 2.366547 1.167114
H -3.916389 1.617092 3.001476
H -5.351039 3.021728 -0.805944
H -5.707472 2.670873 1.631970
H -1.036789 1.610114 -2.298738

Sum of electronic and thermal Free Energies= -1636.584215

SCF Energies= -1636.976685

Compound **7′**_TS

P -1.037955 -0.928491 -0.333399
O -0.023741 -0.618079 -1.580678
Si 1.394164 0.196999 -0.935360
H 0.292542 -0.138152 0.485515
C -0.740676 -2.349484 0.834964
C -1.607075 -3.513076 0.311696
C -2.250038 -1.993332 -1.319015
H -0.966343 -2.040901 1.861011
H 0.327455 -2.585011 0.786928
C -1.559607 -3.374561 -1.227778
H -2.148459 -4.153988 -1.727007
H -0.542743 -3.394104 -1.635689
C -3.101957 -3.253715 0.612491
H -3.671731 -4.186566 0.542977
H -3.250771 -2.855037 1.622092
C -3.540765 -2.249365 -0.500168
H -4.291125 -2.705869 -1.156058
H -3.985443 -1.336498 -0.094678
H -1.246591 -4.472165 0.696557
H -2.428805 -1.618041 -2.328901
C 2.747572 -0.809080 -0.055278
C 3.813068 -1.322382 -0.820365
C 2.784918 -1.029545 1.335451
C 4.864189 -2.029345 -0.228750
H 3.824686 -1.167537 -1.897109
C 3.836811 -1.724128 1.935969
H 1.976529 -0.654139 1.958651
C 4.879247 -2.229612 1.152991
H 5.669884 -2.420199 -0.845628
H 3.842839 -1.874826 3.012869
H 5.696246 -2.775789 1.617939
C 1.301471 2.046359 -0.508723
C 1.048301 2.538172 0.786145
C 1.547937 2.990755 -1.524071
C 1.045750 3.907431 1.057839
H 0.848078 1.839670 1.594813
C 1.531989 4.364318 -1.264001
H 1.756578 2.649836 -2.536030
C 1.283002 4.825619 0.030158
H 0.853679 4.259652 2.068561
H 1.716973 5.071299 -2.069211
H 1.273603 5.892701 0.238332
H 2.026950 0.298152 -2.306263
C -2.066196 0.473291 0.238937
C -2.742804 1.248861 -0.713539
C -2.183857 0.788652 1.599912
C -3.526247 2.331594 -0.306217
H -2.658888 1.014682 -1.771382
C -2.984744 1.857400 2.003598
H -1.647562 0.206086 2.344076
C -3.653465 2.632762 1.051341
H -4.039306 2.934414 -1.050619
H -3.079596 2.089885 3.060878
H -4.268661 3.470925 1.367302

Sum of electronic and thermal Free Energies= -1636.521382

SCF Energies= -1636.911645

Compound **S1**_TS

P -1.098884 -0.345047 -0.231496
O -0.238950 0.214995 -1.509426
Si 1.267490 0.887539 -0.910596
H 0.320597 0.227189 0.550387
C -0.600713 -2.028309 0.302298
C -0.840521 -2.511768 1.597472
C 0.067207 -2.848238 -0.621099
C -0.427256 -3.796378 1.956816
H -1.345475 -1.895472 2.334854
C 0.482451 -4.130754 -0.255345
H 0.268865 -2.488471 -1.625644
C 0.234744 -4.608592 1.032930
H -0.622495 -4.159226 2.962423
H 0.999987 -4.753566 -0.980026
H 0.557068 -5.606811 1.316564
C -2.024069 0.651725 1.041583

C -3.525827 0.591758 0.669046
C -2.735334 -0.654588 -1.167697
H -1.624499 1.670328 1.053857
H -1.814422 0.212623 2.024064
C -3.711504 -0.802375 0.022133
H -4.742299 -0.946568 -0.329522
H -3.468359 -1.630717 0.699072
C -3.758596 1.517576 -0.552748
H -4.831574 1.705483 -0.675290
H -3.271150 2.490721 -0.423573
C -3.199669 0.712155 -1.760785
H -3.993683 0.518957 -2.492930
H -2.396798 1.230558 -2.291900
C 2.707487 -0.241066 -0.398373
C 3.712380 -0.517852 -1.346109
C 2.871345 -0.775799 0.894089
C 4.827946 -1.295752 -1.022388
H 3.624333 -0.120469 -2.354893
C 3.986391 -1.546276 1.227950
H 2.111169 -0.591949 1.649280
C 4.968054 -1.810509 0.267850
H 5.585371 -1.497734 -1.775912
H 4.088924 -1.944110 2.234710
H 5.834639 -2.414850 0.524372
C 1.217956 2.603280 -0.097212
C 1.094778 2.813528 1.290197
C 1.362531 3.741193 -0.914700
C 1.116908 4.097830 1.837454
H 0.977777 1.959673 1.953422
C 1.373578 5.031214 -0.375811
H 1.468952 3.620272 -1.990608
C 1.252386 5.211926 1.003357
H 1.025630 4.231006 2.912724
H 1.479219 5.891946 -1.031715
H 1.263700 6.213062 1.426932
H 1.733304 1.271404 -2.294484
C -2.746507 -1.782922 -2.193203
H -2.053977 -1.582631 -3.019581
H -2.474667 -2.748310 -1.753514
H -3.751407 -1.887615 -2.625215
C -4.425683 0.898468 1.862975
H -4.252778 1.914528 2.240522
H -5.484035 0.822669 1.582950
H -4.244695 0.201440 2.690791
Sum of electronic and thermal Free Energies= -1715.099728
SCF Energies= -1715.541832
Sum of electronic and thermal Free Energies= -1559.200682
SCF Energies= -1559.557477

Compound **S2**_TS
P 0.513521 -1.201469 -0.215475
O -0.170778 -0.514659 -1.515700
Si -1.020661 0.936246 -0.949020
H -0.469630 0.169606 0.522757
C 1.982871 -0.258480 0.318661
C 2.327500 -0.018752 1.654738
C 2.762842 0.295012 -0.709074
C 3.448829 0.755655 1.957047
H 1.717577 -0.407437 2.460990
C 3.873909 1.081052 -0.401023
H 2.492034 0.129637 -1.747951
C 4.220482 1.310504 0.932618
H 3.709181 0.938125 2.996065
H 4.462791 1.517354 -1.203030
H 5.084305 1.924213 1.173054
C -0.370394 -2.418801 0.917758
C 1.167928 -2.856018 -0.883920
C 0.581023 -3.553309 0.398567
C 0.015551 2.464006 -0.505148
C 0.416339 3.350999 -1.521104
C 0.419122 2.757937 0.810743
C 1.199609 4.475176 -1.241234
H 0.120701 3.158307 -2.550473
C 1.190952 3.883520 1.101964
H 0.141361 2.083293 1.616795
C 1.589338 4.743273 0.072802
H 1.506109 5.138293 -2.046841
H 1.492528 4.085257 2.127025
H 2.200500 5.614626 0.295029
C -2.789829 0.882156 -0.258537
C -3.069575 0.915332 1.121342
C -3.882968 0.852001 -1.142976
C -4.381244 0.922704 1.598349
H -2.247233 0.927344 1.833718
C -5.200688 0.838689 -0.673976
H -3.705821 0.836107 -2.216505
C -5.452377 0.877383 0.699011
H -4.570885 0.956571 2.668589
H -6.027659 0.801595 -1.379202
H -6.475095 0.871731 1.067794
H -1.367359 1.299701 -2.385568

H 1.399904 -3.635776 1.124754
C -0.029250 -4.941397 0.224320
H 0.729063 -5.649213 -0.131251
H -0.413564 -5.318612 1.179608
H -0.853192 -4.943680 -0.495198
C -0.326934 -2.166814 2.423264
H 0.693523 -2.190666 2.816239
H -0.781092 -1.201912 2.677097
H -0.899233 -2.949554 2.937762
C -1.836473 -2.552024 0.472166
H -2.413830 -1.685411 0.803815
H -1.964437 -2.648281 -0.609024
H -2.268307 -3.444097 0.940321
C 0.515391 -3.274361 -2.209939
H 0.889852 -2.647429 -3.026068
H 0.771017 -4.316694 -2.440304
H -0.574223 -3.191284 -2.205772
C 2.686120 -3.023971 -0.999411
H 2.925009 -4.082051 -1.173702
H 3.092470 -2.447619 -1.838717
H 3.207849 -2.709024 -0.089845

Sum of electronic and thermal Free Energies= -1716.348882

SCF Energies= -1716.81075

2.10 Geometries and Energies of Phosphine Oxides 5, 6, 7a, Ph_3PO, and Bu_3PO

The geometry optimization was performed at the B3LYP/6-31G(d) level of theory in implicit acetonitrile using the SMD(CH_3CN)-B3LYP/6-31G(d) method. Normal vibrational mode analysis at the same level confirmed that optimized structures were minima or TSs. The frequencies were corrected using quasi-harmonic approximation, as described in Section 2.1. Single-point energy calculations were conducted using SMD(CH_3CN)-B3LYP-D3/6-311+G(d,p) based on the optimized structures. The selection of the method is further elaborated in the benchmark described in Section 2.6. Please note that the optimization convergence criteria were set to a maximum step size of 0.0018 au and an RMS force of 0.0003 au, with the exception of **7a** (a maximum step size of 0.01 au and an RMS force of 0.0017 au).

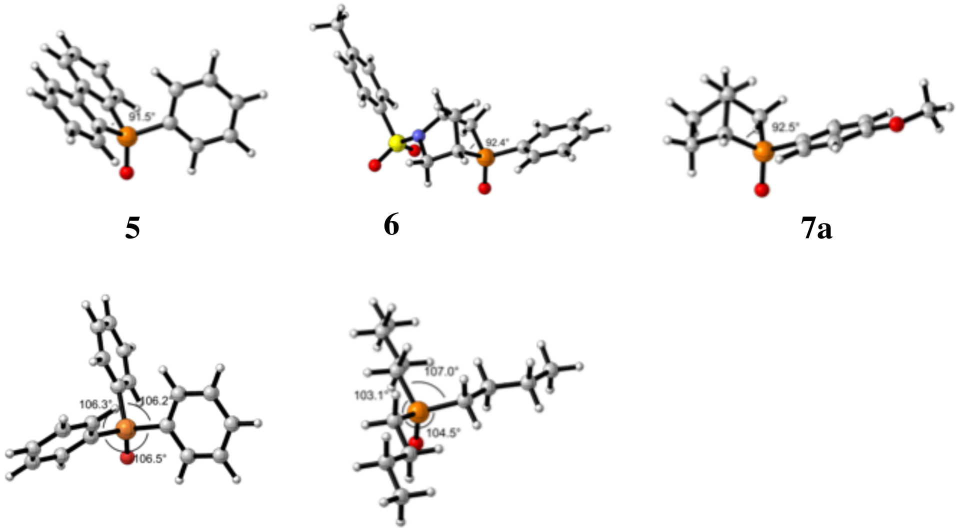

5 **6** **7a**

Ph_3PO Bu_3PO

Figure S5. Optimized structures of the phosphine oxides **5**, **6**, **7a**, Ph_3PO, and Bu_3PO, determined using the SMD(CH3CN)-B3LYP /6-31G(d) method in the implicit solvent CH_3CN.

Compound 5

C 0.569338 -2.686155 0.540522
C 0.732957 -1.306006 0.456233
C 1.851886 -0.741327 -0.193725
C 2.811199 -1.582211 -0.763170
C 2.647541 -2.968494 -0.674683
C 1.537246 -3.521865 -0.028448
H -0.298621 -3.110313 1.038872
H 3.677291 -1.168646 -1.271793
H 3.394284 -3.622853 -1.116236
H 1.424853 -4.600728 0.030203
C 1.851716 0.741637 -0.193599
C 2.810796 1.582836 -0.762968
C 0.732680 1.305952 0.456493
C 2.646813 2.969067 -0.674272
H 3.676950 1.169558 -1.271716
C 0.568735 2.686049 0.540984
C 1.536418 3.522077 -0.027902
H 3.393375 3.623666 -1.115773
H -0.299307 3.109922 1.039431
H 1.423763 4.600904 0.030916
P -0.354206 -0.000202 1.115591
O -0.640711 -0.000420 2.597416
C -1.876270 -0.000177 0.114556
C -3.111603 -0.000646 0.773809
C -1.831939 0.000353 -1.288604
C -4.297637 -0.000575 0.034957
H -3.137458 -0.001040 1.859645
C -3.017515 0.000413 -2.022304
H -0.874993 0.000722 -1.804903
C -4.250959 -0.000044 -1.360844
H -5.254784 -0.000927 0.549454
H -2.981384 0.000823 -3.108361
H -5.173595 0.000016 -1.935126

Sum of electronic and thermal Free Energies= -1110.202409

SCF Energies= -1110.415874

Compound 6

C 0.333596 -1.762918 1.231361
C 1.560894 -0.840950 1.365070
C -0.028049 0.481886 0.346841
H 0.529699 -2.641797 0.611485
H -0.031214 -2.094335 2.207975
C 0.906383 0.543904 1.568407
H 1.610806 1.379088 1.515193
H 0.349544 0.599854 2.509048
C 0.898806 0.452479 -0.892812
H 1.247063 1.462516 -1.125506
H 0.409361 0.032897 -1.773415
H 2.264549 -1.166238 2.131776
H -0.789618 1.261632 0.294655
P 2.283458 -0.661163 -0.339256
C 3.791427 0.349342 -0.159579
C 4.700256 0.074978 0.875845
C 4.099810 1.348348 -1.096882
C 5.890716 0.796513 0.977342
H 4.484568 -0.700781 1.605116
C 5.292848 2.065685 -0.994584
H 3.413990 1.569342 -1.910185
C 6.187596 1.793117 0.043734
H 6.584565 0.581147 1.785353
H 5.521772 2.838561 -1.723231
H 7.113914 2.355452 0.125035
O 2.488081 -1.934921 -1.127867
N -0.667135 -0.836875 0.611775
S -1.849458 -1.461811 -0.375985
O -1.476048 -1.480988 -1.805621
O -2.237818 -2.740952 0.245087
C -3.145181 -0.246350 -0.174925
C -3.605565 0.055656 1.111957
C -3.702146 0.358059 -1.301101
C -4.631338 0.982604 1.260520
H -3.156815 -0.415619 1.980677
C -4.733464 1.283395 -1.132158
H -3.329077 0.115748 -2.290305
C -5.212059 1.609268 0.143398
H -4.988103 1.229751 2.257197
H -5.167123 1.762191 -2.006033
C -6.331871 2.601917 0.326149
H -6.523648 3.165345 -0.592209

H -7.262023 2.090493 0.606789
H -6.102478 3.312609 1.128471

Sum of electronic and thermal Free Energies= -1717.663331
SCF Energies= -1717.963695

Compound 7a

P 1.262376 -0.098856 0.786576
O 1.565037 -0.532408 2.206895
C -0.509887 -0.139237 0.370615
C -1.327331 0.986555 0.540711
C -1.110027 -1.339991 -0.055813
C -2.698688 0.937419 0.284488
H -0.901055 1.927217 0.879357
C -2.471918 -1.403817 -0.316045
H -0.510086 -2.236128 -0.189326
C -3.277556 -0.263658 -0.151564
H -3.298445 1.829622 0.421688
H -2.935420 -2.326387 -0.652876
C 1.943416 1.551175 0.259908
C 2.882085 1.189434 -0.920503
C 2.150883 -0.990411 -0.571055
H 2.471831 2.016590 1.098160
H 1.147828 2.228319 -0.064134
C 2.140257 0.072897 -1.693109
H 2.705252 -0.270310 -2.568151
H 1.135870 0.363843 -2.021859
C 4.137728 0.438688 -0.408231
H 4.925671 0.465107 -1.168399
H 4.543279 0.895308 0.501086
C 3.648143 -1.028225 -0.173249
H 4.179182 -1.727898 -0.827693
H 3.795337 -1.364220 0.857523
H 3.127577 2.070913 -1.520102
H 1.724094 -1.963511 -0.821430
O -4.593738 -0.426264 -0.439718
C -5.460182 0.705615 -0.324741
H -5.149950 1.515503 -0.996396
H -6.450354 0.351639 -0.618192
H -5.498182 1.077944 0.706211
Sum of electronic and thermal Free Energies= -997.250928
SCF Energies= -997.487417

Ph_3PO

C -0.89162600 2.61269400 1.01875800
C -0.21308300 1.67083400 0.23047900
C 0.25173100 2.03947800 -1.04126000
C 0.02259700 3.32966200 -1.52379300
C -0.66078200 4.26121400 -0.73755300
C -1.11380500 3.90307000 0.53546100
H -1.23491600 2.33492900 2.01115000
H 0.38278900 3.60755500 -2.51060900
H -0.83448100 5.26586300 -1.11373600
H -1.63699300 4.62860500 1.15250900
C -1.90079700 -0.79442300 -1.03399300
C -2.91350800 -1.62823700 -1.51220500
C -1.34102300 -1.02417000 0.23171700
C -3.37607000 -2.68978100 -0.72943000
H -3.34380000 -1.44569900 -2.49315800
C -1.81385900 -2.08675600 1.01699900
C -2.82765300 -2.91673400 0.53616900
H -4.16668000 -3.33513400 -1.10308100
H -1.39321600 -2.25210600 2.00477900
H -3.19204000 -3.73614700 1.14985200
P 0.00325400 -0.00380000 0.92835700
O 0.00189300 -0.00341800 2.43836600
C 1.56051200 -0.65353700 0.23105100
C 2.72097200 -0.50718200 1.00612800
C 1.64109600 -1.26757400 -1.02808800
C 3.94933800 -0.95995800 0.52345000
H 2.65547100 -0.04935500 1.98916900
C 2.87255800 -1.71586600 -1.50939100
H 0.74856100 -1.40291100 -1.63192300
C 4.02637200 -1.56231200 -0.73556000
H 4.84359800 -0.84757100 1.13062600
H 2.92920500 -2.19161500 -2.48466400
H 4.98256200 -1.91726100 -1.11049500
H -1.55758900 0.03694100 -1.64323500
H 0.79575500 1.32572200 -1.65309500
Free energy is not calculated because the structure is only loosely converged.
SCF Energies= -1111.6113

Bu_3PO

P -0.331082 -0.401616 0.326956
O -0.265939 -0.526237 1.837529

C 0.932376 -1.411610 -0.552327
H 0.512022 -2.422094 -0.647760
H 1.037170 -1.015837 -1.571058
C 2.296034 -1.486949 0.150164
H 2.728351 -0.483145 0.245373
H 2.156587 -1.860358 1.172901
C 3.284941 -2.390398 -0.596897
H 3.404539 -2.022205 -1.625892
H 2.861267 -3.401528 -0.678245
C 4.653767 -2.457970 0.085579
H 5.345113 -3.107354 -0.465136
H 5.111296 -1.462379 0.151821
H 4.567826 -2.850992 1.106836
C -1.911436 -0.985549 -0.400926
H -1.990046 -2.050443 -0.142559
H -1.821537 -0.929150 -1.494132
C -3.157584 -0.230926 0.083403
H -3.091740 0.825464 -0.209980
H -3.192469 -0.248599 1.181193
C -4.456175 -0.826804 -0.474832
H -4.418407 -0.810300 -1.573413
H -4.520789 -1.884884 -0.184288
C -5.704133 -0.082999 0.008630
H -5.678975 0.971239 -0.296153
H -6.619499 -0.527251 -0.400919
H -5.780471 -0.109518 1.103252
C -0.156175 1.324615 -0.280016
H -0.337203 1.321915 -1.363155
H -0.976503 1.892199 0.178922
C 1.186819 1.994989 0.044060
H 1.995888 1.477695 -0.487549
H 1.400709 1.893140 1.116643
C 1.208367 3.480536 -0.337650
H 0.994129 3.581173 -1.411222
H 0.396073 4.000462 0.189774
C 2.545294 4.153081 -0.014380
H 3.371745 3.668109 -0.549770
H 2.539279 5.212580 -0.298005
H 2.766821 4.096095 1.059105

Sum of electronic and thermal Free Energies= -889.899067
SCF Energies= -890.226938

3. Synthesis and Reduction of the Phosphine Oxides 7 and 7′

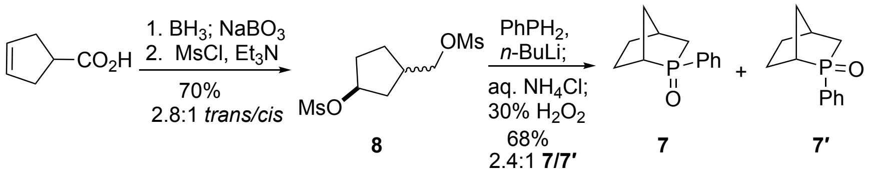

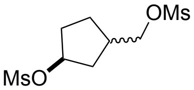

3-([(Methylsulfonyl)oxy]cyclopentyl)methyl Methanesulfonate (8). 1 M Borane in THF (0.6 mL, 0.6 mmol, 0.3 equiv) was added slowly to a solution of cyclopent-3-ene-1-carboxylic acid (224 mg, 2.0 mmol) in THF (20 mL) at –40 °C (dry ice/CH_3CN) under an Ar atmosphere. The mixture was stirred for 1 h at –40 °C and then it was warmed to –20 °C. An additional charge of 1 M BH_3 in THF (3.4 mL, 1.7 equiv) was added dropwise and then the mixture was slowly warmed to room temperature and stirred overnight. Water and $NaBO_3 \cdot 4H_2O$ (1.23 g, 4.0 equiv) were added sequentially. After stirring for 2 h at r.t., dry Na_2SO_4 (1.0 g) was added to the mixture, which was then stirred for another 20 min. The mixture was filtered and concentrated to obtain the diol, which was used in the next step without purification.

A solution of methanesulfonyl chloride (0.39 mL, 5.0 mmol, 2.5 equiv) in DCM (20 mL) was added dropwise to a solution of the diol and dry Et_3N (0.69 mL, 5.0 mmol, 2.5 equiv) in DCM at 0 °C. The mixture was stirred at 0 °C for 30 min and then at room temperature after completion (monitored by TLC). The reaction was quenched through the addition of 1 M HCl, saturated $NaHCO_3$, and saturated brine. The organic phase was dried (Na_2SO_4) and concentrated

(rotary evaporation). The residue was purified through FCC on silica gel to provide **8** in 70% yield over two steps (*trans:cis* = 2.8:1; inseparable mixture). The spectral data for pure **8-*trans*** and **8-*cis*** are those of samples obtained in one of the alternative routes to **7**/**7′** (route 1).

8-*trans*: R_f = 0.5 (1:1 hexanes/EtOAc); IR (ATR) ν_{max} 3030, 2973, 2942, 1342, 1332 cm^{-1}; ^{1}H NMR (500 MHz, $CDCl_3$): δ 5.14–5.11 (m, 1H), 4.14–4.07 (m, 2H), 2.97 (s, 3H), 2.95 (s, 3H), 2.62–2.53 (m, 1H), 2.17–2.12 (m, 1H), 2.06–1.94 (m, 3H), 1.69–1.63 (m, 1H), 1.48–1.39 (m, 1H); ^{13}C NMR (125 MHz, $CDCl_3$): δ 84.1, 72.4, 38.4, 37.3, 36.6, 36.5, 32.7, 26.0; HRMS (ESI) *m/z* $[M + Na]^+$ calcd for $C_8H_{16}O_6S_2Na$ 295.0286, found 295.0284.

8-*cis*: R_f = 0.5 (1:1 hexanes/EtOAc); IR (ATR) ν_{max} 2968, 2952, 2887, 1249, 1180 cm^{-1}; ^{1}H NMR (500 MHz, $CDCl_3$): δ 5.15–5.12 (m, 1H), 4.19–4.12 (m, 2H), 3.00 (s, 3H), 2.98 (s, 3H), 2.46–2.40 (m, 1H), 2.25–2.19 (m, 1H), 2.08–2.03 (m, 1H), 1.94 –1.88 (m, 2H), 1.84–1.79 (m, 1H), 1.62–1.56 (m, 1H); ^{13}C NMR (125 MHz, $CDCl_3$): δ 83.6, 72.7, 38.5, 37.4, 37.0, 36.0, 33.2, 26.3; HRMS (ESI) *m/z* $[M + Na]^+$ calcd for $C_8H_{16}O_6S_2Na$ 295.0286, found 295.0292.

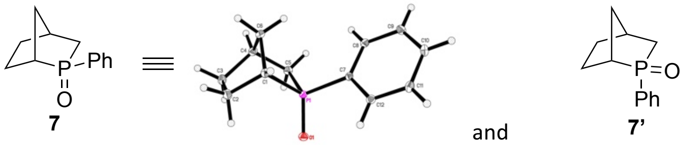

exo*-2-Phenyl-2-phosphabicyclo[2.2.1]heptane-2-oxide(7) and *endo*-2-Phenyl-2-phosphabicyclo[2.2.1]heptane-2-oxide (7′).** *n*-BuLi (12.7 mL, 2.50 M sol. in hexanes, 31.7 mmol, 3.3 equiv) was added to a solution of phenylphosphine (1.59 mL, 14.4 mmol, 1.5 equiv) in THF via syringe at –78 °C.[2] The resulting orange solution was warmed to r.t., stirred for 1 h at r.t., then cooled to –20 °C. A solution of the dimesylate **8** (2.62 g, 9.60 mmol) in THF (20 mL) was added dropwise to the resulting bright-yellow suspension. After the mixture had stirred for 48 h at r.t., the pale-yellow suspension was hydrolyzed with saturated aqueous NH_4Cl. The mixture was extracted with Et_2O; the combined organic phases were dried (Na_2SO_4) and filtered. The solvents were evaporated under reduced pressure. The residue, a white gum, was dissolved in DCM (10 mL) and treated with 15% H_2O_2 (5 mL). After 30 min, the mixture was extracted three times with DCM. The combined organic phases were dried (Na_2SO_4) and concentrated (rotary evaporation). The residue was subjected to CombiFlash® purification on a silica gel column (gradient solvent system: EtOAc/MeOH, from 100:0 to 100:3.3) to afford the products **7** and **7′** as a solid (**7**/**7′** ratio, 2.4:1; 1.42 g, 72%). The phosphine oxides **7** and **7′** were separated by using CombiFlash®R_f^+Lumen UV-VIS/ELSD (Teledyne ISCO) and a RediSep column [silica 12 g Gold (60-Å pore size, 20–40 μm)]. When run on a larger scale (>20 g of the dimesylate **8**), the monoalkylation product (arising from **8-*cis) was separated from **7** and **7′** through silica gel FCC (solvent gradient: 3:1 hexanes:EtOAc, 2:1 hexanes:EtOAc, 1:1 hexanes:EtOAc, pure EtOAc, 20:1 CH_2Cl_2:MeOH, 10:1 CH_2Cl_2:MeOH, then 5:1 CH_2Cl_2:MeOH). The earlier fractions contained a 10:1 mixture of the phosphine oxides **7** and **7′**; this mixture provided pure **7** upon recrystallization using a minimal amount of CH_2Cl_2 (10–15 mL for 10 g of sample) to dissolve the sample, adding 3:1 hexanes:EtOAc (20–30 mL), and leaving the solution open to the air until crystals formed (ca. 3–4 days).

7: M.p. 138 °C; R_f = 0.61 (1:10 MeOH/DCM); IR (ATR) ν_{max} 3030, 2973, 2942, 1342, 1332, 925, 889 cm^{-1}; ^{1}H NMR (500 MHz, $CDCl_3$) δ 7.76–7.71 (m, 2H), 7.50–7.41 (m, 3H), 2.72–2.64 (m, 1H), 2.54–2.41 (m, 1H), 2.50–2.41 (m, 1H), 2.19–2.13 (m, 1H), 1.89–1.77 (m, 2H), 1.70–1.64 (m, 2H), 1.61–1.51 (m, 2H); ^{13}C NMR (125 MHz, $CDCl_3$) δ 133.7 (d, J_{CP} = 88.6 Hz), 131.6 (d, J_{CP} = 2.8 Hz), 130.4 (d, J_{CP} = 9.0 Hz), 128.7 (d, J_{CP} = 11.0 Hz), 37.7 (d, J_{CP} = 66.9 Hz), 36.9 (d,

J_{CP} = 2.1 Hz), 36.7 (d, J_{CP} = 11.9 Hz), 32.8 (d, J_{CP} = 61.7 Hz), 29.3 (d, J_{CP} = 2.0 Hz), 20.8 (d, J_{CP} = 18.6 Hz); ^{31}P NMR (121 MHz, $CDCl_3$) δ 55.8; HRMS (ESI) *m*/*z* $[M + H]^+$ calcd for $C_{12}H_{16}OP$ 207.0939, found 207.0935.

7′: M.p. 54 °C; R_f = 0.60 (1:10 MeOH/DCM); IR (ATR) ν_{max} 3030, 2960, 2880, 1349, 1172, 750 cm^{-1}; ^{1}H NMR (500 MHz, $CDCl_3$) δ 7.76–7.72 (m, 2H), 7.55–7.51 (m, 1H), 7.50–7.46 (m, 2H), 2.86–2.76 (m, 1H), 2.49 (s, 1H), 2.40–2.36 (m, 1H), 2.06–1.97 (m, 2H), 1.86–1.75 (m, 1H), 1.73–1.58 (m, 2H), 1.27–1.12 (m, 2H); ^{13}C NMR (125 MHz, $CDCl_3$) δ 131.8 (d, J_{CP} = 2.8 Hz), 131.1 (d, J_{CP} = 8.9 Hz), 130.2 (d, J_{CP} = 90.0 Hz), 128.6 (d, J_{CP} = 11.1 Hz), 38.1 (d, J_{CP} = 67.3 Hz), 37.4 (d, J_{CP} = 6.1 Hz), 35.6, 35.2 (d, J_{CP} = 59.2 Hz), 28.4, 21.9 (d, J_{CP} = 3.7 Hz); ^{31}P NMR (121 MHz, $CDCl_3$) δ 57.4; HRMS (ESI) *m*/*z* $[M + H]^+$ calcd for $C_{12}H_{16}OP$ 207.0939, found 207.0939.

Reduction of the Phosphine Oxides 7 and 7′ to Phosphines S3 and S4

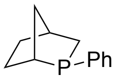

S3

2-Phenyl-2-phosphabicyclo[2.2.1]heptane (S3). Prepared according to the reported method, in 95% yield.[3] R_f = 0.82 (3:1 hexanes/EtOAc); IR (ATR) ν_{max} 3056, 2941, 1328 cm^{-1}; ^{1}H NMR (500 MHz, $CDCl_3$) δ 7.32–7.28 (m, 4H), 7.21–7.18 (m, 1H), 2.62–2.57 (m, 2H), 1.89–1.79 (m, 3H), 1.59–1.51 (m, 3H), 1.38–1.36 (m, 1H), 1.20–1.14 (m, 1H); ^{13}C NMR (125 MHz, $CDCl_3$) δ 143.9 (d, J_{CP} = 26.1 Hz), 130.0 (d, J_{CP} = 14.0 Hz), 128.2 (d, J_{CP} = 4.2 Hz), 126.8 (s), 38.3 (d, J_{CP} = 5.2 Hz), 36.7 (d, J_{CP} = 10.1 Hz), 36.6 (d, J_{CP} = 3.1 Hz), 31.8 (d, J_{CP} = 18.0 Hz), 30.3 (s), 26.3 (d, J_{CP} = 21.4 Hz); ^{31}P NMR (121 MHz, $CDCl_3$) δ –14.6; HRMS (ESI) *m*/*z* $[M + H]^+$ calcd for $C_{12}H_{16}P$ 191.0990, found 191.0983.

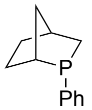

S4

2-Phenyl-2-phosphabicyclo[2.2.1]heptane (S4). Prepared using the reported method, in 95% yield (10:1 endo:exo).[3] R_f = 0.81 (3:1 hexanes/EtOAc); IR (ATR) ν_{max} 3056, 2981, 1328 cm^{-1}; ^{1}H NMR (500 MHz, $CDCl_3$) δ 7.36–7.31 (m, 4H), 7.26–7.23 (m, 1H), 2.65–2.57 (m, 2H), 2.03–1.94 (m, 1H), 1.75–1.49 (m, 4H), 1.41–1.36 (m, 1H), 1.38–1.36 (m, 1H), 1.00–0.95 (m, 1H); ^{13}C NMR (125 MHz, $CDCl_3$) δ 140.0 (d, J_{CP} = 25.5 Hz), 130.8 (d, J_{CP} = 14.9 Hz), 128.0 (d, J_{CP} = 4.6 Hz), 127.1 (s), 39.6 (d, J_{CP} = 15.6 Hz), 38.0 (d, J_{CP} = 1.9 Hz), 35.5 (d, J_{CP} = 13.8 Hz), 29.6 (d, J_{CP} = 2.2 Hz), 27.0 (d, J_{CP} = 8.9 Hz), 26.3 (d, J_{CP} = 4.2 Hz); ^{31}P NMR (121 MHz, $CDCl_3$) δ –7.7; HRMS (ESI) *m*/*z* $[M + H]^+$ calcd for $C_{12}H_{16}P$ 191.0990, found 191.0985.

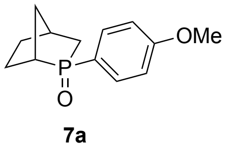

7a

2-(4-Methoxyphenyl)-2-phosphabicyclo[2.2.1]heptane 2-oxide (7a). Prepared using the method described above for the synthesis of the phosphine oxide **7**. TLC of the crude reaction mixture indicated that the exo/endo ratio was greater than 2.4:1. When run on a small scale (544 mg of the dimesylate **8**), separation of the exo- and endo-isomers **7a** and **7a′** was challenging; the

phosphine oxides **7a** and **7a′** were eventually separated through HPLC [Agilent Technologies 1260 Infinity; Agilent 10 Prep-Silica 150*30.0 column (isopropanol:EtOAc, 1:3)], providing 292.6 mg (62% yield) of **7a**. R_f = 0.61 (MeOH/DCM, 1:10); IR (ATR) ν_{max} 2952, 2875, 1597, 1505, 1257, 1169, 1114 cm^{-1}; ^1H NMR (500 MHz, $CDCl_3$) δ 7.69 (dd, *J* = 8.9, 10.3 Hz, 2H), 6.98 (dd, *J* = 1.9, 8.7 Hz, 2H), 3.84 (s, 3H), 2.70 (d, *J* = 29.6 Hz, 1H), 2.55–2.42 (m, 2H), 2.19–2.11 (m, 1H), 1.92–1.80 (m, 2H), 1.73–1.52 (m, 4H); ^{13}C NMR (125 MHz, $CDCl_3$) δ 162.1, 132.2 (d, J_{CP} = 10.8 Hz), 124.8 (d, J_{CP} = 94.7 Hz), 114.1 (d, J_{CP} = 12.2 Hz), 55.2, 37.9 (d, J_{CP} = 67.9 Hz), 36.7 (d, J_{CP} = 1.5 Hz), 36.6 (d, J_{CP} = 11.9 Hz), 33.0 (d, J_{CP} = 62.4 Hz), 29.2 (d, J_{CP} = 1.9 Hz), 20.8 (d, J_{CP} = 7.0 Hz); ^{31}P NMR (202 MHz, $CDCl_3$) δ 55.1; HRMS (ESI) *m/z* $[M + H]^+$ calcd for $C_{13}H_{18}OP$ 237.1044, found 237.1055.

We also attempted other routes for the preparation of the phosphine oxides **7** and **7′**:

Route 1

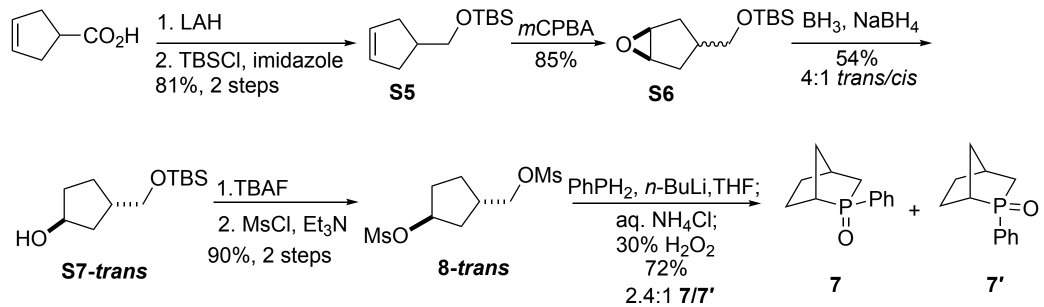

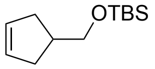

***tert*-Butyl(cyclopent-3-en-1-ylmethoxy)dimethylsilane (S5).** A solution of cyclopent-3-ene-1-carboxylic acid (2.24 g, 0.0200 mmol) in dry THF (20 mL) was added dropwise to a slurry of $LiAlH_4$ (1.14 g, 0.0300 mol, 1.5 equiv) in dry THF (10 mL) at 0 °C and the mixture was warmed to room temperature over 2 h. The heterogeneous mixture was cooled to 0 °C and then water (1.2 mL) was added very slowly, followed by 15% aqueous NaOH (1.2 mL) and water (3.6 mL). The mixture was stirred for 15 min. Na_2SO_4 was added and the mixture stirred for an additional 15 min. The organic phase was concentrated to give a a yellow oil, which was used directly in the next step.

TBSCl (4.53 g, 0.0300 mol, 1.5 equiv) and imidazole (3.40 g, 0.0500 mol, 2.5 equiv) were added in one batch to a solution of the yellow oil in dry DCM (20 mL) at room temperature. The mixture was stirred at room temperature overnight and then concentrated under reduced pressure. The residue was purified through FCC to yield **S5** as a colorless oil (81% over two steps). The spectral data have been reported previously.[4]

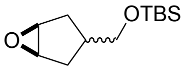

{(6-Oxabicyclo[3.1.0]hexan-3-yl)methoxy}(*tert*-butyl)dimethylsilane (S6). mCPBA (1.03 g, 6.00 mmol, 3.0 equiv) was added in several batches to a solution of **S5** (424 mg, 2.00 mmol) in dry DCM (20 mL) at room temperature. The mixture was stirred at room temperature overnight and concentrated under reduced pressure. The residue was purified through FCC to yield **S6** as a colorless oil (85% yield). The spectral data have been reported previously.[5]

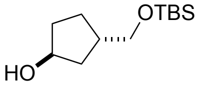

trans*-3-([(*tert*-Butyldimethylsilyl)oxy]methyl)cyclopentan-1-ol (S7-*trans*).** The secondary alcohol product **S7-*trans was prepared using the reported method, in 43% yield, from the epoxide **S6**.[6] In addition, **S7-*cis*** was also separated (11% yield).

S7-*trans*: R_f = 0.45 (3:1 hexanes/EtOAc); IR (ATR) ν_{max} 3348, 2953, 2928, 2855, 1253, 1096, 833 cm^{-1}; ^{1}H NMR (500 MHz, $CDCl_3$) δ 4.33 (s, 1H), 3.47 (s, 2H), 2.35 (s, 1H), 1.85–1.33 (m, 7H), 0.87 (s, 9H), 0.03 (s, 6H); ^{13}C NMR (125 MHz, $CDCl_3$) δ 73.9, 66.8, 39.6, 38.7, 35.1, 26.4, 25.9, 18.3, –5.3; HRMS (ESI) *m*/*z* [M + Na]$^+$ calcd for $C_{12}H_{26}O_2SiNa$ 253.1600, found 253.1610.

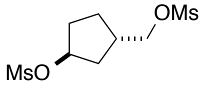

***trans*-3-([(Methylsulfonyl)oxy]cyclopentyl)methyl Methanesulfonate (8-*trans*).** TBAF (4.6 mL, 4.6 mmol, 1 M solution in THF, 2 equiv) was added dropwise to a solution of the silyl ether **S7** (0.53 g, 2.3 mmol) in dry THF (20 mL) at room temperature. The mixture was stirred at room temperature and the reaction monitored (TLC) for consumption of the starting material. The solution was concentrated under reduced pressure and the residue purified (FCC) to yield the diol as a colorless oil (95% yield). The spectral data matched those reported previously.[7]

A solution of methanesulfonyl chloride (0.39 mL, 5.0 mmol, 2.5 equiv) in DCM (20 mL) was added dropwise to a solution of the diol and dry Et_3N (0.69 mL, 5.0 mmol, 2.5 equiv) in DCM at 0 °C. The mixture was stirred at 0 °C for 30 min and then at room temperature after completion of the reaction (TLC). The reaction was quenched through the addition of 1 M HCl, saturated $NaHCO_3$, and saturated brine. The organic phase was dried (Na_2SO_4) and concentrated (rotary evaporation). The residue was purified through FCC on silica gel to provide **8-*trans*** in 90% yield over two steps.

Route 2

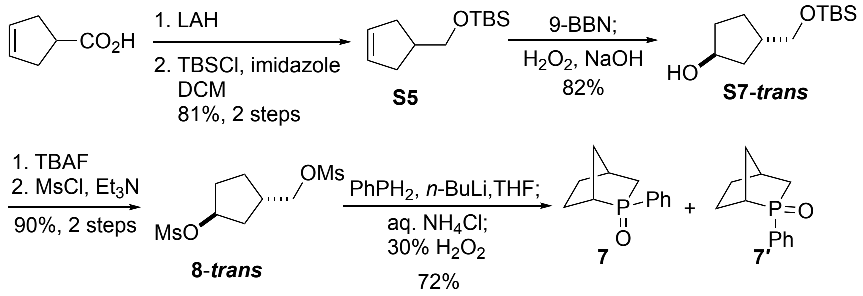

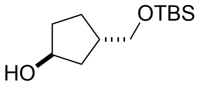

***trans*-3-([(*tert*-Butyldimethylsilyl)oxy]methyl)cyclopentan-1-ol (S7-*trans*).** A flame-dried flask was charged with the cyclohexene **S5** (212 mg, 1.0 mmol). The vessel was evacuated and backfilled with N_2; this process was repeated for a total of three times, followed by the addition of dry THF (10 mL). After cooling the vessel to 0 °C, 0.5 M 9-BBN in THF (4 mL, 2.0 mmol, 2.0 equiv) was added dropwise via syringe. The mixture was stirred for 3 h at 0 °C and then for 2 h at room temperature. 30% (w/w) Aqueous H_2O_2 (0.4 mL, 4 mmol, 4.0 equiv) was added to the

mixture at 0 °C and then it was stirred at room temperature for 2 h. 3 M NaOH (1.4 mL, 4 mmol, 4 equiv) was added and then the mixture and stirred for 30 min. The aqueous phase was extracted with EtOAc. The combined organic phases were washed (brine), dried (Na_2SO_4), filtered, and concentrated in vacuo. The residue was purified (FCC) to give **S7-*trans*** (82% yield) as a colorless oil.

Route 3

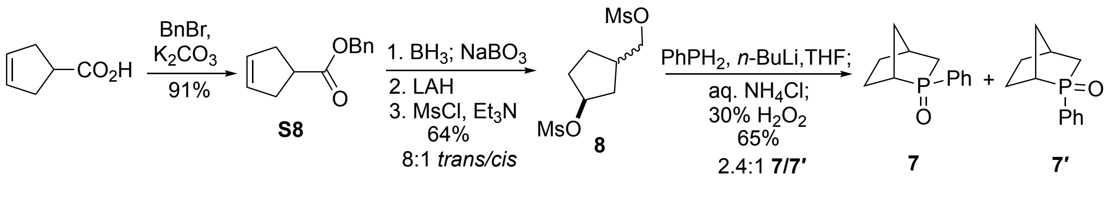

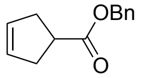

Benzyl Cyclopent-3-ene-1-carboxylate (S8). Prepared according to the reported procedure, in 91% yield.[8]

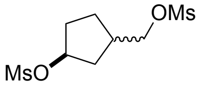

3-([(Methylsulfonyl)oxy]cyclopentyl)methyl Methanesulfonate (8). 1 M borane in THF (0.5 mL, 0.5 mmol, 0.5 equiv) was added dropwise via syringe to a solution of benzyl cyclopent-3-ene-1-carboxylate (202 mg, 1.0 mmol) in THF (5 mL) under Ar at 0 °C. The mixture was warmed to room temperature and stirred for 2 h. Water (1.15 mL) was added slowly, followed by sodium perborate tetrahydrate (154 mg, 1.0 mmol, 1.0 equiv), and then the mixture was stirred overnight. The mixture was concentrated and the aqueous phase extracted with EtOAc. The combined extracts were washed (brine), dried (Na_2SO_4), filtered, and concentrated to give the alcohol, which was used directly in the next step.

A solution of the alcohol in dry Et_2O (10 mL) was added dropwise to a slurry of $LiAlH_4$ (76 mg, 2.0 mmol, 2.0 equiv) in dry Et_2O (5 mL) at 0 °C. The mixture was warmed to room temperature and monitored (TLC) for consumption of the starting material. The mixture was cooled to 0 °C. Water (76 μL) was added slowly, followed by 15% aqueous NaOH (76 μL) and water (230 μL). The mixture was stirred at room temperature for 15 min. Na_2SO_4 was added and the mixture stirred for 15 min. The reaction mixture was filtered and the solids washed with dry DCM. The combined organic phases were concentrated to give the diol, which was used directly in the next step.

A solution of methanesulfonyl chloride (0.39 mL, 5.0 mmol, 2.5 equiv) in DCM (20 mL) was added dropwise to a solution of the diol and dry Et_3N (0.69 mL, 5.0 mmol, 2.5 equiv) in DCM at 0 °C. The mixture was stirred at 0 °C for 30 min and then at room temperature after the reaction had reached completion (TLC). The reaction was quenched through the addition of 1 M HCl, saturated $NaHCO_3$, and saturated brine. The organic phase was dried (Na_2SO_4) and

concentrated (rotary evaporation). The residue was purified through FCC on silica gel to provide **8** in 64% yield over three steps (*trans*:*cis* = 8:1; inseparable mixture).

Route 4

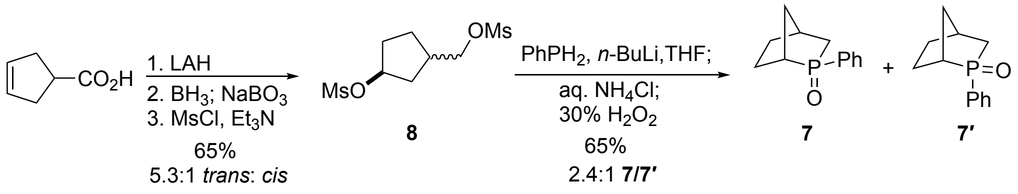

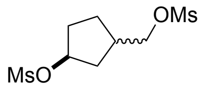

([(Methylsulfonyl)oxy]cyclopentyl)methyl Methanesulfonate (8). A solution of cyclopent-3-ene-1-carboxylic acid (10.0 g, 89.0 mmol) in dry Et_2O (20 mL) was added dropwise to a slurry of LAH (5.07 g, 134 mmol, 1.5 equiv) in dry Et_2O (140 mL) at 0 °C and then the mixture was warmed to room temperature and stirred for 2 h. The mixture was cooled to 0 °C and diluted with Et_2O. Water (5 mL) was added very slowly, followed by 15% aqueous NaOH (5 mL) and water (15 mL). The mixture was stirred for 15 min. Na_2SO_4 was added and the mixture stirred for an additional 15 min. The organic phase was concentrated to give a crude product, which was used directly in the next step.

A solution of the alcohol in dry THF (200 mL) at –20 °C was evacuated and backfilled with Ar three times. 1 M Borane in THF (44.5 mL, 44.5 mmol, 0.5 equiv) was added dropwise via syringe. The mixture was warmed to room temperature (monitored by TLC). A solution of sodium perborate tetrahydrate (54.8 g, 356 mmol, 4.0 equiv) was added and then the mixture was stirred at room temperature for 30 min. Na_2SO_4 (22 g) was added and then the mixture was stirred for 15 min. The mixture was then filtered and concentrated to obtain the diol, which was used without purification in the next step.

A solution of methanesulfonyl chloride (0.39 mL, 2.5 equiv) in DCM (20 mL) was added dropwise to a solution of the diol and dry Et_3N (0.69 mL, 2.5 equiv) in DCM at 0 °C. The mixture was stirred at 0 °C for 30 min and then at room temperature after the reaction had reached completion (TLC). The reaction was quenched through the addition of 1 M HCl, saturated $NaHCO_3$, and saturated brine. The organic phase was dried (Na_2SO_4) and concentrated (rotary evaporation). The residue was purified through FCC on silica gel to provide **8** in 65% yield over three steps (*trans*:*cis* = 5.3:1; inseparable mixture).

4. NMR Spectroscopic Studies of Silane-Mediated Reductions of Phosphine Oxides 4–7′, S1, S2, and 7a.

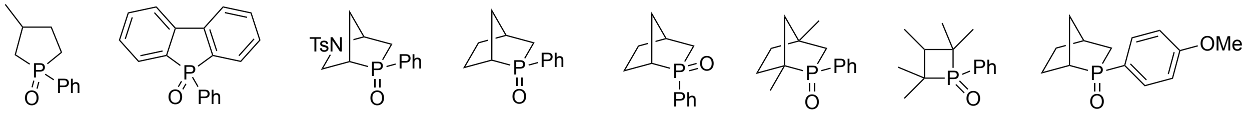

4 **5** **6** **7** **7′** **S1** **S2** **7a**

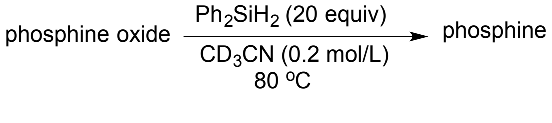

The phosphine oxides **4–7**, **7′**, **S1**, **S2** and **7a** (0.1 mmol) were dissolved in CD_3CN (0.5 mL) in an NMR tube and purged with Ar. Ph_2SiH_2 (20 equiv) was added to the NMR tubes, which were sealed with Teflon tape around the regular plastic cap. After 0, 20, 40, 80, 140, 200, 260, and 320 min, the conversions from the phosphine oxides to the phosphines were determined using ^{31}P NMR spectroscopy.

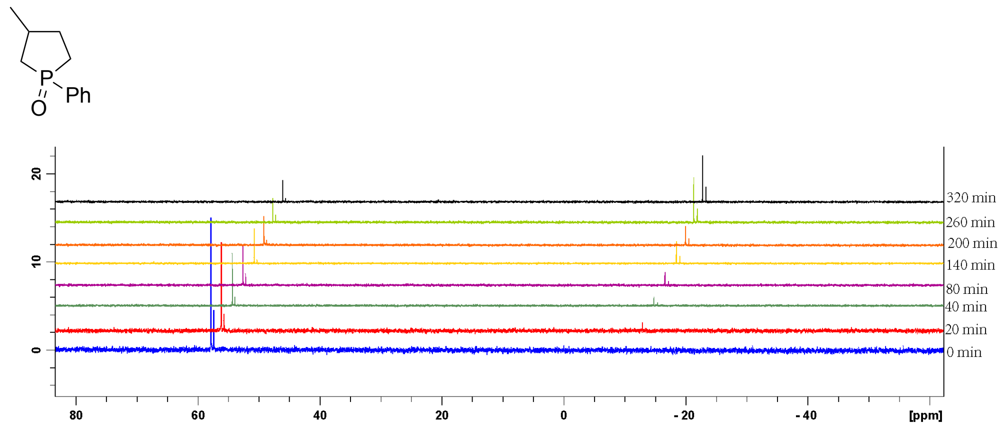

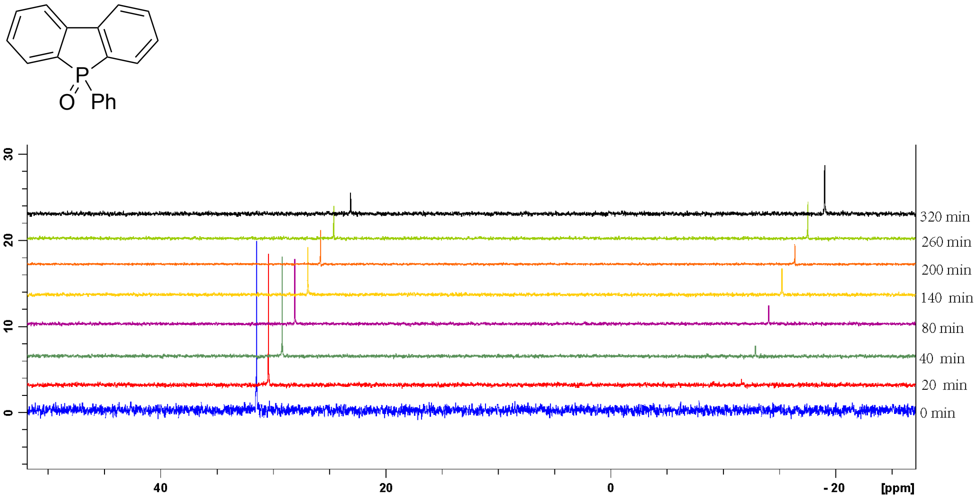

exo endo exo

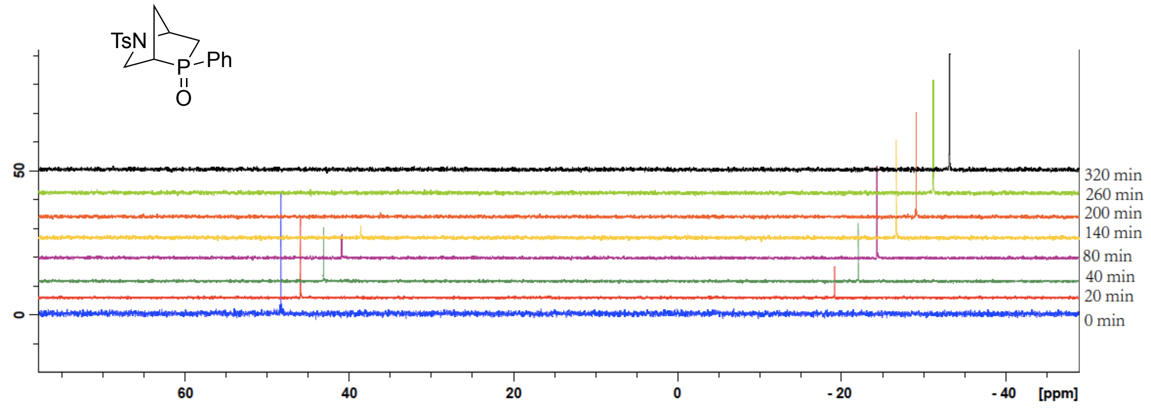

TsN
P
Ph
O
320 min
260 min
200 min
140 min
80 min
40 min
20 min
0 min
50
0
60
40
20
0
- 20
- 40
[ppm]

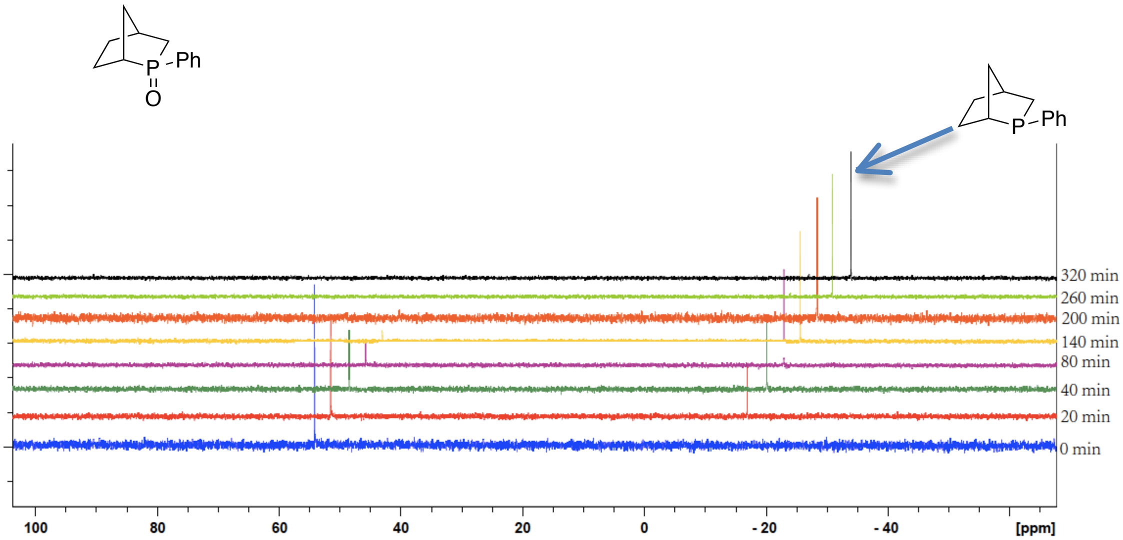

P
Ph
O
P
Ph
320 min
260 min
200 min
140 min
80 min
40 min
20 min
0 min
50
0
100
80
60
40
20
0
- 20
- 40
[ppm]

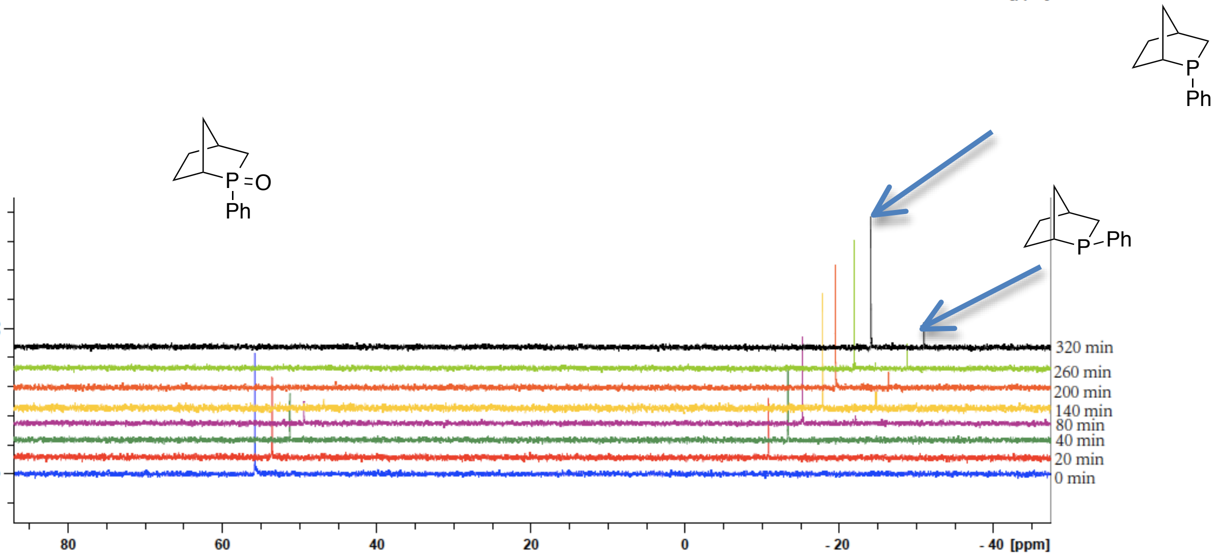

P
O
Ph
P
Ph
P
Ph
320 min
260 min
200 min
140 min
80 min
40 min
20 min
0 min
50
0
80
60
40
20
0
- 20
- 40
[ppm]

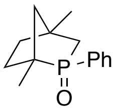

P
Ph
O

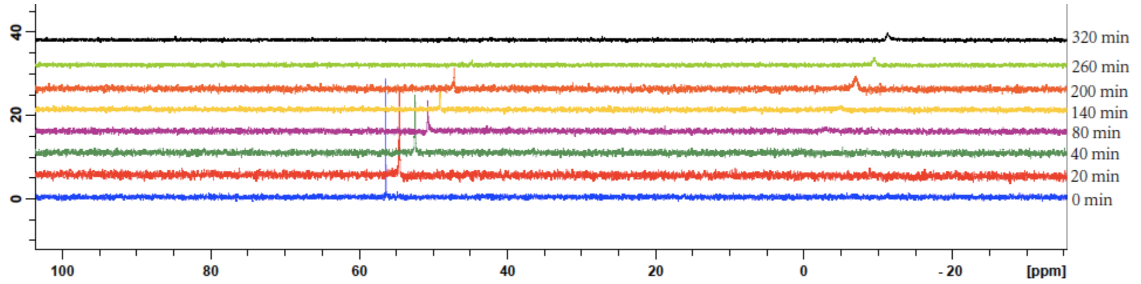

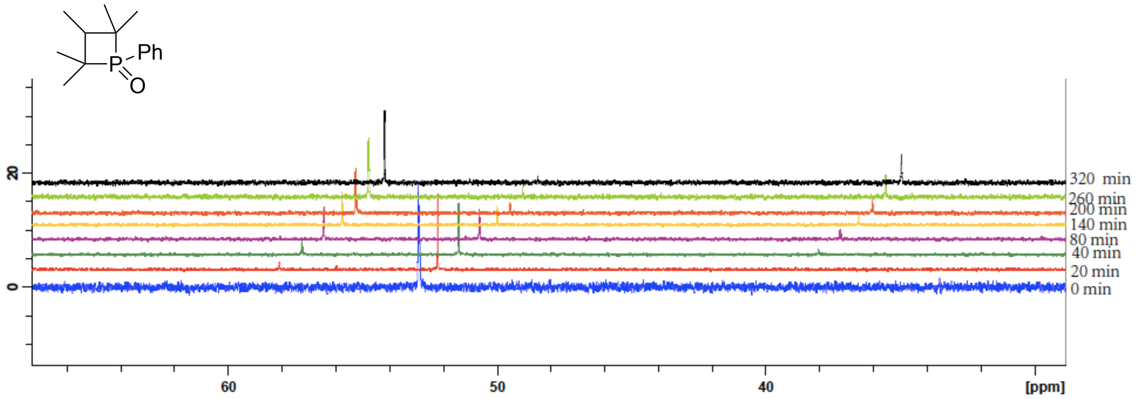

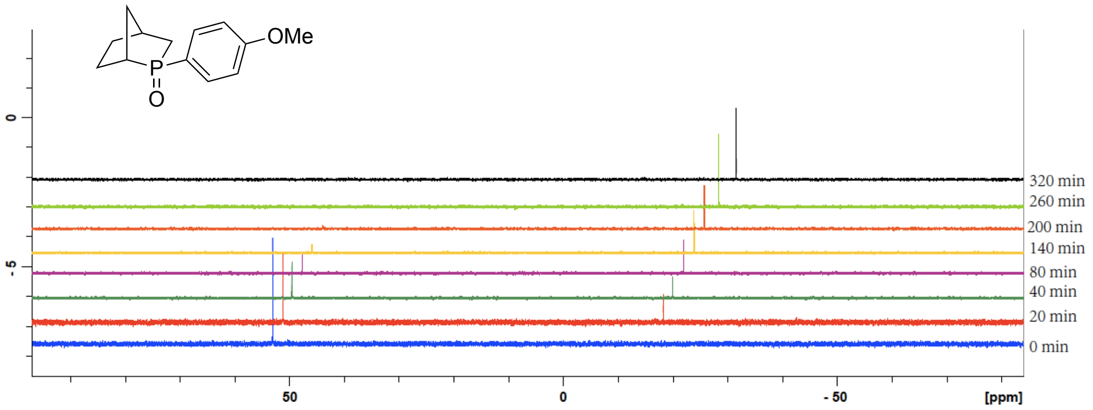

5. Kinetics Profiles, Trend Lines, and Fitting Equations for Substrates 4–7´, S2 and 7a

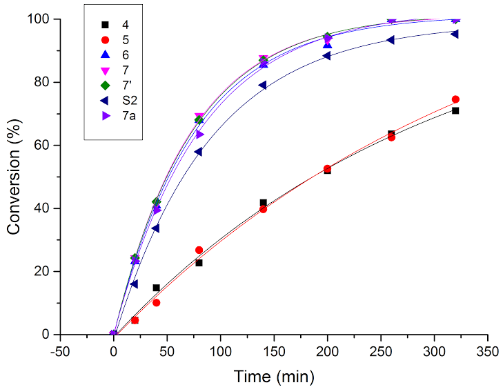

Time: X
Conversion: Y

Fitting function: Y = Y0 + A1*exp[-(X-X0)/t1]
Intial rate: R = -(A1/t1)*exp(X0/t1)

	Y0	X0	A1	t1	Initial rate
**4**	109.9	34.5	-98.8	303.0	0.37
**5**	126.8	43.6	-113.5	364.8	0.35
**6**	101.4	-0.3	-102.0	75.2	1.35
**7**	101.6	0.1	-101.8	72.0	1.42
**7'**	101.9	0.1	-101.9	73.4	1.39
**S2**	99.1	1.4	-99.3	90.3	1.12
**7a**	103.2	0.4	-102.6	81.5	1.27

Figure S6. Trend lines for kinetic profile of the phosphine oxides **4–7′**, **S2** and **7a**.

Trend lines were fitted for the phosphine oxides **4–7, 7′**, **S2** and **7a** with exponential decay functions, a result of pseudo-first-order kinetics profiles. The fitting parameters are listed in Figure S6, along with the rate equations for the reactions, determined by taking the derivatives of *Y* with respect to *X*. Initial rates were calculated by setting *X* to 0 in the rate equation; the initial rate for **4** was 0.37 min^{-1}, and that for **7** was 1.42 min^{-1}. The ratio of the two initial rates, r_4:r_7, is 1.0:4.0, corresponding to a difference in activation free energies of 0.81 kcal/mol, based on the Boltzmann distribution.

6. NMR Spectroscopic Studies of the γ-Umpolung–Wittig Reaction

The catalytic γ-Umpolung–Wittig Reaction under our final reaction condition was monitored using ^{1}H NMR and ^{31}P NMR spectroscopy. Firstly, we dissolved phosphine oxide **7** (6.2 mg, 0.03 mmol, 0.15 equiv) and Ph_2SiH_2 (74 µL, 0.40 mmol, 2 equiv) in toluene-d_8 (0.5 mL) in a 20 mL vial (prompt the phosphine oxide **7** to dissolve by sonication) and then transferred the resulting solution to an NMR tube. We recorded ^{1}H NMR and ^{31}P NMR at 0 min. And then the entire solution in the NMR tube was transferred back to the vial and reacted at 80 °C. We transferred the entire reaction mixture to an NMR tube and took ^{1}H NMR and ^{31}P NMR at 30 min and 1 h and transferred the solution back to the reaction vial after conducting the NMR studies. Then the neat *o*-sulfonamidobenzaldehyde **2a** (56 mg, 0.2 mmol) was added into the reaction vial. The allenoate **1a** (0.11 M in toluene-d_8, 0.60 mmol, 3 equiv) was added via a syring pump over 2 h and the reaction was run for 4 h after the addition of the allenoate. ^{1}H NMR and ^{31}P NMR were obtained by taking 0.4 mL of the reaction mixture at 2 h and placing the solution back to the vial, while at 3, 4, 5, 6, and 7 h the 0.4 mL solution was taken from the vial without putting it back into the vial. At 3 h, 0.2 mL of the reaction mixture contained 0.001 mmol of phosphorous species. The NMR sample solution was spiked with 0.001 mmol mixture of **7**/**7′** (14.2:1). This confirmed the assignment of ^{31}P peaks at 54.1 and 56.0 ppm as those belonging to phosphine oxide **7** and **7′**. At 8 h, 0.4 mL of the reaction mixture contained 0.001 mmol of phosphorous species. The NMR sample was spike with 0.0005 mol of **S3**. This confirmed the assignment of ^{31}P peak at –14.7 ppm as corresponding to phosphine **S3** and the ^{31}P peak at –11.9 ppm for phosphine **S4**.

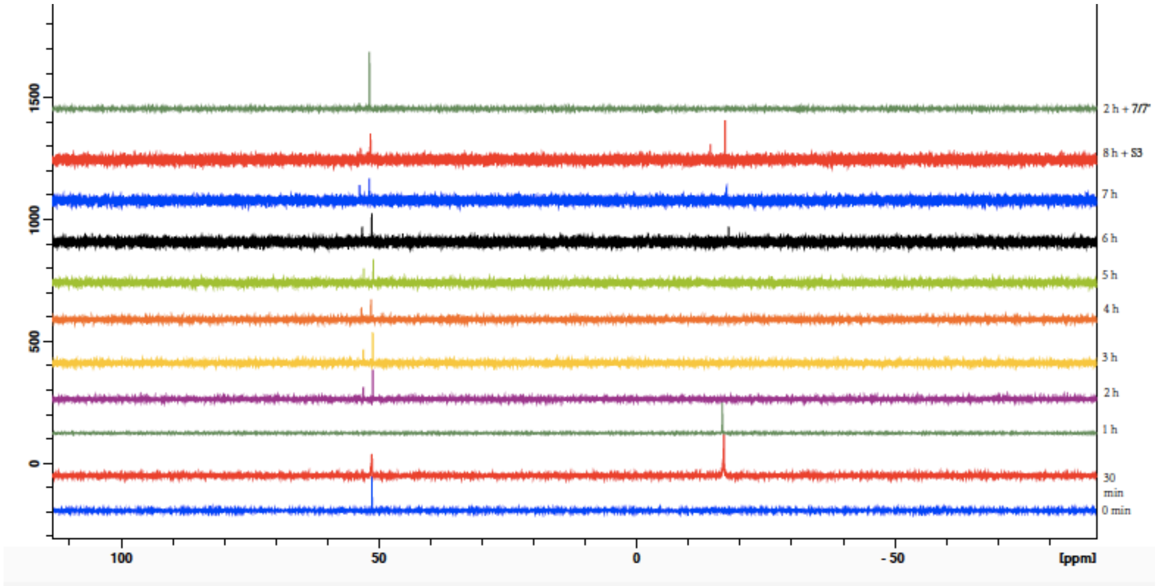

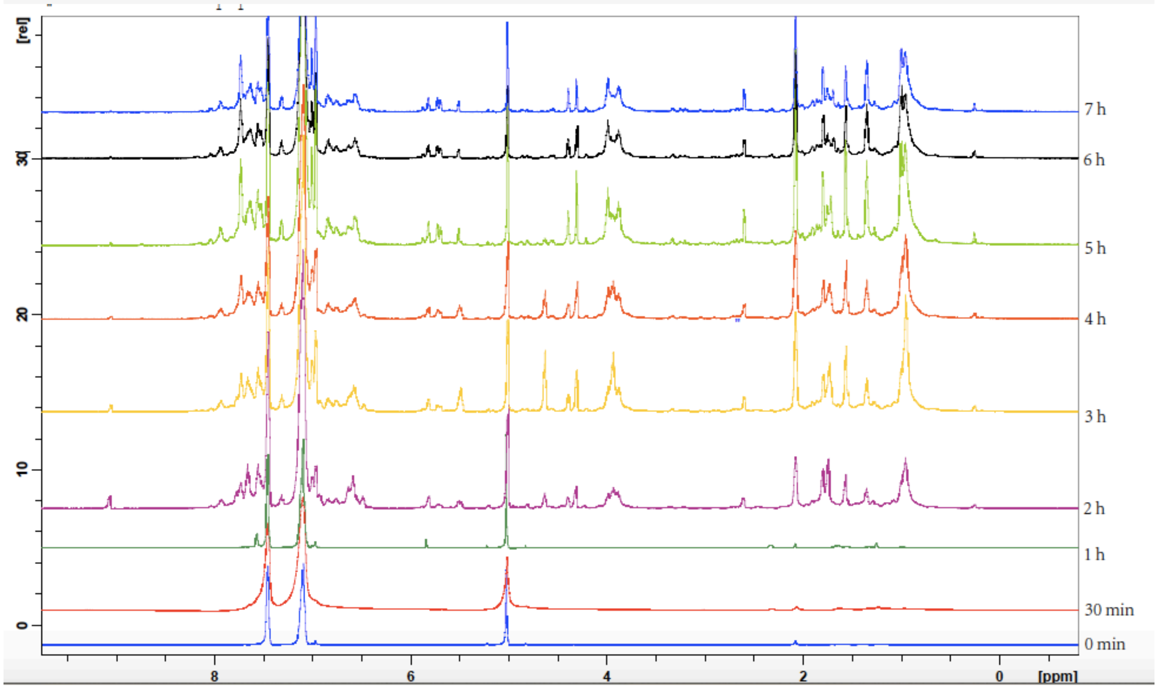

7. Preparation of Substrates

7.1 Allenoates

Ethyl 2,3-butadienoate,[9] benzyl 2,3-butadienoate,[10] 2,6-dimethylphenyl buta-2,3-dienoate,[11] 2-trimethylsilylethyl 2,3-butadienoate,[12] isopropyl buta-2,3-dienoate[12] neopentyl buta-2,3-dienoate,[12] and 2,6-diisopropylphenyl buta-2,3-dienoate[13] were prepared using reported methods.

7.2 General Procedure for Preparation of Substrates 2

The *o*-(*N*-tosyl)benzaldehyde **2a** was prepared from the corresponding *o*-aminobenzyl alcohol in two steps, without purification of any intermediates.[14] The *o*-(*N*-tosyl)benzaldehydes **2b–2l** were prepared from the corresponding acids in three steps, without purification of any intermediates.[14]

8. γ-Umpolung/Wittig Reactions

Table S4. Conditions Tested for the γ-Umpolung/Wittig Reactions

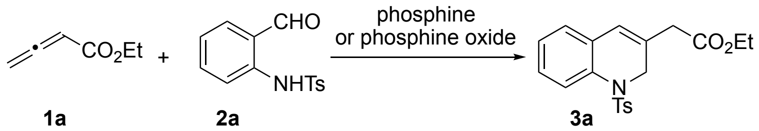

entry	phosphine (oxide)	solvent	C (mol/L)	silane	temp. (°C)	time (h)	isolated yield (%)
1	Ph_3P (100%)	toluent	0.05		rt	12	52
2	Ph_3P (100%)	benzene	0.05		rt	12	47
3	Ph_3P (100%)	CH_3CN	0.05		rt	12	NR
4	Ph_3P (100%)	$CHCl_3$	0.05		rt	12	67
5	Ph_3P (100%)	CH_2Cl_2	0.05		rt	12	31
6	Ph_3P (100%)	THF	0.05		rt	12	28
7	Ph_3P (120%)	$CHCl_3$	0.05		rt	12	91
8	**7** (30%)	CH_3CN	0.05	Ph_2SiH_2 (2 eq.)	80	48	<5
9	**7** (30%)	xylene	0.05	Ph_2SiH_2 (2 eq.)	80	48	NR
10	**7** (30%)	$CHCl_3$	0.05	Ph_2SiH_2 (2 eq.)	80	36	24
10	**7** (30%)	toluene	0.05	Ph_2SiH_2 (2 eq.)	80	4	81
11	**7** (30%)	toluene	0.05	Ph_2SiH_2 (1.5 eq.)	80	5	76
12	**7** (30%)	toluene	0.05	Ph_2SiH_2 (1 eq.)	80	8	65

General Procedure for Synthesis of the 1,2-Dihydroquinolines 3a–3r

A flame-dried 25-mL vial was charged with the phosphine oxide **7** (6.2 mg, 0.030 mmol, 0.15 equiv), Ph_2SiH_2 (74 μL, 0.40 mmol, 2 equiv), and dry toluene (0.5 mL) and then the mixture was purged with Ar and stirred at 80 °C for 1 h. Neat *o*-sulfonamidobenzaldehyde **2** (0.2 mmol) was added into the mixture and then the allenoate **1** (0.11 M in toluene, 0.60 mmol, 3 equiv) was added via a syringe pump over 2 h. After completion of the reaction (TLC), the solvent was evaporated under reduced pressure and the product purified through FCC on silica gel to yield the desired 1,2-dihydroquinolines **3a–3r**.

Characterization Data for the 1,2-Dihydroquinolines 3a–3r.

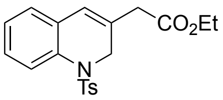

Ethyl 2-(1-Tosyl-1,2-dihydroquinolin-3-yl)acetate (3a). Pale yellow oil (65 mg, 88% yield); R_f = 0.52 (3:1 hexanes/EtOAc); IR (ATR) ν_{max} 3039, 2927, 1734, 1417, 1134, 1105 cm^{-1}; ^{1}H NMR (300 MHz, $CDCl_3$) δ 7.71–7.68 (m, 1H), 7.32–7.23 (m, 3H), 7.19–7.13 (m, 1H), 7.08–7.05 (m, 2H), 6.91 (dd, J = 1.5, 7.5 Hz, 1H), 5.89 (s, 1H), 4.44 (s, 1H), 4.15 (q, J = 7.2 Hz, 2H), 2.96 (s, 2H), 2.33 (s, 3H), 1.27 (t, J = 7.2 Hz, 3H); ^{13}C NMR (125 MHz, $CDCl_3$) δ 169.8, 158.2, 143.5, 135.7, 130.8, 130.2, 129.0, 128.0, 127.0, 126.9, 124.0, 112.9, 111.3, 61.1, 55.5, 48.6, 41.0, 21.5, 14.2; HRMS (ESI) m/z [M + Na]$^+$ calcd for $C_{20}H_{21}NO_4SNa$ 394.1089, found 394.1080.

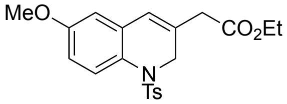

Ethyl 2-(6-Methoxy-1-tosyl-1,2-dihydroquinolin-3-yl)acetate (3b). Pale yellow oil (66 mg, 82% yield); R_f = 0.33 (3:1 hexanes/EtOAc); IR (ATR) ν_{max} 2981, 2937, 1732, 1493, 1162, 1091 cm^{-1}; ^{1}H NMR (500 MHz, $CDCl_3$) δ 7.60 (d, J = 8.8 Hz, 1H), 7.23 (d, J = 8.2 .Hz, 2H), 7.06 (d, J = 8.1 Hz, 2H), 6.80 (dd, J = 2.8, 8.8 Hz, 1H), 6.43 (d, J = 2.8 Hz, 1H), 5.79 (s, 1H), 4.39 (s, 1H), 4.13 (q, J = 7.2 Hz, 2H), 3.78 (s, 3H), 2.92 (s, 2H), 2.33 (s, 3H), 1.25 (t, J = 7.2 Hz, 3H); ^{13}C NMR (125 MHz, $CDCl_3$) δ 169.7, 158.2, 143.4, 135.7, 130.9, 130.3, 129.0, 128.0, 127.0, 126.9, 124.0, 113.0, 111.4, 61.1, 55.5, 48.6, 41.0, 21.5, 14.2; HRMS (ESI) m/z [M + Na]$^+$ calcd for $C_{21}H_{23}NO_5SNa$ 424.1195, found 424.1198.

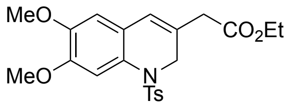

Ethyl 2-(6,7-Dimethoxy-1-tosyl-1,2-dihydroquinolin-3-yl)acetate (3c). Pale yellow oil (74.1 mg, 86% yield); R_f = 0.20 (3:1 hexanes/EtOAc); IR (ATR) ν_{max} 2980, 2937, 1732, 1509, 1163, 1092 cm^{-1}; ^{1}H NMR (300 MHz, $CDCl_3$) δ 7.27–7.26 (m, 2H), 7.23 (s, 1H), 7.06–7.04 (m, 2H), 6.40 (s, 1H), 5.75 (s, 1H), 4.37 (s, 1H), 4.12 (q, J = 7.2 Hz, 2H), 3.93 (s, 3H), 3.82 (s, 3 H), 2.91 (s, 3H), 1.25 (t, J = 7.2 Hz, 3H); ^{13}C NMR (75 MHz, $CDCl_3$) δ 169.9, 148.2, 147.7, 143.5, 135.7, 128.9, 127.3, 127.0 (2C), 123.6, 122.8, 110.5, 108.6, 61.1, 56.2, 56.0, 48.5, 40.9, 21.5, 14.2; HRMS (ESI) m/z [M + Na]$^+$ calcd for $C_{22}H_{25}NO_6SNa$ 454.1300, found 454.1294.

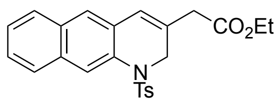

Ethyl 2-(1-Tosyl-1,2-dihydrobenzo[*g*]quinolin-3-yl)acetate (3d). Pale yellow oil (67 mg, 79% yield); R_f = 0.45 (3:1 hexanes/EtOAc); IR (ATR) ν_{max} 2982, 2922, 1731, 1349, 1164, 1091 cm^{-1}; ^{1}H NMR (300 MHz, $CDCl_3$) δ 8.20 (s, 1H), 7.92–7.89 (m, 1H), 7.78–7.75 (m, 1H), 7.54–7.45 (m, 2H), 7.38 (s, 1H), 7.34–7.30 (m, 2H), 7.08–7.05 (m, 2H), 6.15 (s, 1H), 4.57 (s, 2H), 4.21 (q, J = 7.2 Hz, 2H), 3.08 (s, 2H), 2.35 (s, 3H), 1.33 (t, J = 7.2 Hz, 3H); ^{13}C NMR (75 MHz, $CDCl_3$) δ 169.8, 143.5, 136.1, 132.0, 131.9, 131.1, 129.2, 128.3, 127.8, 127.5, 127.1, 126.4 (2C), 125.2, 124.8, 124.5, 61.2, 48.5, 41.2, 21.6, 14.3; HRMS (ESI) m/z [M + Na]$^+$ calcd for $C_{24}H_{23}NO_4SNa$ 444.1245, found 444.1240.

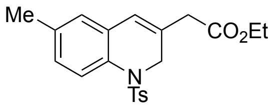

Ethyl 2-(6-Methyl-1-tosyl-1,2-dihydroquinolin-3-yl)acetate (3e). Pale yellow oil (65 mg, 85% yield); R_f = 0.45 (3:1 hexanes/EtOAc); IR (ATR) ν_{max} 2975, 2926, 1732, 1340, 1163, 1009 cm^{-1}; ^{1}H NMR (300 MHz, $CDCl_3$) δ 8.20 (s, 1H), 7.58–7.56 (m, 1H), 7.27–7.26 (m, 2H), 7.07–7.06 (m, 3H), 6.72 (s, 1H), 5.82 (s, 1H), 4.40 (s, 2H), 4.13 (q, J = 7.0 Hz, 2H), 2.94 (s, 2H), 2.33 (s, 3H), 2.30 (s, 3H), 1.26 (t, J = 7.0 Hz, 3H); ^{13}C NMR (125 MHz, $CDCl_3$) δ 169.8, 143.4, 136.5, 136.0, 131.5, 129.5, 129.0, 128.6, 126.9 (2C), 126.4, 124.1, 61.1, 48.5, 41.1, 21.5, 21.0, 14.2; HRMS (ESI) m/z [M + H]$^+$ calcd for $C_{21}H_{24}NO_4S$ 386.1426, found 386.1422.

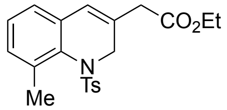

Ethyl 2-(8-Methyl-1-tosyl-1,2-dihydroquinolin-3-yl)acetate (3f). Pale yellow oil (75 mg, 98% yield); R_f = 0.45 (3:1 hexanes/EtOAc); IR (ATR) ν_{max} 2982, 2928, 1733, 1350, 1163, 1091 cm^{-1}; ^{1}H NMR (500 MHz, $CDCl_3$) δ 8.20 (s, 1H), 7.28–7.27 (m, 2H), 7.21–7.19 (m, 1H), 7.16–7.09 (m, 3H), 6.75 (dd, J = 7.5, 1.0 Hz, 1H), 5.74 (s, 1H), 4.55 (d, J = 18.5 Hz, 1H), 4.11 (qd, J = 7.5, 2.5 Hz, 2H), 4.06 (d, J = 17.5 Hz, 1H), 2.86 (d, J = 16.0 Hz, 1H), 2.80 (d, J = 16.0 Hz, 1H), 2.57 (s, 3H), 2.37 (s, 3H), 1.24 (t, J = 7.5 Hz, 3H); ^{13}C NMR (125 MHz, $CDCl_3$) δ 169.7, 143.6, 137.8, 136.0, 132.9, 131.3, 130.9, 130.3, 128.9, 127.5, 127.3, 124.9, 123.8, 61.1, 49.3, 41.1, 21.6, 19.5, 14.2; HRMS (ESI) m/z [M + Na]$^+$ calcd for $C_{21}H_{23}NO_4SNa$ 408.1245, found 408.1245.

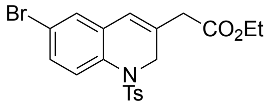

Ethyl 2-(6-Bromo-1-tosyl-1,2-dihydroquinolin-3-yl)acetate (3g). Pale yellow oil (49 mg, 55% yield); R_f = 0.51 (3:1 hexanes/EtOAc); IR (ATR) ν_{max} 2982, 2938, 1728, 1352, 1166, 1091 cm^{-1}; ^{1}H NMR (500 MHz, $CDCl_3$) δ 7.58–7.57 (m, 1H), 7.37–7.35 (m, 1H), 7.30–7.29 (m, 2H), 7.11–7.09 (m, 2H), 7.06–7.05 (m, 1H), 5.82 (s, 1H), 4.43 (s, 2H), 4.15 (q, J = 7.2 Hz, 2H), 2.96 (s, 2H), 2.35 (s, 3H), 1.27 (t, J = 7.2 Hz, 3H); ^{13}C NMR (125 MHz, $CDCl_3$) δ 169.4, 143.8, 135.7, 133.1, 131.4, 131.3, 130.6, 129.2, 129.0, 128.1, 126.9, 122.9, 120.0, 61.3, 48.3, 40.9, 21.6, 14.2; HRMS (ESI) m/z [M + Na]$^+$ calcd for $C_{20}H_{20}BrNO_4SNa$ 472.0194, found 472.0191.

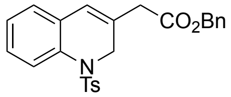

Benzyl 2-(1-Tosyl-1,2-dihydroquinolin-3-yl)acetate (3h). Pale yellow oil (69 mg, 80% yield); R_f = 0.52 (3:1 hexanes/EtOAc); IR (ATR) ν_{max} 3286, 3178, 1737, 1350, 1186, 1097 cm^{-1}; ^{1}H NMR (500 MHz, $CDCl_3$) δ 7.70 (d, J = 8.0 Hz, 1H), 7.39–7.32 (m, 5H), 7.28–7.24 (m, 3H), 7.17 (td, J = 7.5, 1.5 Hz, 1H), 7.04 (d, J = 7.5 Hz, 2H), 6.90 (dd, J = 7.5, 1.5 Hz, 1H), 5.88 (s, 1H), 5.12 (s, 2H), 4.43 (s, 2H), 3.01 (s, 2H), 2.31 (s, 3H); ^{13}C NMR (125 MHz, $CDCl_3$) δ 169.6, 143.5, 135.9, 135.4, 134.1, 129.6, 129.4, 129.0, 128.7, 128.5 (2C), 127.9, 126.9, 126.7, 126.6, 126.4, 124.2, 67.0, 48.4, 40.9, 21.5; HRMS (ESI) m/z [M + Na]$^+$ calcd for $C_{25}H_{23}NO_4SNa$ 456.1245, found 456.1236.

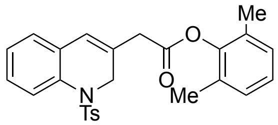

2,6-Dimethylphenyl 2-(1-Tosyl-1,2-dihydroquinolin-3-yl)acetate (3i). Pale yellow oil (70 mg, 78% yield); R_f = 0.55 (3:1 hexanes/EtOAc); IR (ATR) ν_{max} 2981, 2923, 1753, 1349, 1166, 1090 cm^{-1}; ^1H NMR (500 MHz, $CDCl_3$) δ 7.74 (d, J = 8.0 Hz, 1H), 7.32–7.26 (m, 3H), 7.17 (td, J = 7.5, 1.5 Hz, 1H), 7.08 (d, J = 8.0 Hz, 2H), 7.06 (s, 3H), 6.90 (dd, J = 7.5, 1.5 Hz, 1H), 6.05 (s, 1H), 4.54 (s, 2H), 3.27 (s, 2H), 2.34 (s, 3H), 2.11 (s, 6H); ^{13}C NMR (125 MHz, $CDCl_3$) δ 167.6, 147.9, 143.6, 136.0, 134.1, 129.9, 129.5, 129.1, 128.9, 128.7, 128.1, 127.0, 126.8, 126.6, 126.5, 126.2, 124.7, 48.7, 40.3, 21.5, 16.4; HRMS (ESI) m/z $[M + H]^+$ calcd for $C_{26}H_{26}NO_4S$ 448.1583, found 448.1586.

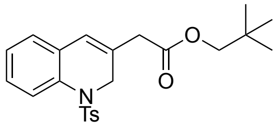

Neopentyl 2-(1-Tosyl-1,2-dihydroquinolin-3-yl)acetate (3j). Pale yellow oil (72 mg, 87% yield); R_f = 0.60 (3:1 hexanes/EtOAc); IR (ATR) ν_{max} 3064, 2981, 2935, 1727, 1350, 1165, 1091 cm^{-1}; ^1H NMR (500 MHz, $CDCl_3$) δ 7.69 (d, J = 8.0 Hz, 1H), 7.27–7.23 (m, 3H), 7.16 (td, J = 7.5, 1.5 Hz, 1H), 7.06 (d, J = 8.0 Hz, 2H), 6.90 (dd, J = 7.5, 1.5 Hz, 1H), 5.89 (s, 1H), 4.44 (s, 2H), 3.78 (s, 2H), 2.98 (s, 2H), 2.32 (s, 3H), 0.92 (s, 9H); ^{13}C NMR (125 MHz, $CDCl_3$) δ 169.9, 143.5, 135.9, 134.0, 129.7, 129.6, 129.0, 127.9, 126.9, 126.7, 126.6, 126.4, 124.0, 74.5, 48.5, 40.9, 31.3, 26.5, 21.5; HRMS (ESI) m/z $[M + H]^+$ calcd for $C_{23}H_{28}NO_4S$ 414.1739, found 414.1728.

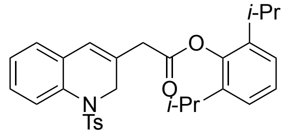

2,6-Diisopropylphenyl 2-(1-Tosyl-1,2-dihydroquinolin-3-yl)acetate (3k). Pale yellow oil (77 mg, 77% yield); R_f = 0.65 (3:1 hexanes/EtOAc); IR (ATR) ν_{max} 3061, 2965, 2930, 1753, 1351, 1165, 1093 cm^{-1}; ^1H NMR (300 MHz, $CDCl_3$) δ 7.72 (d, J = 6.3 Hz, 1H), 7.34–7.29 (m, 3H), 7.23–7.19 (m, 2H), 7.16–7.08 (m, 4H), 6.96 (dd, J = 7.5, 1.5 Hz, 1H), 6.05 (s, 1H), 4.53 (s, 2H), 3.28 (s, 2H), 2.83 (septet, J = 6.9 Hz, 2H), 2.35 (s, 3H), 1.17 (d, J = 6.9 Hz, 12H); ^{13}C NMR (125 MHz, $CDCl_3$) δ 168.6, 145.3, 143.7, 140.1, 136.0, 134.1, 129.5, 129.1, 128.9, 128.1, 127.0, 126.8, 126.7, 126.4, 124.7, 124.0, 48.7, 40.5, 27.6, 21.6; HRMS (ESI) m/z $[M + Na]^+$ calcd for $C_{30}H_{33}NO_4SNa$ 526.2028, found 526.2023.

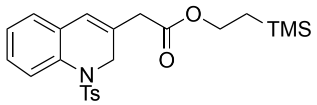

2-(Trimethylsilyl)ethyl 2-(1-Tosyl-1,2-dihydroquinolin-3-yl)acetate (3l). Pale yellow oil (76 mg, 86% yield); R_f = 0.65 (3:1 hexanes/EtOAc); IR (ATR) ν_{max} 3064, 2953, 2923, 1731, 1351, 1166, 1034 cm^{-1}; ^1H NMR (300 MHz, $CDCl_3$) δ 7.76–7.72 (m, 1H), 7.33–7.27 (m, 3H), 7.23–7.18 (m, 1H), 7.12–7.09 (m, 2H), 6.96–6.94 (m, 1H), 5.92 (s, 1H), 4.48 (s, 2H), 4.24–4.18 (m, 2H), 2.98 (s, 2H), 2.37 (s, 2H), 1.06–1.01 (m, 3H), 0.08 (s, 9H); ^{13}C NMR (125 MHz, $CDCl_3$) δ 169.9, 143.5, 135.9, 134.0, 129.7 (2C), 129.0, 127.8, 126.8, 126.9, 126.7, 126.6, 126.3, 124.0, 63.5, 48.4, 41.2, 21.6, 1.5; HRMS (ESI) m/z $[M + Na]^+$ calcd for $C_{23}H_{29}NO_4SSiNa$ 466.1484, found 466.1485.

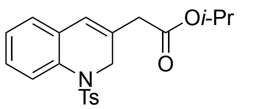

Isopropyl 2-(1-Tosyl-1,2-dihydroquinolin-3-yl)acetate (3m). Yellow oil (62 mg, 81% yield); R_f = 0.49 (3:1 hexanes/EtOAc); IR (ATR) ν_{max} 3071, 2982, 2930, 1726, 1350, 1164, 1093 cm^{-1}; ^{1}H NMR (500 MHz, $CDCl_3$) δ 7.71–7.69 (m, 1H), 7.28–7.24 (m, 3H), 7.18–7.15 (m, 1H), 7.07–7.05 (m, 2H), 6.92–6.90 (m, 1H), 5.87 (s, 1H), 5.00 (septet, J = 6.3 Hz, 1H), 4.43 (s, 2H), 2.93 (s, 2H), 2.33 (s, 3H), 1.24 (d, J = 6.3 Hz, 6H); ^{13}C NMR (125 MHz, $CDCl_3$) δ 169.3, 143.4, 135.9, 134.0, 129.8, 129.7, 129.0, 127.8, 126.9, 126.7, 126.6, 126.3, 123.9, 68.7, 48.4, 41.4, 21.8, 21.5; HRMS (ESI) m/z [M + Na]$^+$ calcd for $C_{21}H_{23}NO_4SNa$ 408.1245, found 408.1249.

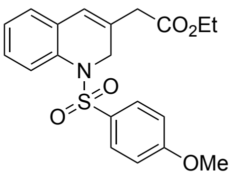

Ethyl 2-(1-[(4-Methoxyphenyl)sulfonyl]-1,2-dihydroquinolin-3-yl)acetate (3n). Yellow oil (63 mg, 82% yield); R_f = 0.53 (3:1 hexanes/EtOAc); IR (ATR) ν_{max} 2970, 1737, 1596, 1353, 1260, 1163 cm^{-1}; ^{1}H NMR (300 MHz, $CDCl_3$) δ 7.75–7.73 (m, 1H), 7.38–7.18 (m, 4H), 6.97–6.94 (m, 1H), 6.80–6.75 (m, 2H), 5.96 (s, 1H), 4.48 (s, 2H), 4.19 (q, J = 7.2 Hz, 2H), 3.83 (s, 3H), 3.02 (s, 2H), 1.31 (t, J = 7.2 Hz, 3H); ^{13}C NMR (125 MHz, $CDCl_3$) δ 169.8, 163.0, 134.1, 130.6, 129.8, 129.7, 129.0, 127.9, 126.7 (2C), 126.3, 124.0, 113.5, 61.2, 55.5, 48.4, 41.0, 14.2; HRMS (ESI) m/z [M + H]$^+$ calcd for $C_{20}H_{22}NO_5S$ 388.1219, found 388.1217.

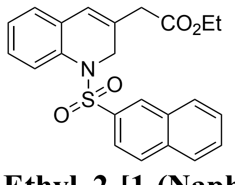

Ethyl 2-[1-(Naphthalen-2-ylsulfonyl)-1,2-dihydroquinolin-3-yl]acetate (3o). Pale yellow oil (68 mg, 84% yield); R_f = 0.52 (3:1 hexanes/EtOAc); IR (ATR) ν_{max} 3060, 2981, 2933, 1732, 1347, 1164, 1075 cm^{-1}; ^{1}H NMR (500 MHz, $CDCl_3$) δ 8.00 (d, J =1.0 Hz, 1H), 7.82 (d, J = 8.0 Hz, 1H), 7.79–7.76 (m, 2H), 7.71 (d, J = 8.5 Hz, 1H), 7.61–7.57 (m, 1H), 7.55–7.52 (m, 1H), 7.34–7.32 (m, 1H), 7.32–7.29 (m, 1H), 7.18 (td, J = 7.5, 1.5 Hz, 1H), 6.85 (dd, J = 7.5, 1.0 Hz, 1H), 5.75 (s, 1H), 4.51 (s, 2H), 4.10 (q, J =7.5 Hz, 2H), 2.89 (s, 2H), 1.58 (s, 2H), 1.23 (t, J = 7.5 Hz, 3H); ^{13}C NMR (125 MHz, $CDCl_3$) δ 169.7, 135.8, 134.8, 134.0, 131.8, 129.7 (2C), 129.1, 128.7, 128.5, 128.2, 128.0, 127.8, 127.3, 126.9, 126.6, 126.4, 124.1, 122.4, 61.1, 48.5, 40.8, 14.2; HRMS (ESI) m/z [M + Na]$^+$ calcd for $C_{23}H_{21}NO_4SNa$ 430.1089, found 430.1083.

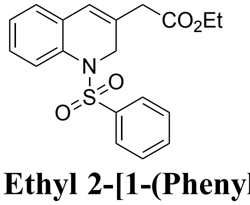

Ethyl 2-[1-(Phenylsulfonyl)-1,2-dihydroquinolin-3-yl]acetate (3p). Pale yellow oil (55 mg, 83% yield); R_f = 0.52 (3:1 hexanes/EtOAc); IR (ATR) ν_{max} 3063, 2984, 1732, 1352, 1168, 1091 cm^{-1}; ^{1}H NMR (500 MHz, $CDCl_3$) δ 7.71 (d, J = 8.0 Hz, 1H), 7.47–7.44 (m, 1H), 7.40–7.38 (m, 2H), 7.30–7.28 (m, 3H), 7.18 (td, J = 7.5, 1.0 Hz, 1H), 6.91 (dd, J = 7.5, 1,5 Hz, 1H), 5.86 (s, 1H), 4.45

(s, 2H), 4.15 (q, J = 7.5 Hz, 2H), 2.95 (s, 2H), 1.27 (t, J = 7.0 Hz, 3H); ^{13}C NMR (125 MHz, $CDCl_3$) δ 169.7, 138.7, 133.9, 132.8, 129.7 (2C), 128.4, 127.9, 126.9 (2C), 126.7, 126.4, 124.0, 61.2, 48.4, 41.0, 14.2; HRMS (ESI) m/z $[M + Na]^+$ calcd for $C_{19}H_{19}NO_4SNa$ 380.0933, found 380.0929.

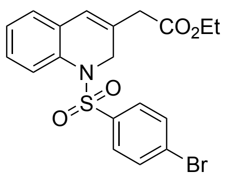

Ethyl 2-(1-[(4-Bromophenyl)sulfonyl]-1,2-dihydroquinolin-3-yl)acetate (3q). Pale yellow oil (37 mg, 47% yield); R_f = 0.52 (3:1 hexanes/EtOAc); IR (ATR) ν_{max} 3072, 2982, 1731, 1355, 1167, 1068 cm^{-1}; ^{1}H NMR (500 MHz, $CDCl_3$) δ 7.70 (d, J = 8.0 Hz, 1H), 7.42–7.39 (m, 2H), 7.29–7.27 (m, 1H), 7.25–7.23 (m, 2H), 7.19 (td, J = 7.5, 1.0 Hz, 1H), 6.94 (dd, J = 7.5, 1.5 Hz, 1H), 5.93 (s, 1H), 4.45 (s, 2H), 4.16 (q, J =7.5 Hz, 2H), 2.99 (s, 2H), 1.28 (t, J = 7.5 Hz, 3H); ^{13}C NMR (125 MHz, $CDCl_3$) δ 169.6, 137.7, 133.6, 131.6, 129.8, 129.7, 128.5, 128.1, 127.8, 127.1, 126.6, 126.5, 124.2, 61.2, 48.4, 40.8, 14.2; HRMS (ESI) m/z $[M + Na]^+$ calcd for $C_{19}H_{18}BrNO_4SNa$ 458.0038, found 458.0036.

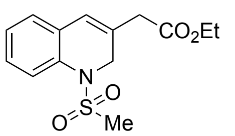

Ethyl 2-[1-(Methylsulfonyl)-1,2-dihydroquinolin-3-yl]acetate (3r). Pale yellow oil (25 mg, 43% yield); R_f = 0.52 (3:1 hexanes/EtOAc); IR (ATR) ν_{max} 3015, 2926, 1731, 1343, 1157, 1077 cm^{-1}; ^{1}H NMR (300 MHz, $CDCl_3$) δ 7.68–7.65 (m, 1H), 7.34–7.28 (m, 1H), 7.27–7.24 (m, 1H), 7.18–7.15 (m, 1H), 6.50 (s, 1H), 5.93 (s, 1H), 4.44 (s, 2H), 4.26 (q, J = 6.9 Hz, 2H), 3.30 (s, 2H), 2.81 (s, 3H), 1.35 (t, J = 6.9 Hz, 3H); ^{13}C NMR (125 MHz, $CDCl_3$) δ 170.3, 134.1, 131.0, 129.1, 128.3, 126.9, 126.7, 126.5, 125.2, 61.4, 48.0, 39.9, 37.2, 14.2; HRMS (ESI) m/z $[M + Na]^+$ calcd for $C_{14}H_{17}NO_4SNa$ 318.0776, found 318.0781.

9. Synthesis of the Furanoquinoline 13

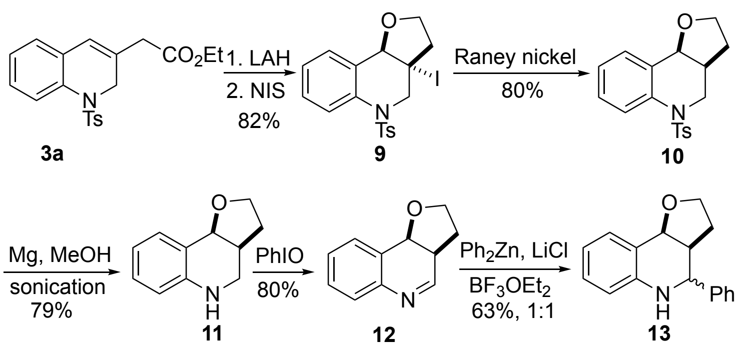

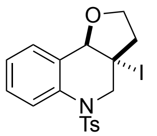

3α-Iodo-5-tosyl-2,3,3α,4,5,9β-hexahydrofuro[3,2-*c*]quinoline (9). A solution of **3a** (7.42 g, 20.0 mmol) in dry THF was added dropwise to a slurry of $LiAlH_4$ (1.14 g, 30.0 mmol, 1.5 equiv) in dry THF at 0 °C and then the mixture was warmed to room temperature over 2 h. The heterogeneous mixture was cooled to 0 °C and then water (1.14 mL) was added very slowly, followed by 15% aqueous NaOH (1.14 mL) and water (3.4 mL). The mixture was stirred at room temperature for 15 min. After adding a drying reagent (Na_2SO_4) and stirring for another 15 min, the whole mixture was filtered to remove the salts. The organic phase was dried and concentrated in vacuo to give a crude yellow oil that was used in the next step without purification.

N-Iodosuccinimide (9.0 g, 40 mmol, 2 equiv) was added in one batch to a solution of the yellow oil in dry DCM at room temperature. The mixture was stirred at room temperature for 1.5 h and monitored (TLC) for consumption of the starting material. The solution was concentrated under reduced pressure and then the residue was purified (FCC) to yield the iodofuran **9** (1.82 g, 82% over two steps). R_f = 0.70 (3:1 hexanes/EtOAc); IR (ATR) ν_{max} 3058, 2981, 2875, 1491, 1349, 1162, 1090 cm^{-1}; ^{1}H NMR (500 MHz, $CDCl_3$) δ 7.81–7.80 (m, 2H), 7.44–7.43 (m, 1H), 7.36–7.35 (m, 1H), 7.32–7.30 (m, 2H), 7.21–7.17 (m, 1H), 7.09–7.06 (m, 1H), 5.09 (s, 1H), 4.43 (d, *J* = 13.5 Hz, 1H), 4.04–3.98 (m, 2H), 3.73 (d, *J* = 13.5 Hz, 2H), 2.85–2.80 (m, 1H), 2.61–2.55 (m, 1H), 2.41 (s, 3H); ^{13}C NMR (125 MHz, $CDCl_3$) δ 144.1, 137.8, 136.2, 131.4, 130.0, 129.1, 126.9, 124.2, 124.0, 119.0, 86.2, 66.1, 56.3, 42.9, 39.5, 21.6; HRMS (ESI) *m*/*z* $[M + Na]^+$ calcd for $C_{18}H_{18}INO_3SNa$ 477.9950, found 477.9948.

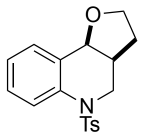

5-Tosyl-2,3,3a,4,5,9b-hexahydrofuro[3,2-*c*]quinoline (10). The sulfonamide **9** (1.2 g, 2.64 mmol) was dissolved in methanol (400 mL) in a round-bottom flask equipped with a stirrer bar. A suspension of Raney Ni in water [Raney Ni 4200 (Aldrich), 20 mL] was added directly to the solution without removal of the water. The mixture was stirred vigorously (to keep the Raney Ni suspended) until the starting material was consumed (TLC). The supernatant was decanted from the solid Raney Ni into a separatory funnel containing saturated aqueous NaCl. The solids were rinsed three times with EtOAc; the rinses were also added to the separatory funnel. After separation of the layers, the aqueous phase was extracted three times with EtOAc. The combined organic phases were washed again (brine) and dried (Na_2SO_4). After filtration and concentration in vacuo, the crude product was purified (FCC) to yield a white solid (0.69 g, 80%). The spectral data matched those reported previously.[15]

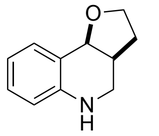

2,3,3a,4,5,9b-Hexahydrofuro[3,2-*c*]quinoline (11). Prepared using the reported method, in 79% yield.[16] R_f = 0.42 (3:1 hexanes/EtOAc); IR (ATR) ν_{max} 3383, 2962, 2938, 2872, 1612, 1498, 1276 cm^{-1}; ^{1}H NMR (500 MHz, $CDCl_3$) δ 7.34–7.32 (m, 1H), 7.10–7.08 (m, 1H), 6.76–6.73 (m, 1H), 6.61–6.59 (m, 1H), 4.58 (d, *J* = 5.4 Hz, 1H), 3.96 (m, 1H), 3.83 (m, 1H), 3.14 (dd, *J* = 11.1, 5.1 Hz, 1H), 2.85 (app. t, *J* = 11.0 Hz, 1H), 2.48–2.41 (m, 1H), 2.28–2.21 (m, 1H), 1.79–1.73 (m, 1H); ^{13}C

NMR (125 MHz, $CDCl_3$) δ 145.4, 131.4, 128.8, 120.5, 118.1, 114.8, 75.5, 65.3, 43.2, 36.1, 29.8; HRMS (ESI) m/z $[M + H]^+$ calcd for $C_{11}H_{14}NO$ 176.1075, found 176.1080.

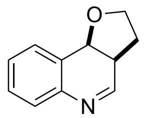

2,3,3a,9b-Tetrahydrofuro[3,2-*c*]quinoline (12). A solution of the quinoline **11** (175 mg, 1 mmol) and PhIO (1.19 g, 5.4 mmol, 5.4 equiv) in DCM was stirred at room temperature and monitored (TLC) for consumption of the starting material. The resulting solution was concentrated in vacuo. Purification of the residue (FCC) afforded the quinoline **12** (138 mg, 80%). R_f = 0.52 (1:1 hexanes/EtOAc); IR (ATR) ν_{max} 3004, 2987, 2934, 1728, 1676, 1276, 1260 cm^{-1}; ^1H NMR (500 MHz, $CDCl_3$) δ 9.04 (s, 1H), 7.32–7.31 (m, 1H), 7.21–7.16 (m, 1H), 6.99–6.96 (m, 1H), 6.78–6.76 (m, 1H), 4.80 (d, J = 6.2 Hz, 1H), 3.82–3.79 (m, 2H), 3.11–3.07 (m, 1H), 2.56–2.51 (m, 1H), 2.40–2.36 (m, 1H); ^{13}C NMR (125 MHz, $CDCl_3$) δ 171.5, 136.3, 130.0, 129.9, 123.5, 120.3, 115.6, 76.8, 66.3, 43.7, 30.1; HRMS (ESI) m/z $[M + H]^+$ calcd for $C_{11}H_{12}NO$ 174.0919, found 174.0914.

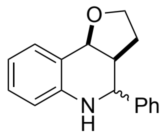

4-Phenyl-2,3,3a,4,5,9b-hexahydrofuro[3,2-*c*]quinoline (13). $BF_3 \bullet OEt_2$ (25 µL, 0.2 mmol, 1.0 equiv) and fresh PhZnCl reagent (2 mL, 1.0 mmol, 5.0 equiv) [prepared from $ZnCl_2$ (0.26 g) and PhLi (1 mL, 1.9 M in dibutyl ether) in THF (2.8 mL) as a 0.5 M solution] were added sequentially to a solution of the imine **14** (34.6 mg, 0.2 mmol) in dry THF (5 mL) at 0 °C.[16] The reaction mixture partitioned between ethyl acetate and saturated NH_4Cl. The aqueous phase was extracted three times with EtOAc and the combined organic phases were washed (brine), dried (Na_2SO_4), and concentrated in vacuo. The residue was purified through FCC to yield **13-*cis*** and **13-*trans*** in 63% yield (1:1). The cis and trans products were differentiated by comparing their data with that reported in the literature.[17]

13-*cis*: ^1H NMR (500 MHz, $CDCl_3$) δ 7.48–7.46 (m, 2H), 7.40–7.35 (m, 3H), 7.33–7.30 (m, 1H), 7.11–7.08 (m, 1H), 6.83–6.80 (m, 1H), 6.61–6.60 (m, 1H), 5.28 (d, J = 8.0 Hz, 1H), 4.71–4.70 (m, 1H), 3.85–3.80 (m, 2H), 3.74–3.69 (m, 1H), 2.82–2.76 (m, 1H), 2.25–2.17 (m, 1H), 1.16–1.50 (m, 1H); ^{13}C NMR (125 MHz, $CDCl_3$) δ 145.0, 142.2, 130.1, 128.7, 128.4, 127.7, 126.5, 122.7, 119.2, 114.9, 76.0, 66.8, 57.5, 45.8, 24.7.

13-*trans*: ^1H NMR (500 MHz, $CDCl_3$) δ 7.45–7.43 (m, 2H), 7.41–7.38 (m, 3H), 7.36–7.33 (m, 1H), 7.14–7.11 (m, 1H), 6.82–6.78 (m, 1H), 6.64–6.62 (m, 1H), 4.60 (d, J = 8.0 Hz, 1H), 4.14 (s, 1H), 4.06–4.01 (m, 1H), 3.86–3.79 (m, 2H), 2.49–2.44 (m, 1H), 2.05–1.98 (m, 1H), 1.75–1.69 (m, 1H); ^{13}C NMR (125 MHz, $CDCl_3$) δ 145.4, 141.7, 131.2, 129.0, 128.7, 128.3 (2C), 128.2, 120.0, 118.4, 114.7, 76.2, 65.2, 57.8, 43.4, 28.8.

10. References

1. (a) Krenske, E. H. *J. Org. Chem.* **2012**, *77*, 1. (b) Krenske, E. H. *J. Org. Chem.* **2012**, *77*, 3969.
2. $PhPH_2$ was commercially available, but prepared through LAH-mediated reduction of $PhPCl_2$. Because the purity of $PhPH_2$ is important for dialkylation, the phosphine required distillation. While distilling, use of a cow adapter was necessary.
3. Henry, C. E.; Xu, Q.; Fan, Y. C.; Martin, T. J.; Belding, L.; Dudding, T. Kwon, O. *J. Am. Chem. Soc.* **2014**, *136*, 11890.
4. Wainwright, P.; Maddaford, A.; Bissell, R.; Fisher, R.; Leese, D.; Lund, A.; Runcie, K.; Dragovich, P. S.; Gonzalez, J.; Kung, P.; Middleton, D. S.; Pryde, D.; Stephenson, P.; Sutton, S. *Synlett* **2005**, *5*, 765.
5. Agrofoglio, L.; Condom, R.; Guedj, R. *Tetrahydron Lett.* **1992**, *33*, 5503.
6. Henkens, R.; Sturtevant, J. M. *J. Am. Chem. Soc.* **1968**, *90*, 2669.
7. Marcé, P.; Díaz, Y.; Matheu, M. I.; Castillón, S. *Org. Lett.* **2008**, *10*, 4735.
8. Amjad, A.; Man-Chu, L. M.; Edward, M.; Lin, Y. PCT Int. Appl. (2011), WO 2011100384 A1 Aug 18, 2011.
9. Clavier, H.; Jeune, K. L.; Riggi, I.; Tenglia, A.; Buono, G. *Org. Lett.* **2011**, *13*, 308.
10. Saunders, L. B.; Cowen, B. J.; Miller, S. J. *Org. Lett.* **2010**, *12*, 4800.
11. Himbert, G.; Fink, D. *Tetrahydron Lett.* **1985**, *26*, 4363.
12. Zhu, X.; Henry, C. E.; Wang, J.; Dudding, T.; Kwon, O. *Org. Lett.* **2005**, *7*, 1387.
13. Himbert, G.; Fink, D.; Diehl, K.; Rademacher, P.; Bittner, A. J. *Chem. Ber.* **1989**, *122*, 1161.
14. (a) Fonseca, M. H.; Eibler, E.; Zabelb, M.; Konig, B. *Tetrahedron: Asymmetry* **2003**, *14*, 1989. (b) Theeraladanon, C.; Arisawa, M.; Nishida, A.; Nakagawa, M. *Tetrahedron* **2004**, *60*, 3017. (c) Chernyak, D.; Chernyak, N.; Gevorgyan, V. *Adv. Synth. Catal.* **2010**, *352*, 961.
15. Lindsay, V. N. G.; Viart, H. M. F.; Sarpong, R. *J. Am. Chem. Soc.* **2015**, *137*, 8368.
16. Pilli, R. A.L.; Robello, G. *J. Braz. Chem. Soc.* **2004**, *15*, 938.
17. Yu, J.; Jiang, H.-J; Zhou, Y.; Luo, S.-W.; Gong, L.-Z. *Angew. Chem. Int. Ed.* **2015**, *54*, 11209.

11. ^{1}H, ^{13}C, and ^{31}P NMR Spectra

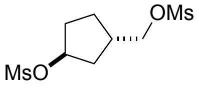

8-*trans*

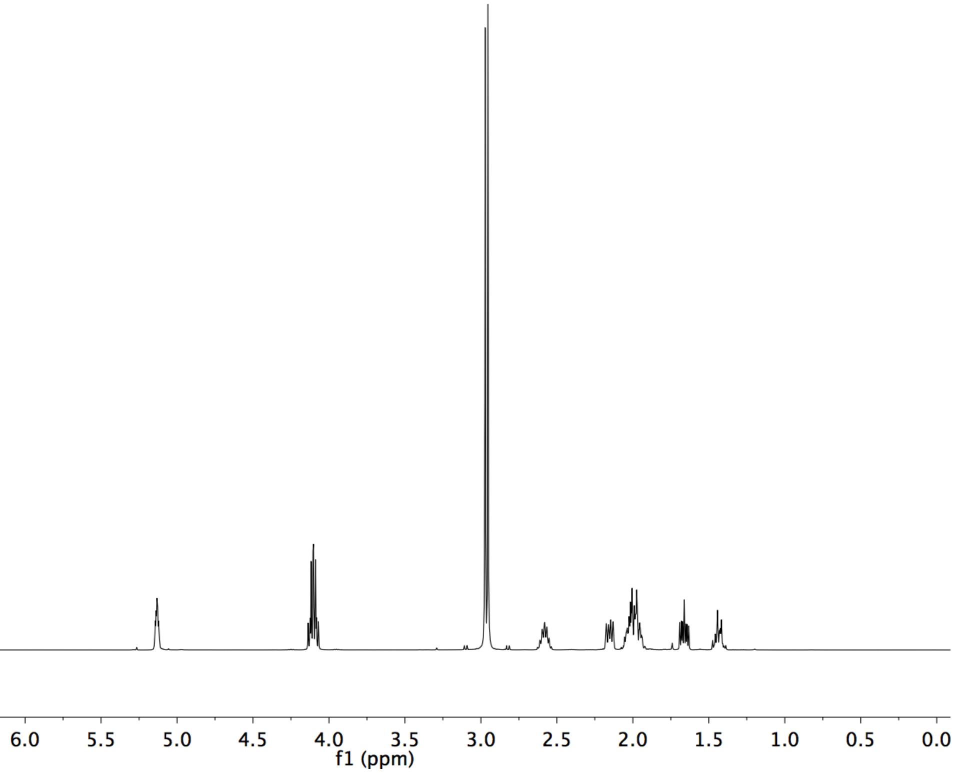

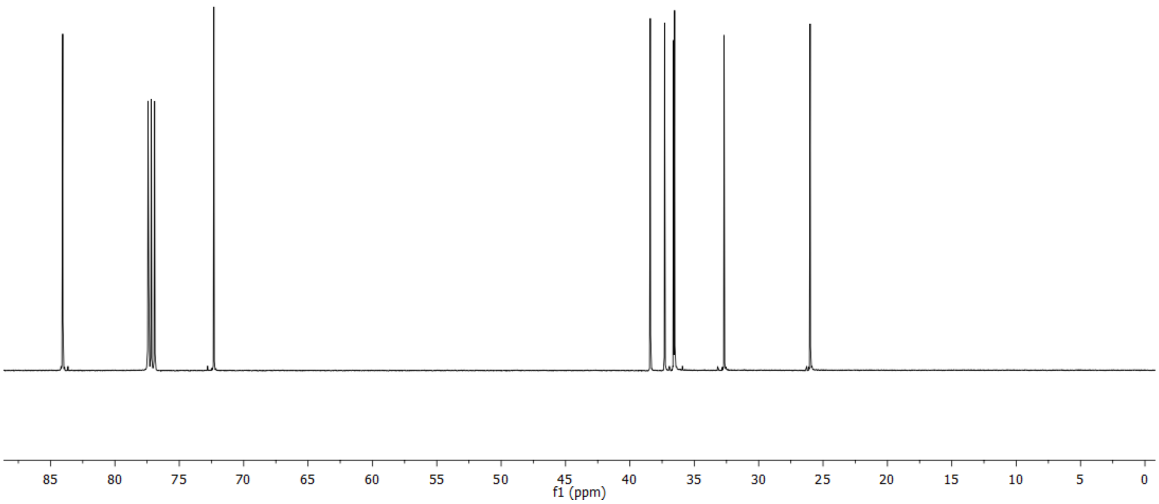

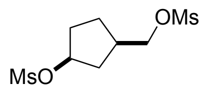

8-*cis*

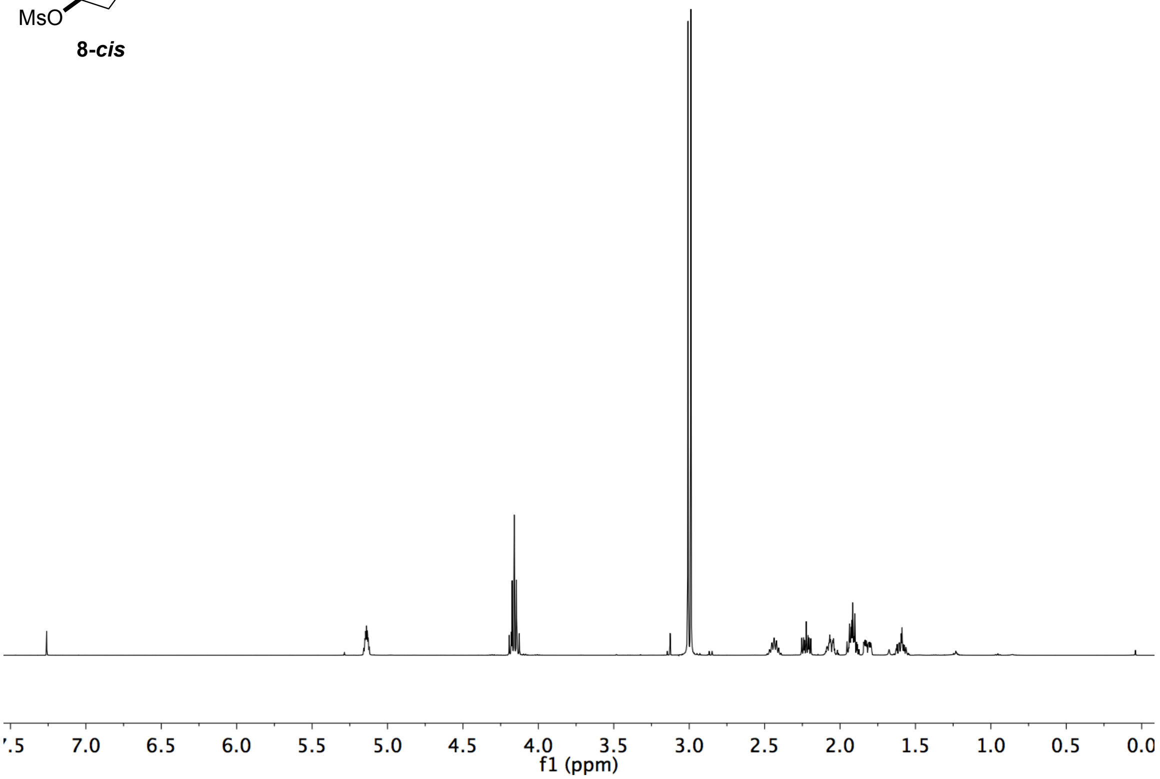

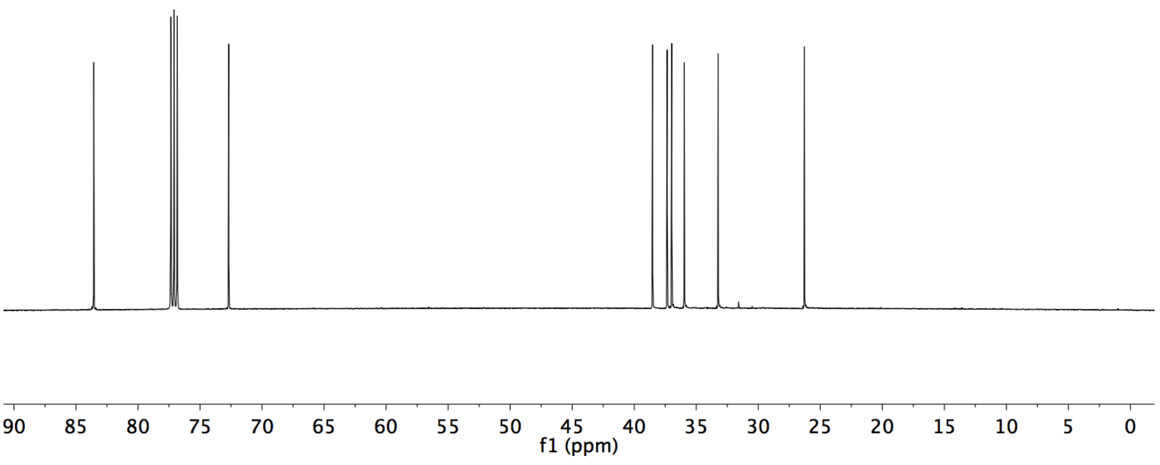

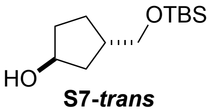
OTBS
HO

S7-*trans*

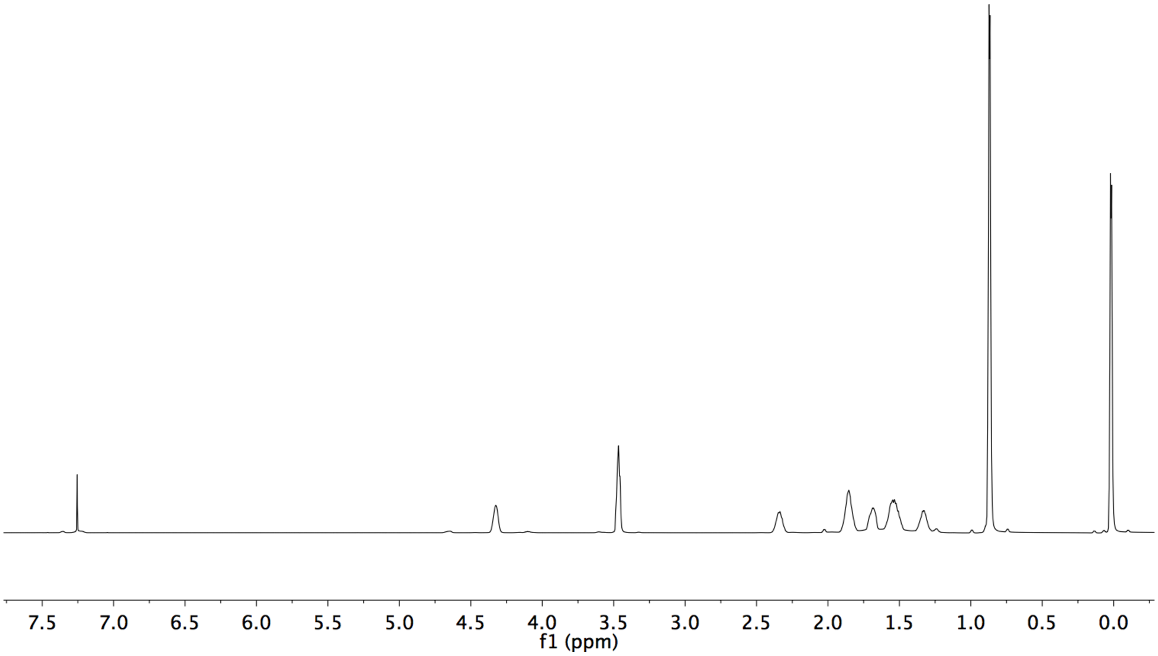
7.5 7.0 6.5 6.0 5.5 5.0 4.5 4.0 3.5 3.0 2.5 2.0 1.5 1.0 0.5 0.0
f1 (ppm)

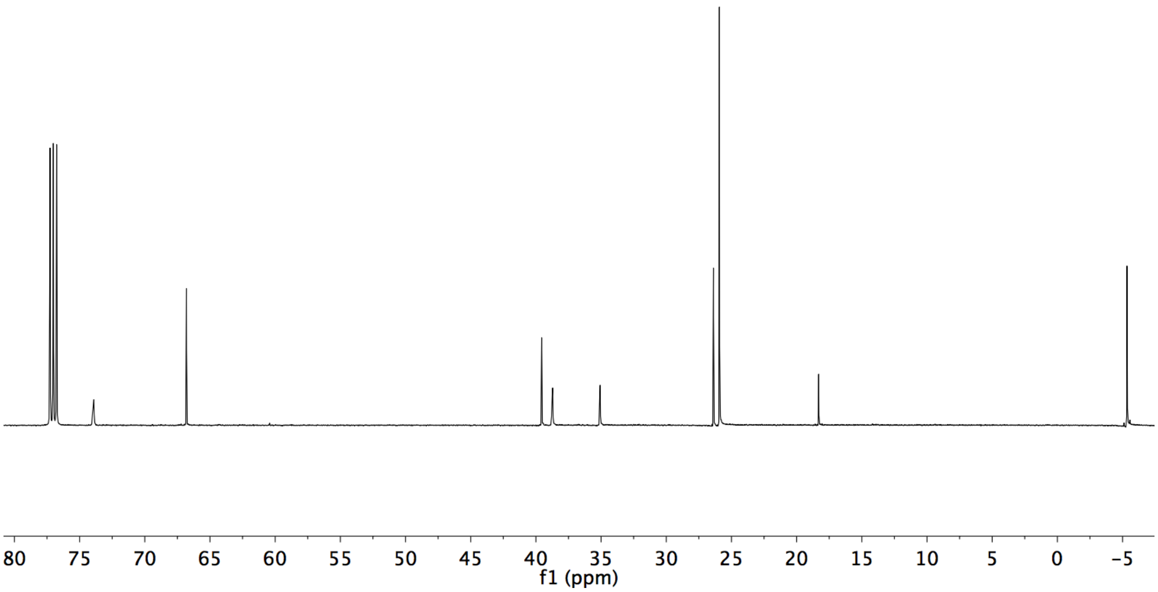
80 75 70 65 60 55 50 45 40 35 30 25 20 15 10 5 0 −5
f1 (ppm)

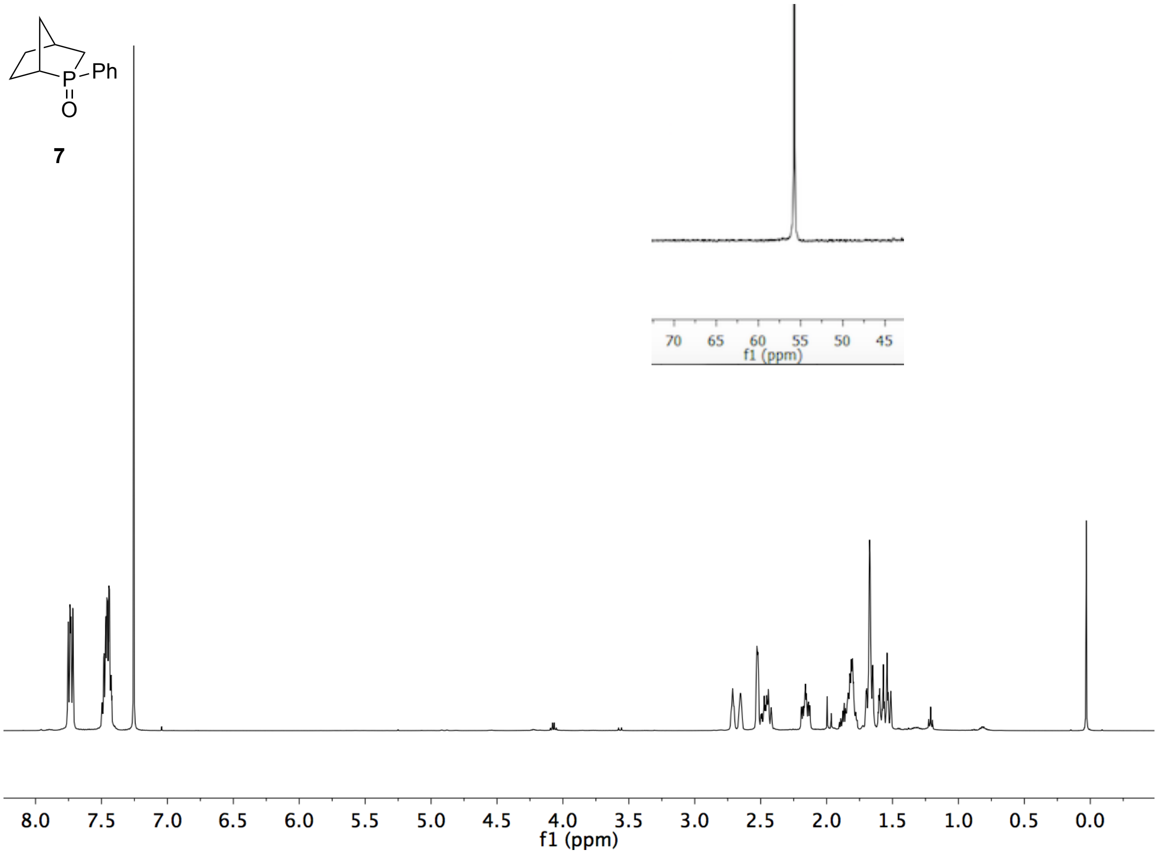
P
Ph
O
70 65 60 55 50 45
f1 (ppm)
8.0 7.5 7.0 6.5 6.0 5.5 5.0 4.5 4.0 3.5 3.0 2.5 2.0 1.5 1.0 0.5 0.0
f1 (ppm)

7

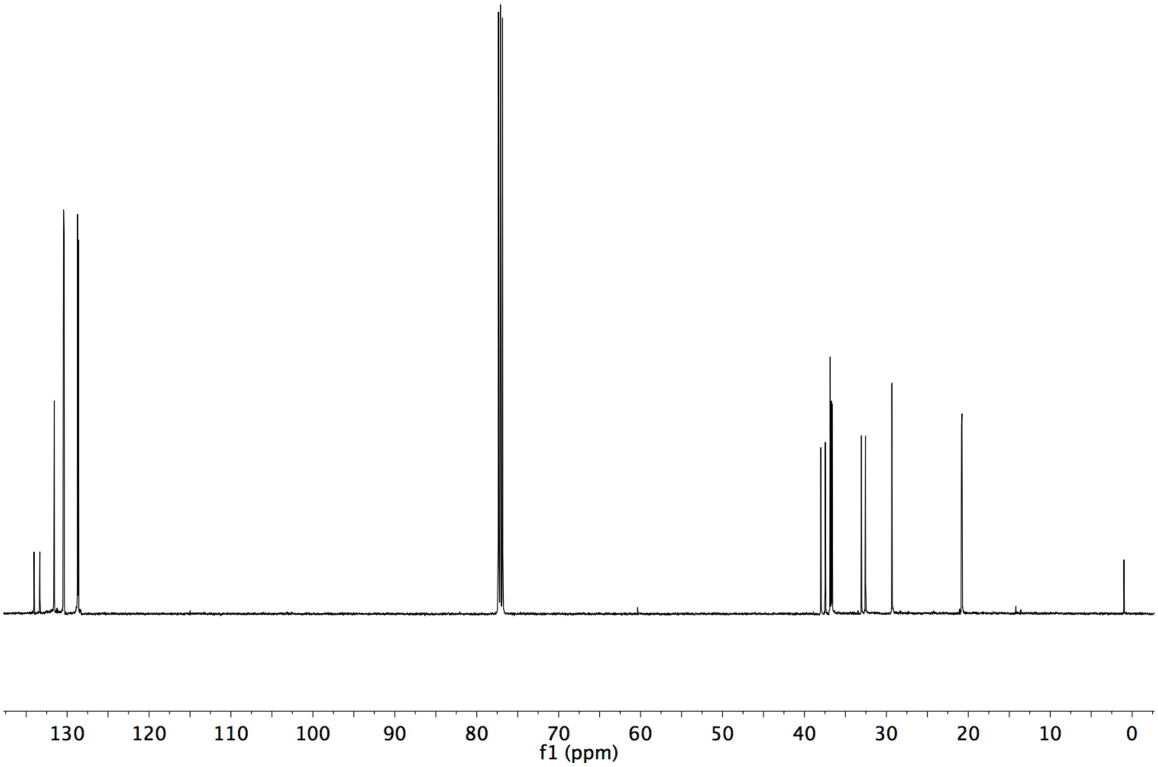
130 120 110 100 90 80 70 60 50 40 30 20 10 0
f1 (ppm)

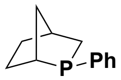
P
Ph

S3

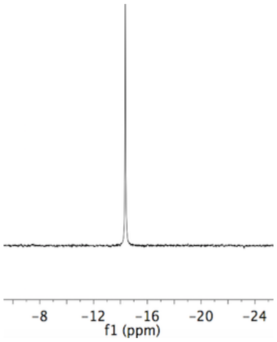
-8
-12
-16
-20
-24
f1 (ppm)

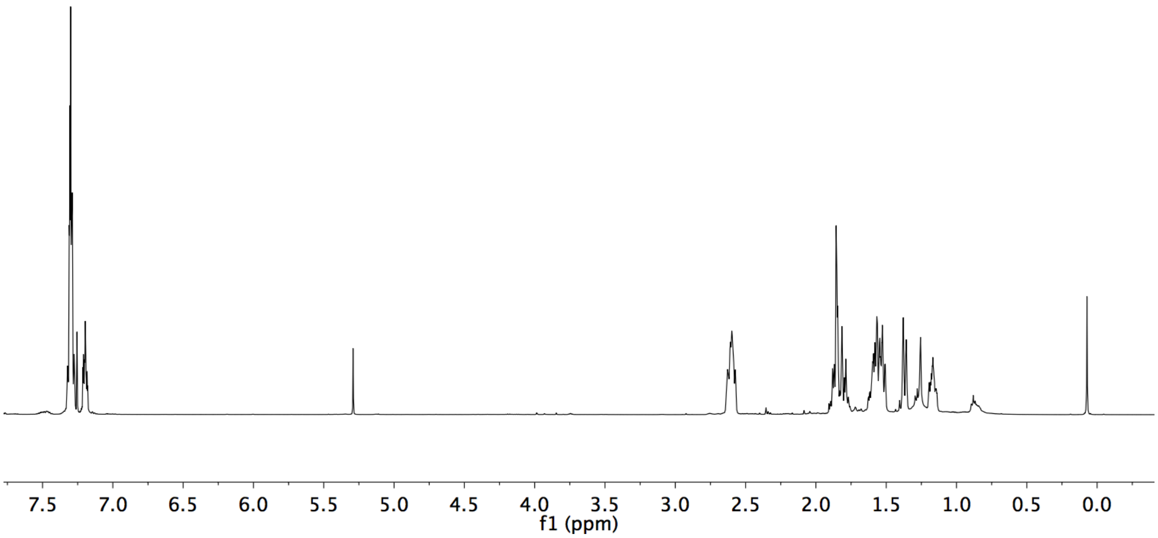
7.5 7.0 6.5 6.0 5.5 5.0 4.5 4.0 3.5 3.0 2.5 2.0 1.5 1.0 0.5 0.0
f1 (ppm)

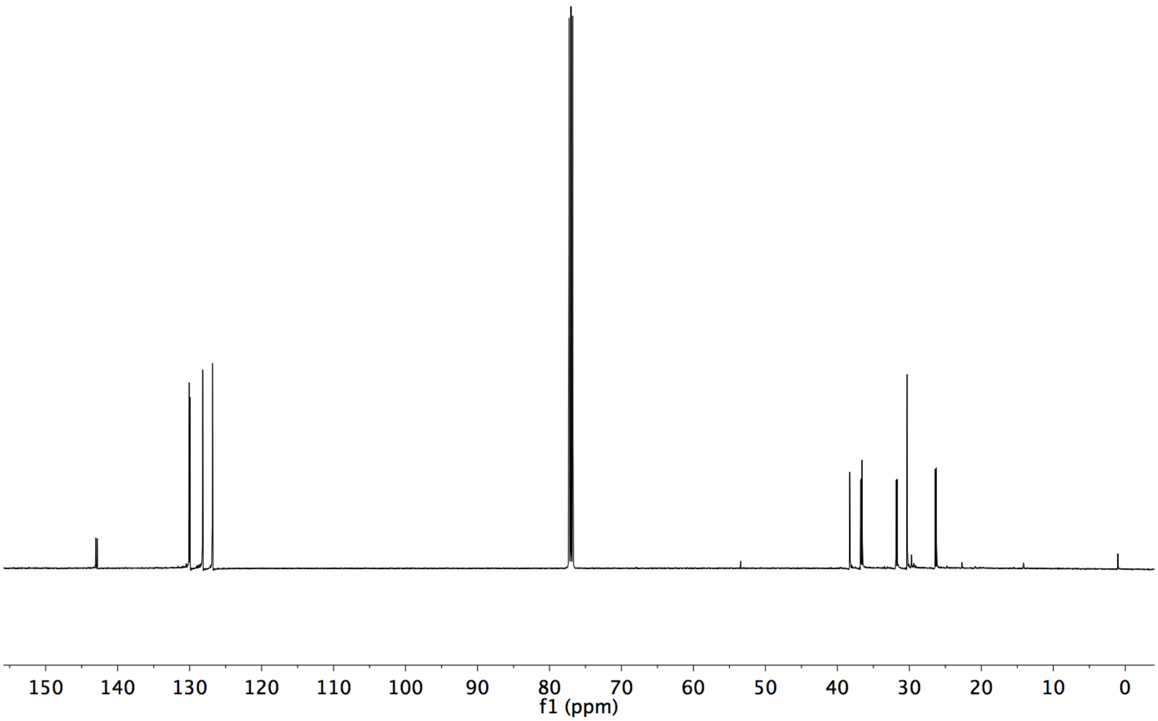
150 140 130 120 110 100 90 80 70 60 50 40 30 20 10 0
f1 (ppm)

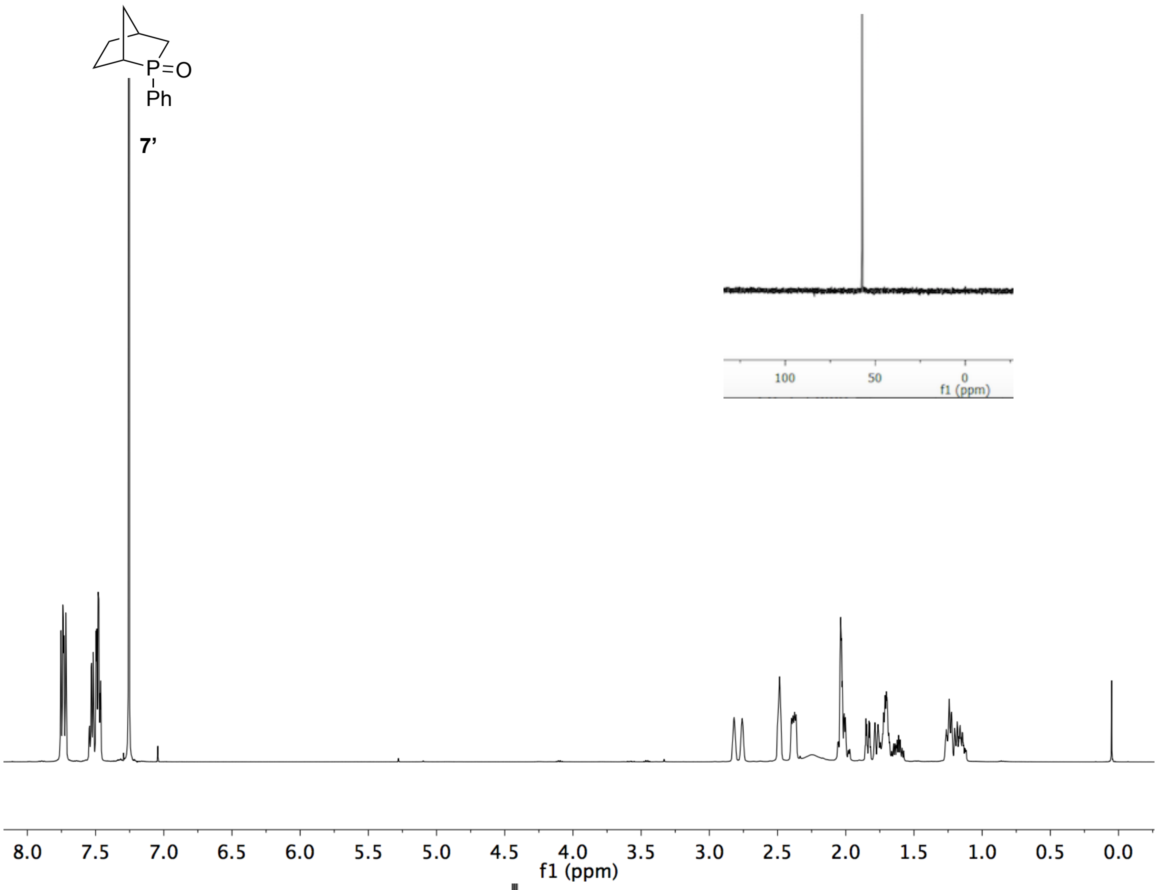
P=O
Ph
7’
f1 (ppm)
f1 (ppm)

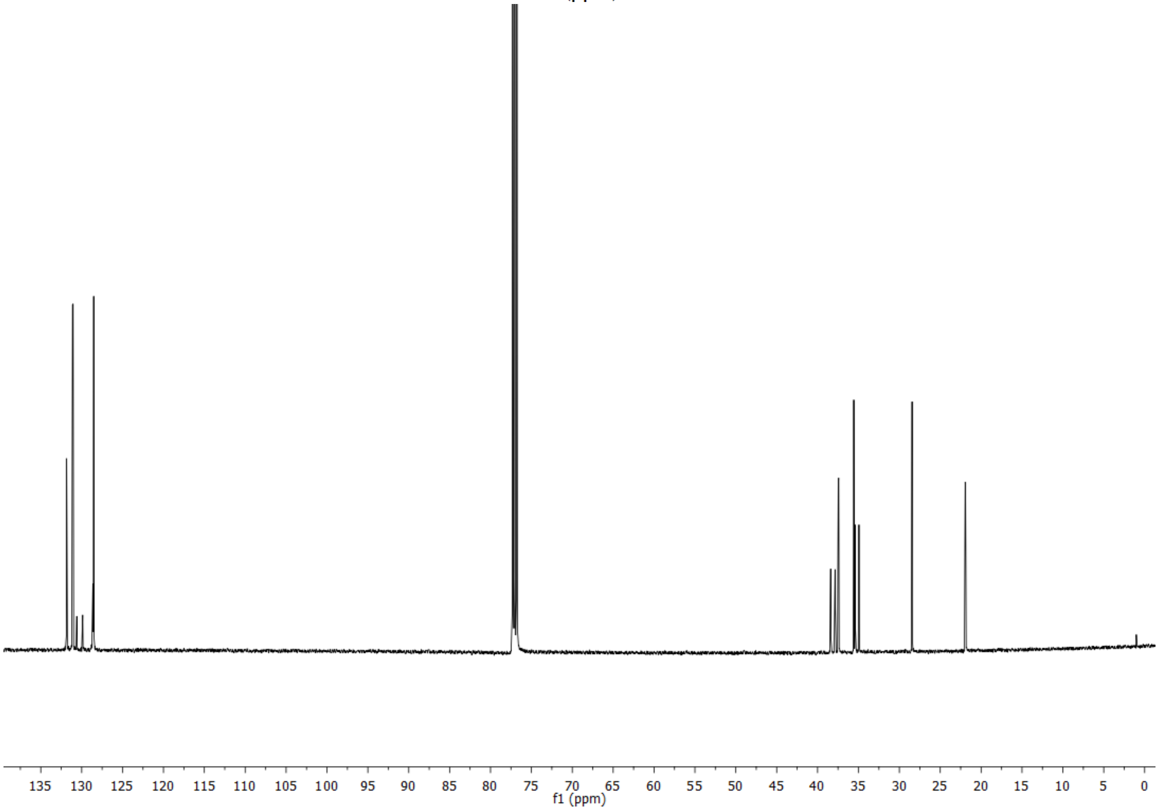
f1 (ppm)

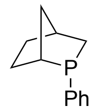
P
Ph

S4

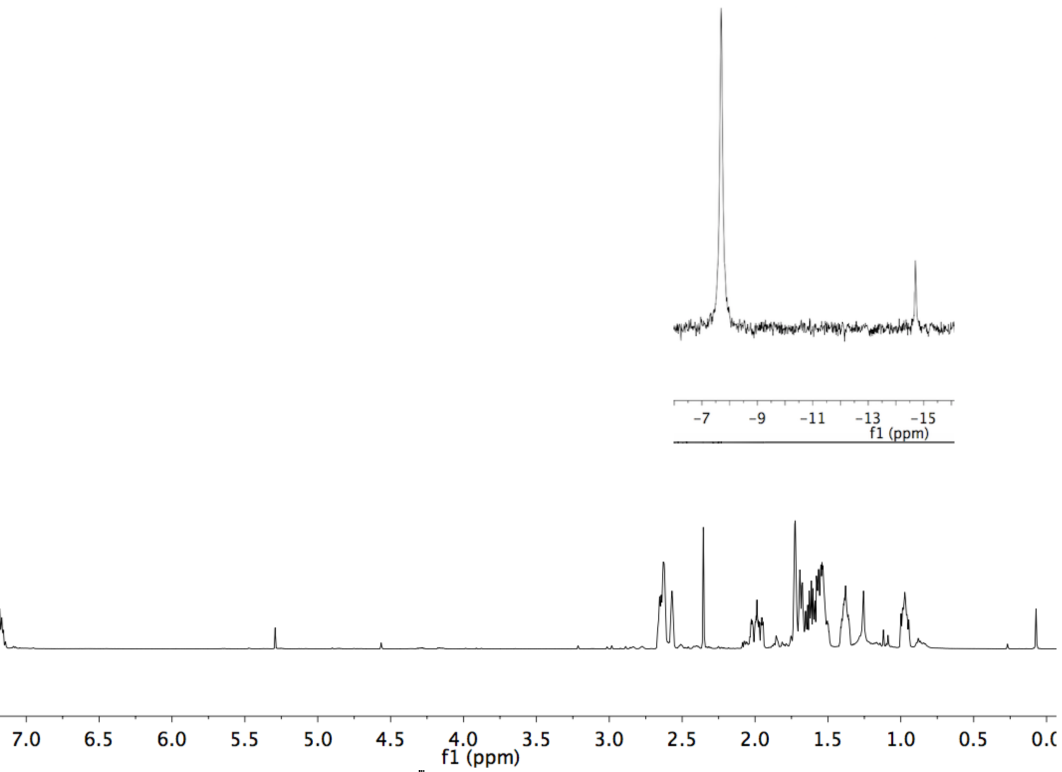
-7 -9 -11 -13 -15
f1 (ppm)
7.5 7.0 6.5 6.0 5.5 5.0 4.5 4.0 3.5 3.0 2.5 2.0 1.5 1.0 0.5 0.0
f1 (ppm)

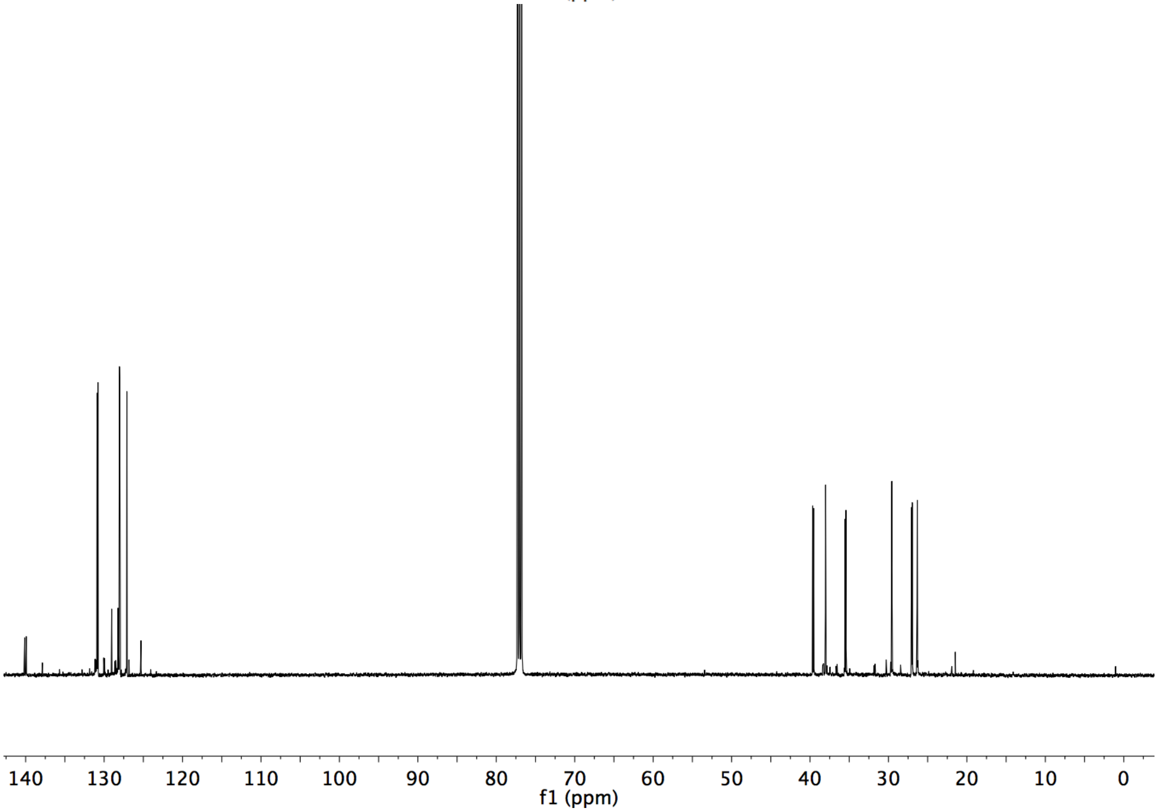
140 130 120 110 100 90 80 70 60 50 40 30 20 10 0
f1 (ppm)

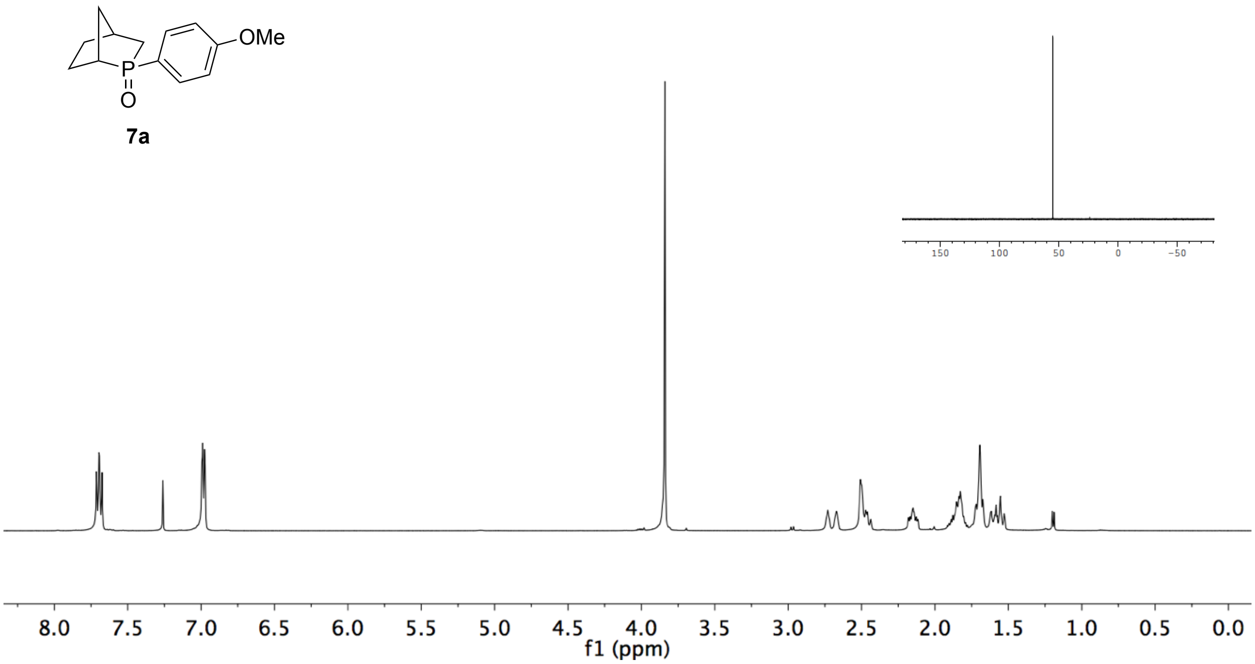
OMe
P
O
7a
150 100 50 0 −50
8.0 7.5 7.0 6.5 6.0 5.5 5.0 4.5 4.0 3.5 3.0 2.5 2.0 1.5 1.0 0.5 0.0
f1 (ppm)

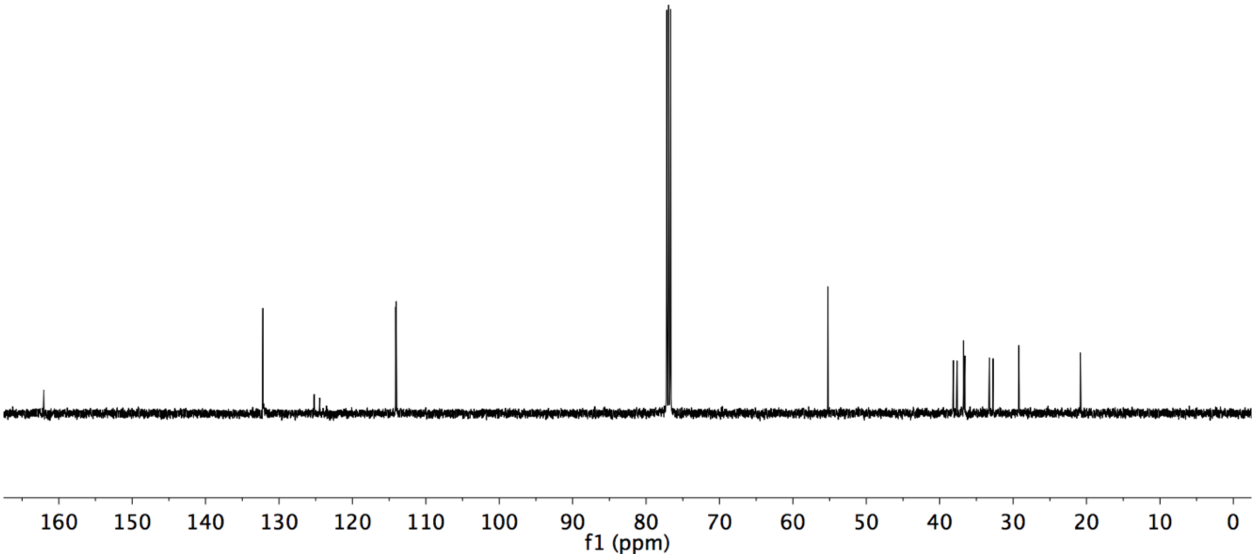
160 150 140 130 120 110 100 90 80 70 60 50 40 30 20 10 0
f1 (ppm)

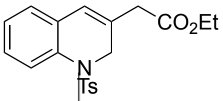
CO2Et
N
Ts

3a

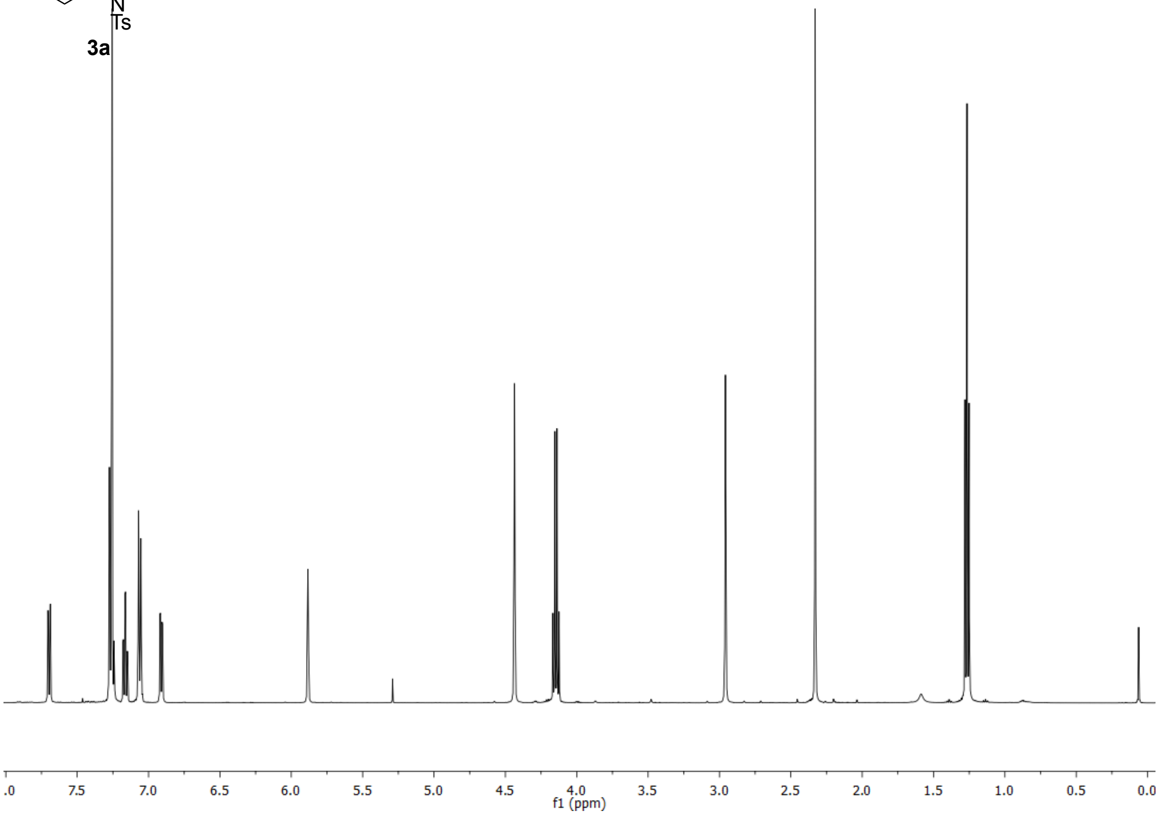
.0 7.5 7.0 6.5 6.0 5.5 5.0 4.5 4.0 3.5 3.0 2.5 2.0 1.5 1.0 0.5 0.0
f1 (ppm)

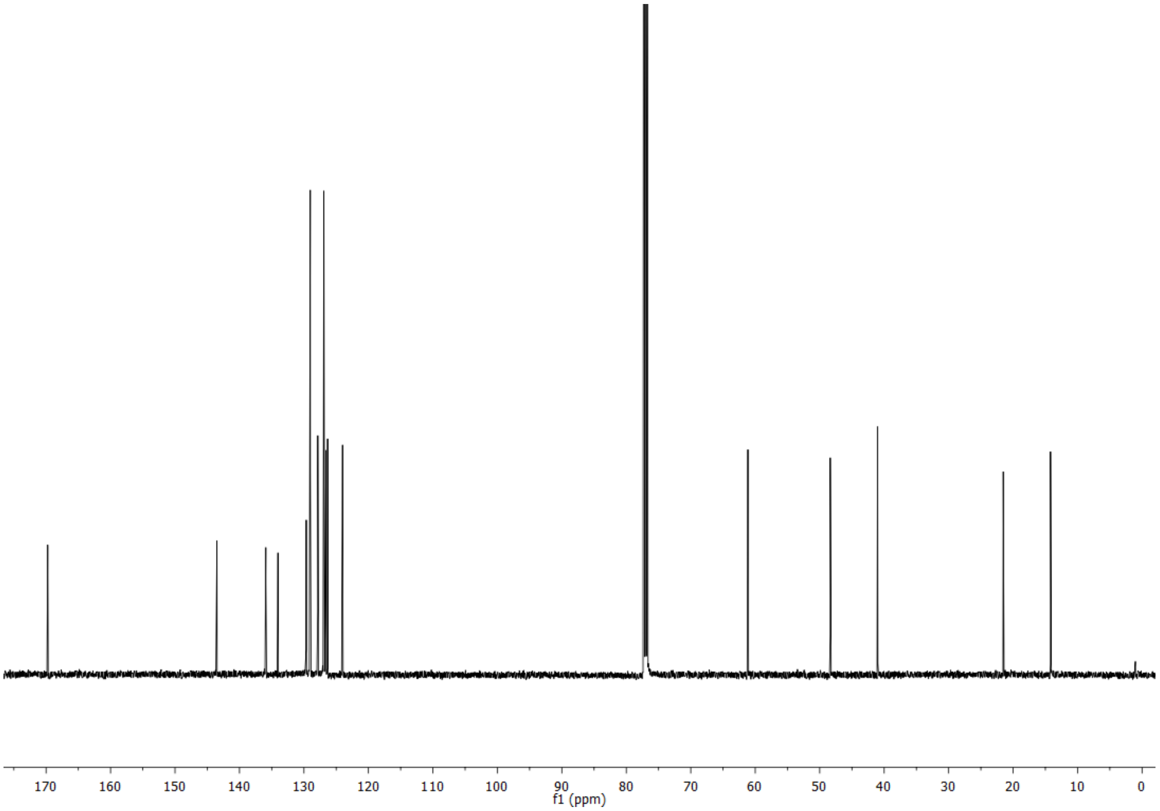
170 160 150 140 130 120 110 100 90 80 70 60 50 40 30 20 10 0
f1 (ppm)

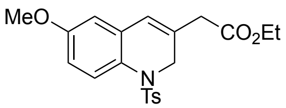
MeO
CO2Et
N
Ts

3b

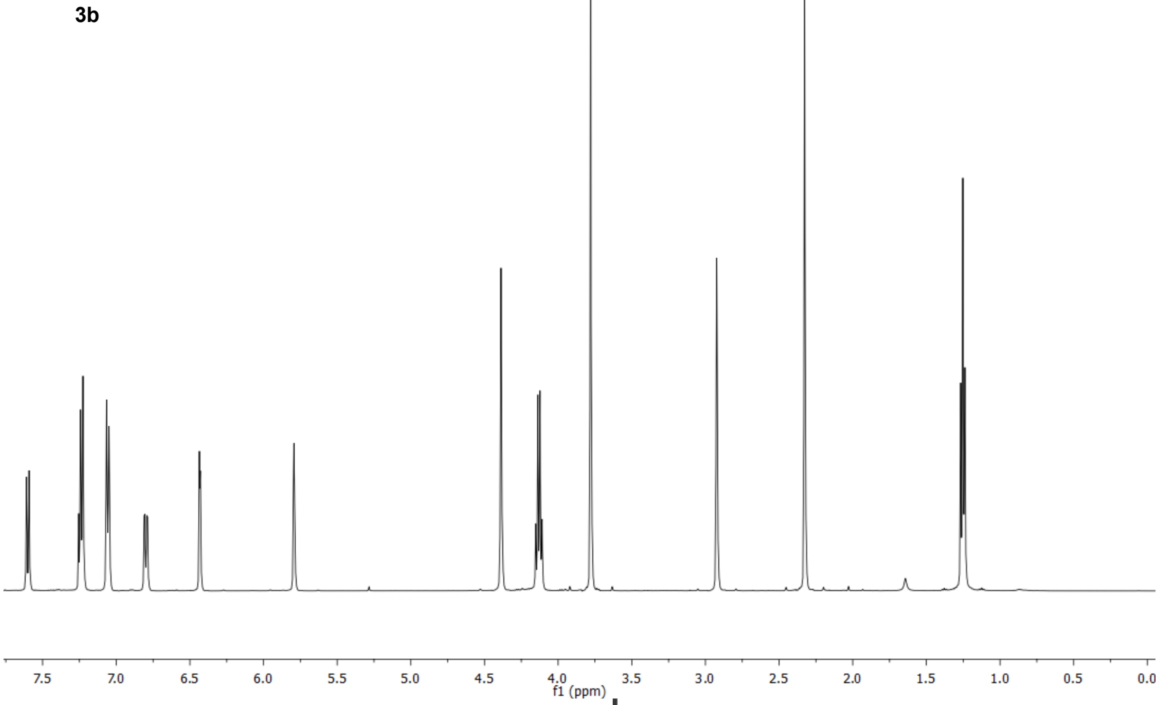
7.5 7.0 6.5 6.0 5.5 5.0 4.5 4.0 3.5 3.0 2.5 2.0 1.5 1.0 0.5 0.0
f1 (ppm)

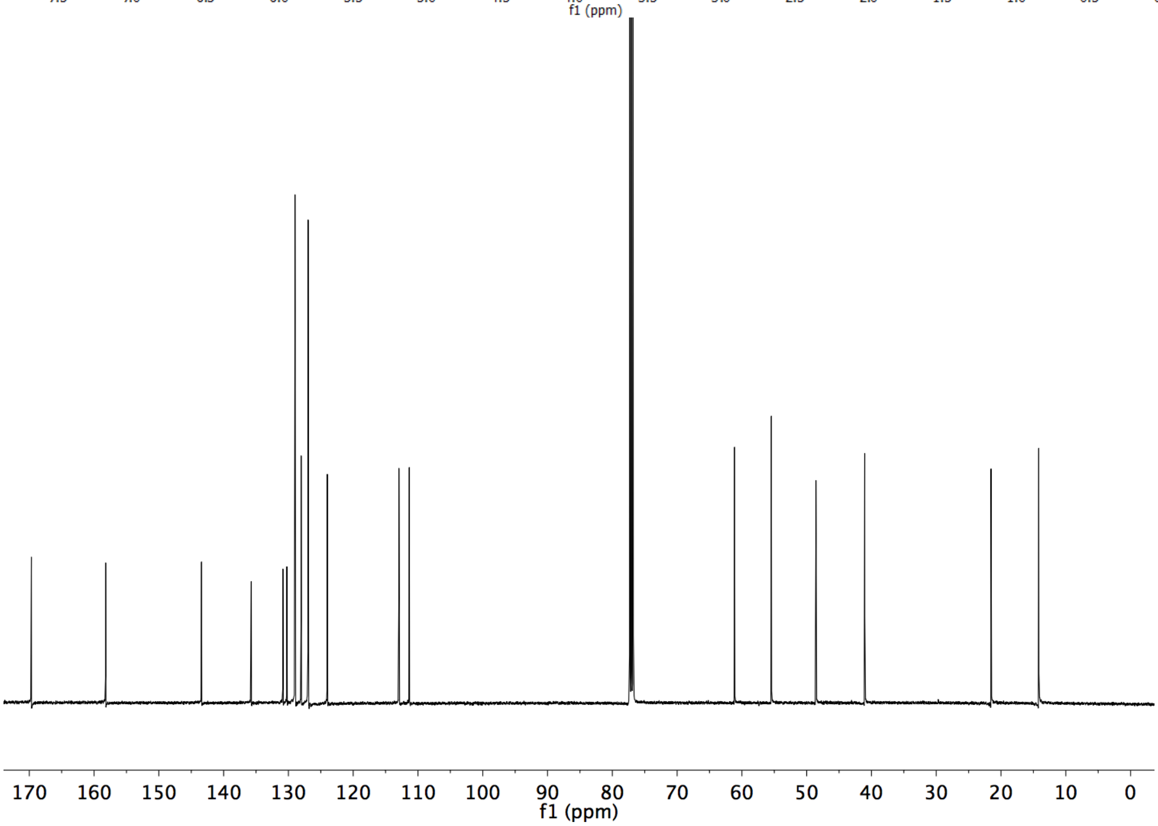
170 160 150 140 130 120 110 100 90 80 70 60 50 40 30 20 10 0
f1 (ppm)

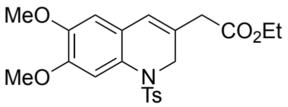

3c

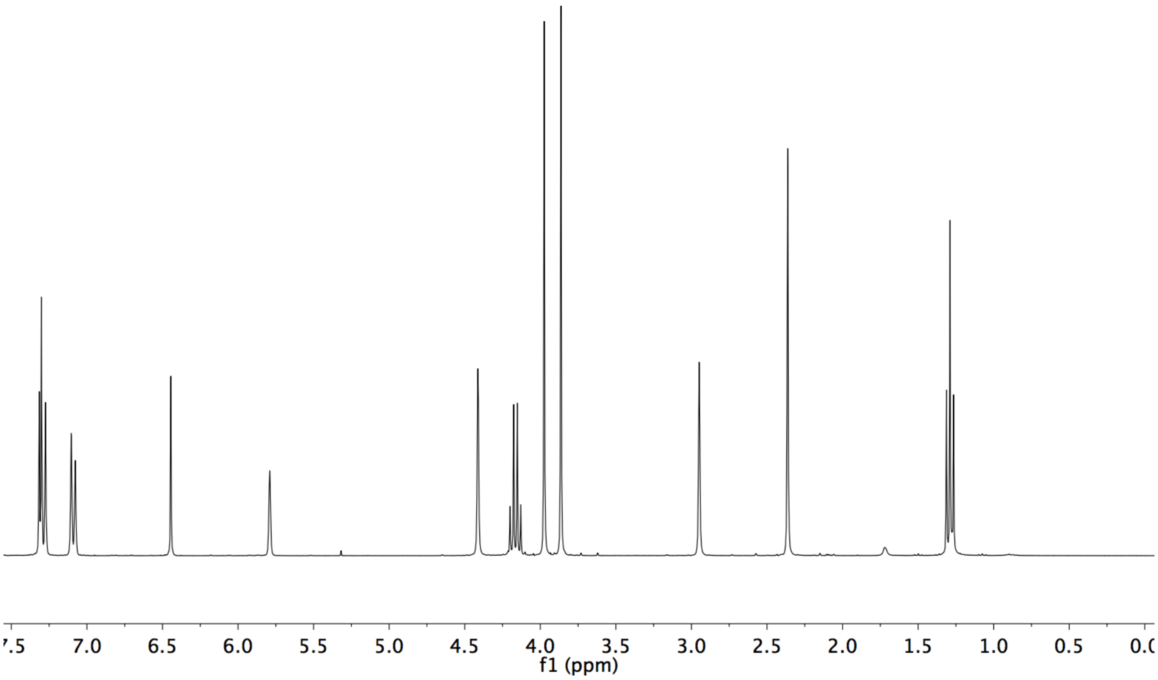

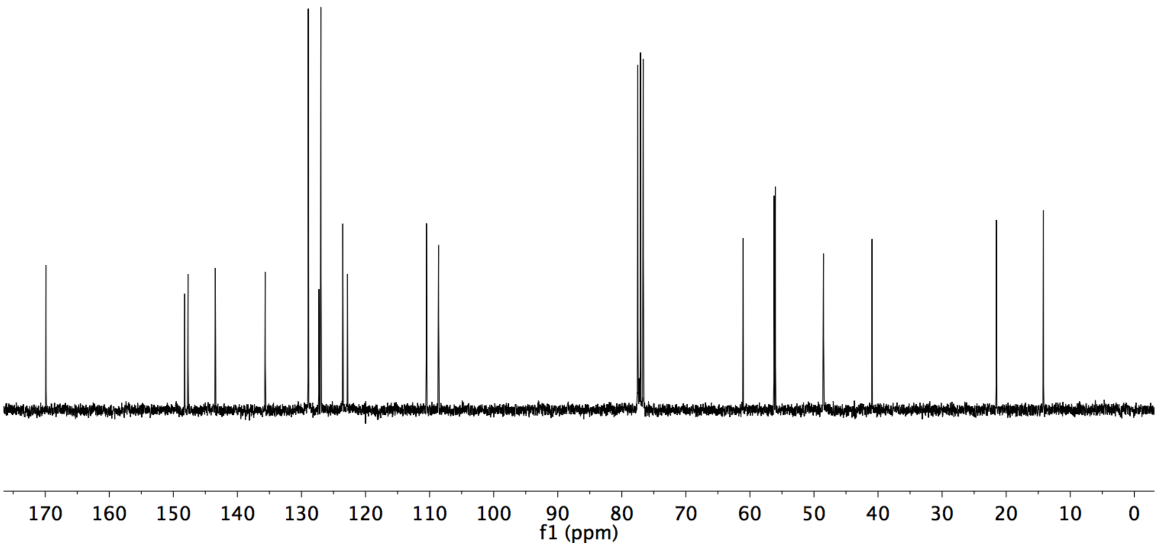

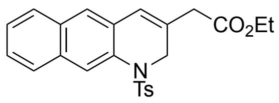
CO2Et
N
Ts

3d

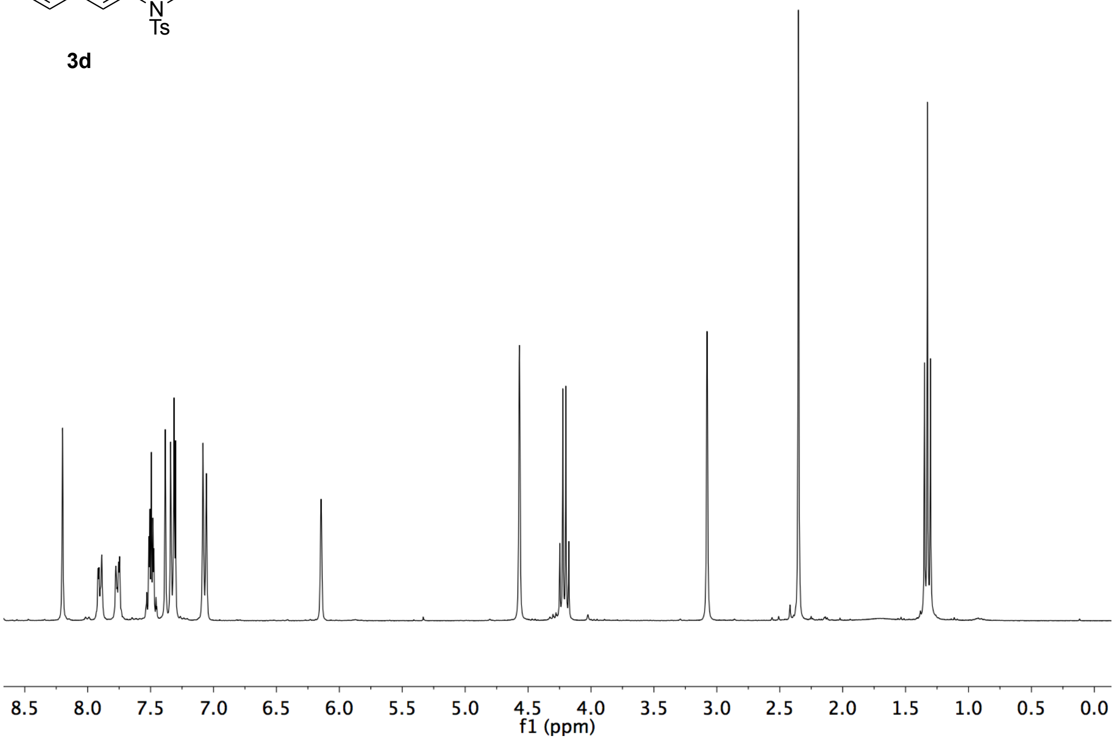
8.5 8.0 7.5 7.0 6.5 6.0 5.5 5.0 4.5 4.0 3.5 3.0 2.5 2.0 1.5 1.0 0.5 0.0
f1 (ppm)

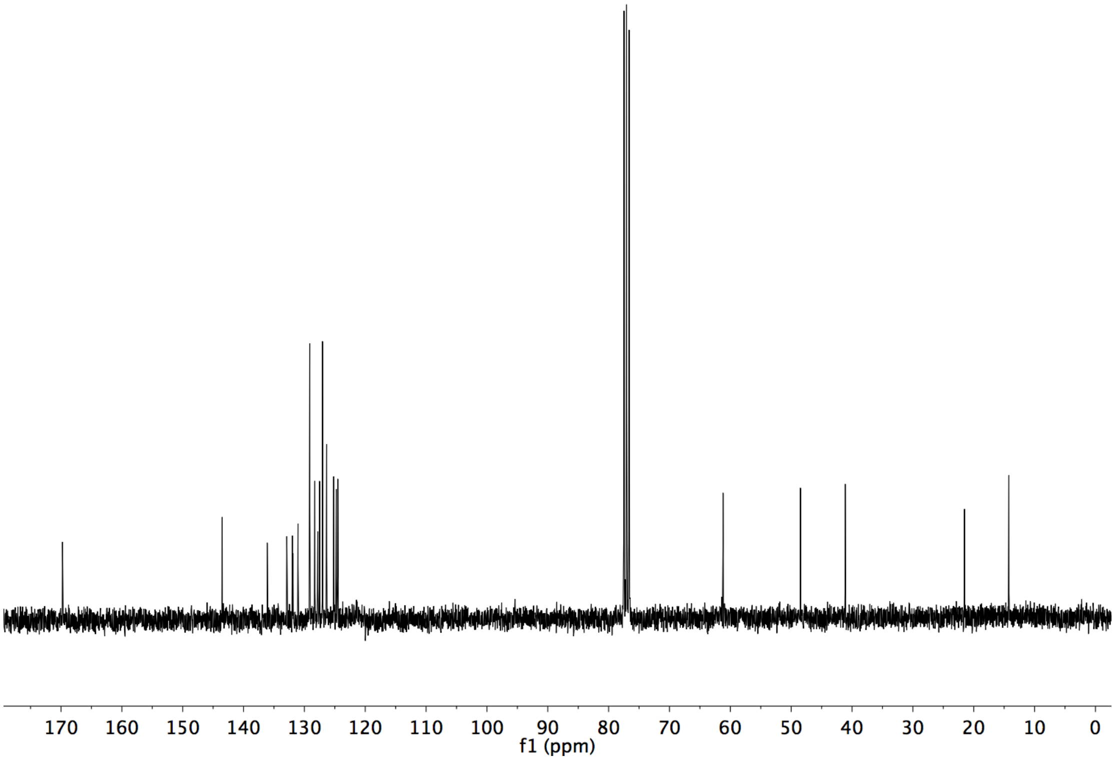
170 160 150 140 130 120 110 100 90 80 70 60 50 40 30 20 10 0
f1 (ppm)

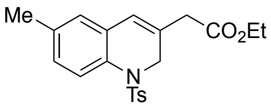
Me
CO2Et
N
Ts

3e

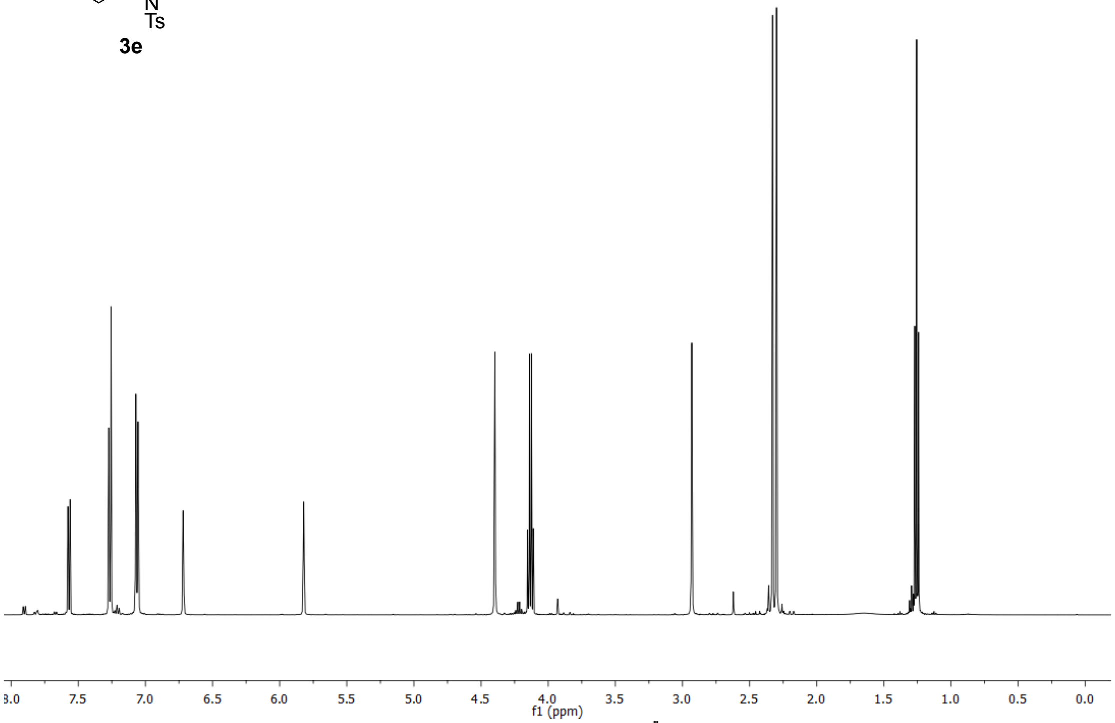
3.0 7.5 7.0 6.5 6.0 5.5 5.0 4.5 4.0 3.5 3.0 2.5 2.0 1.5 1.0 0.5 0.0
f1 (ppm)

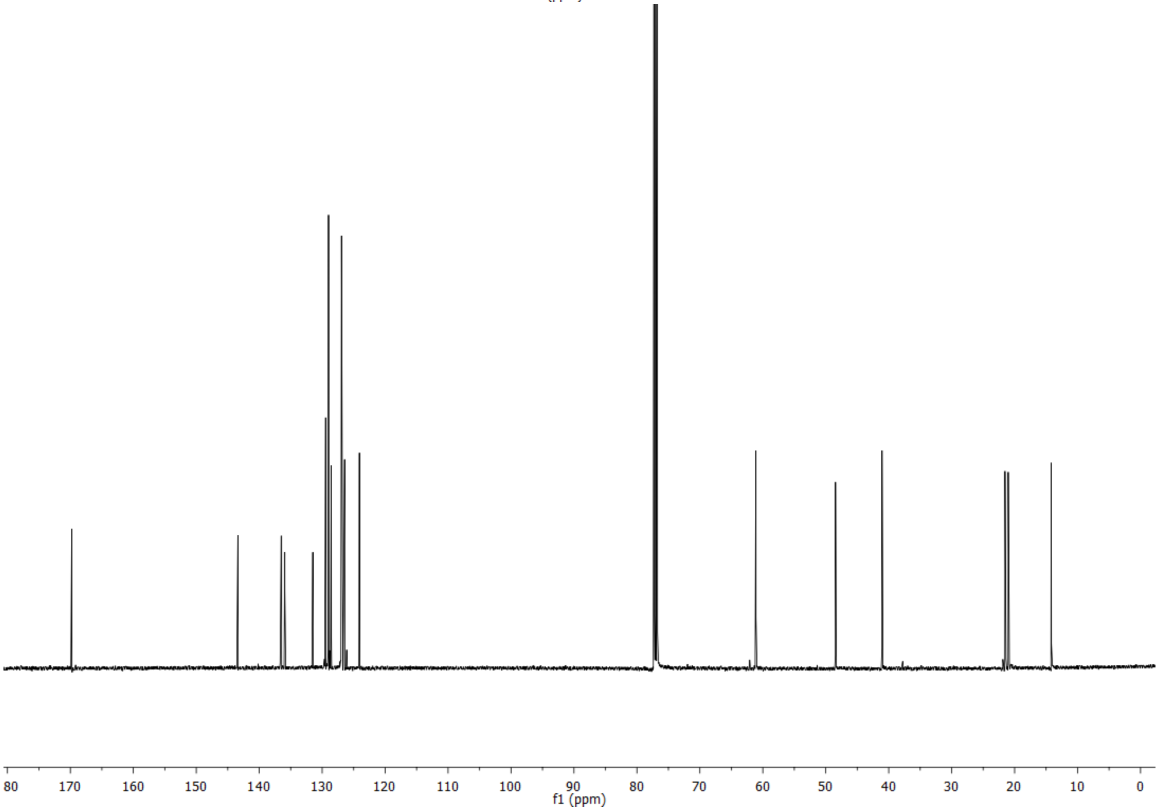
80 170 160 150 140 130 120 110 100 90 80 70 60 50 40 30 20 10 0
f1 (ppm)

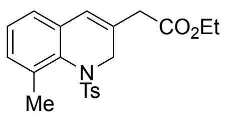
CO2Et
N
Ts
Me

3f

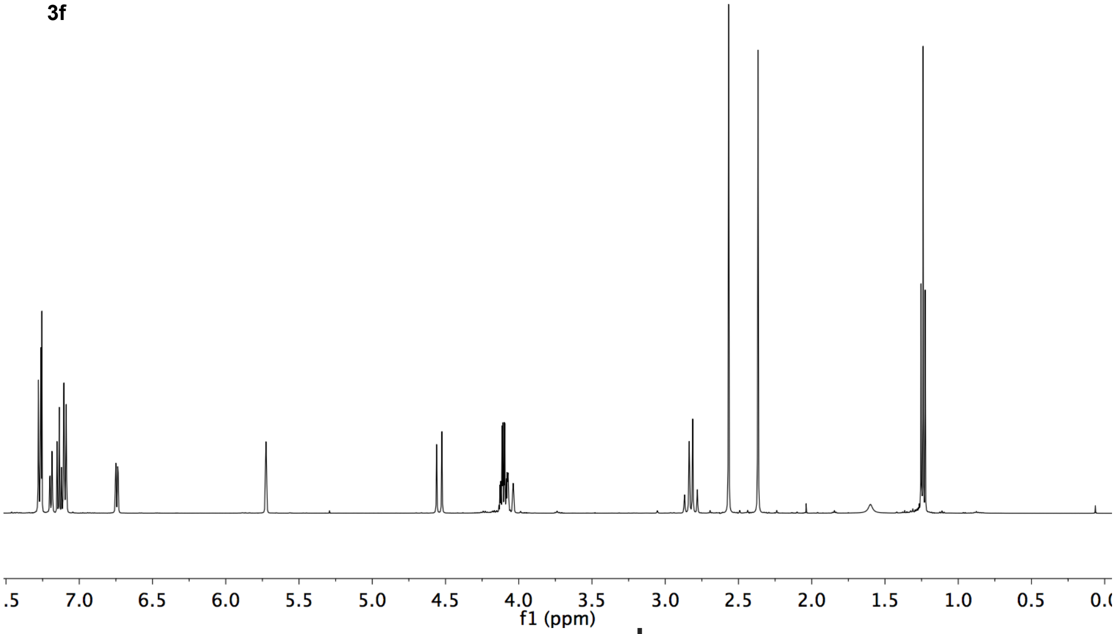
.5 7.0 6.5 6.0 5.5 5.0 4.5 4.0 3.5 3.0 2.5 2.0 1.5 1.0 0.5 0.0
f1 (ppm)

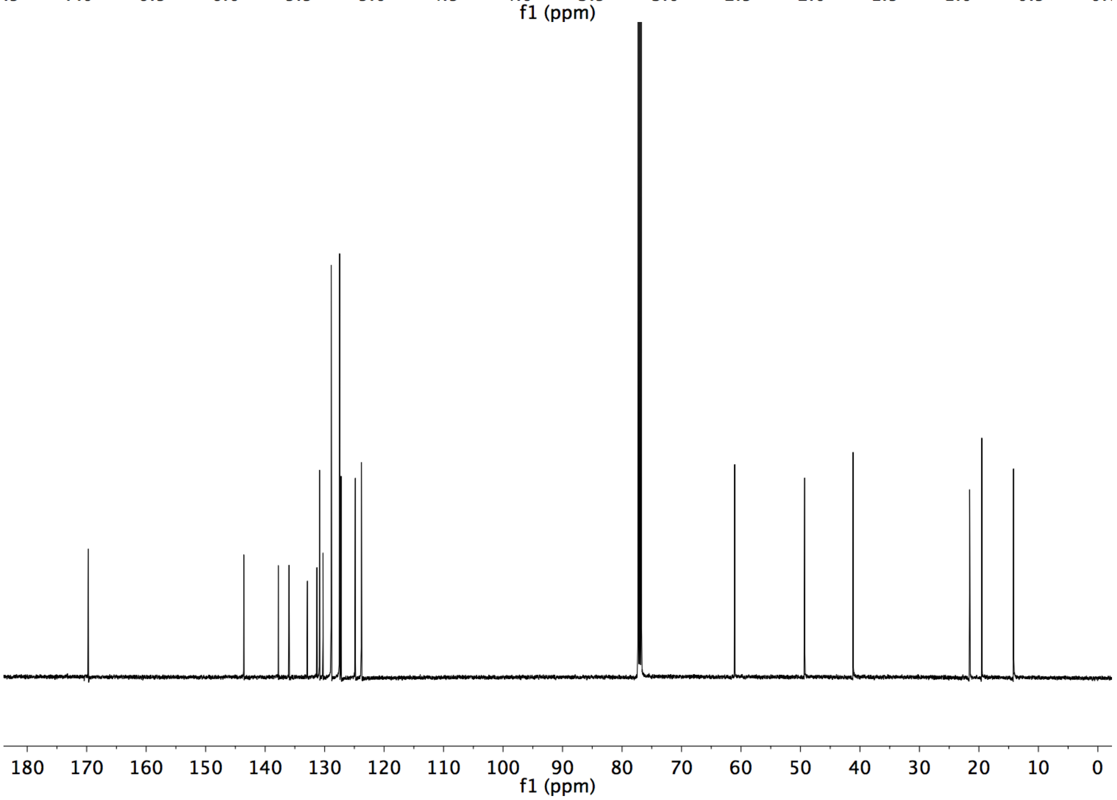
180 170 160 150 140 130 120 110 100 90 80 70 60 50 40 30 20 10 0
f1 (ppm)

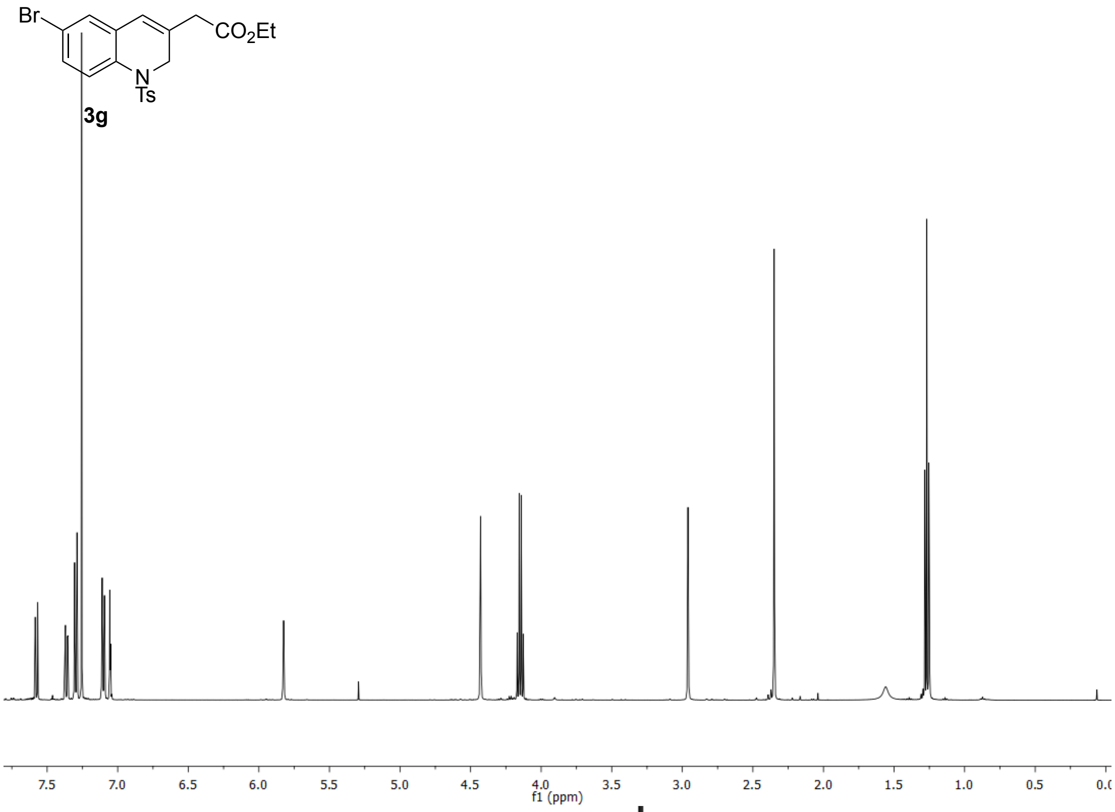
Br
CO2Et
N
Ts
3g
f1 (ppm)

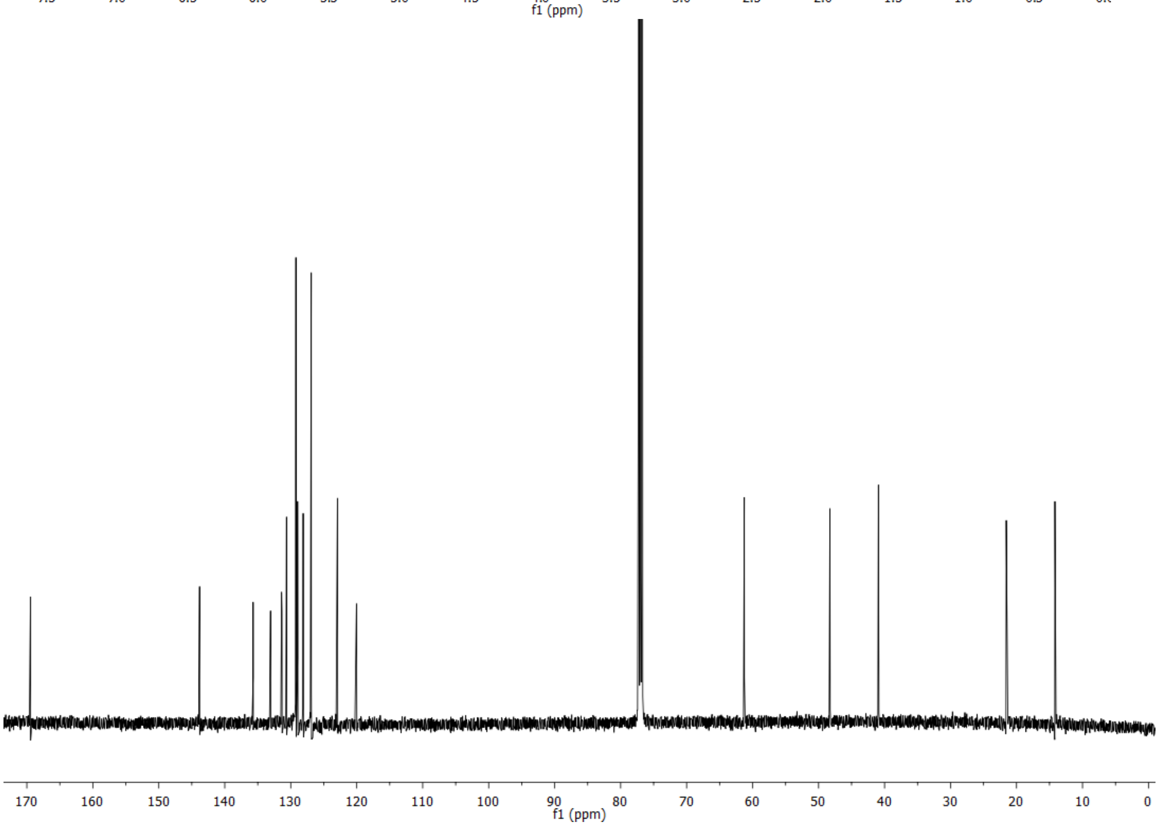
f1 (ppm)

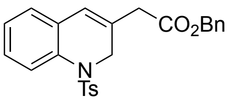
CO2Bn
N
Ts

3h

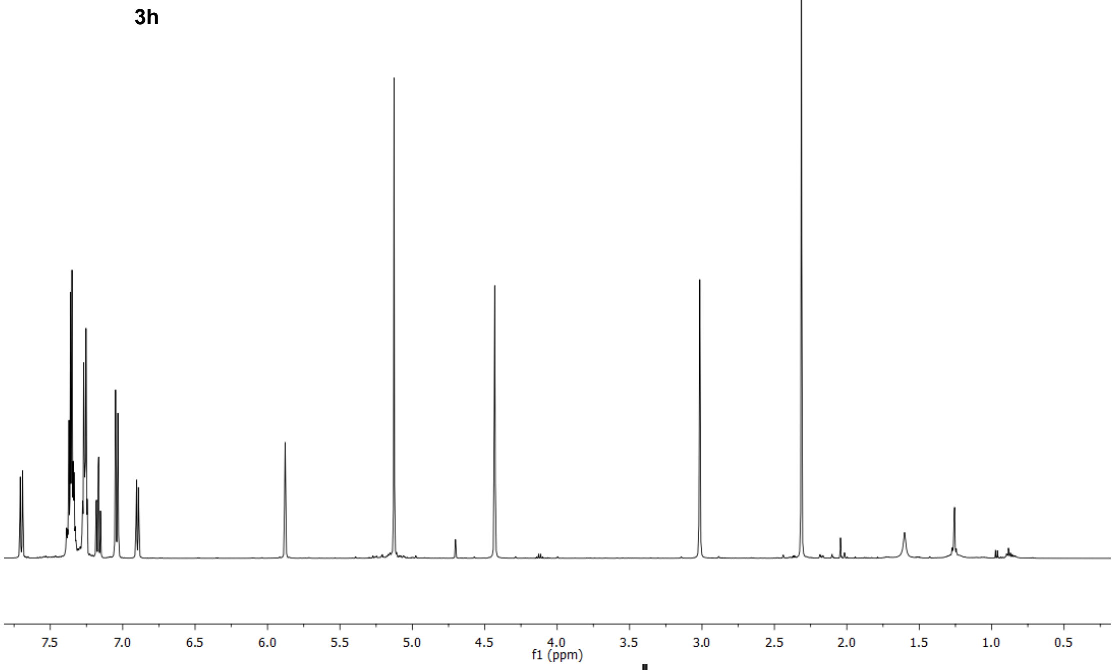
7.5
7.0
6.5
6.0
5.5
5.0
4.5
4.0
3.5
3.0
2.5
2.0
1.5
1.0
0.5
f1 (ppm)

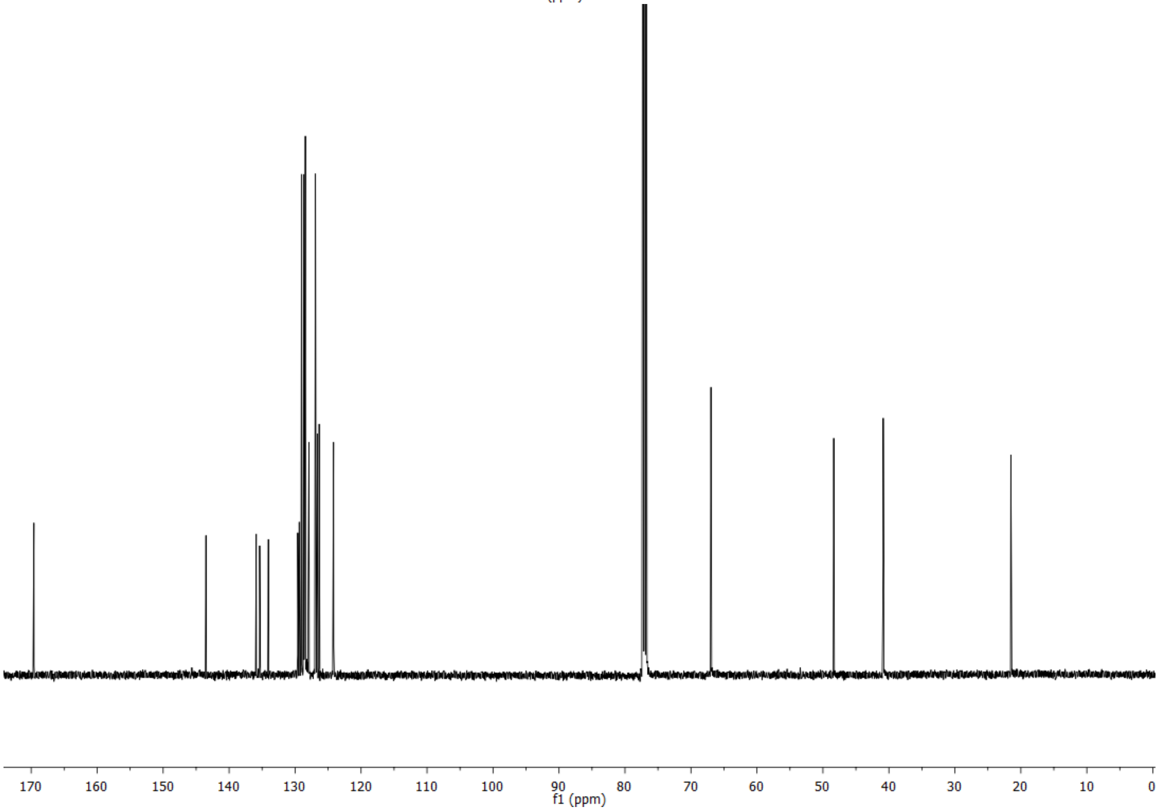
170
160
150
140
130
120
110
100
90
80
70
60
50
40
30
20
10
0
f1 (ppm)

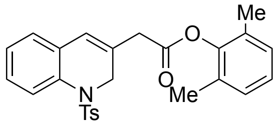

3i

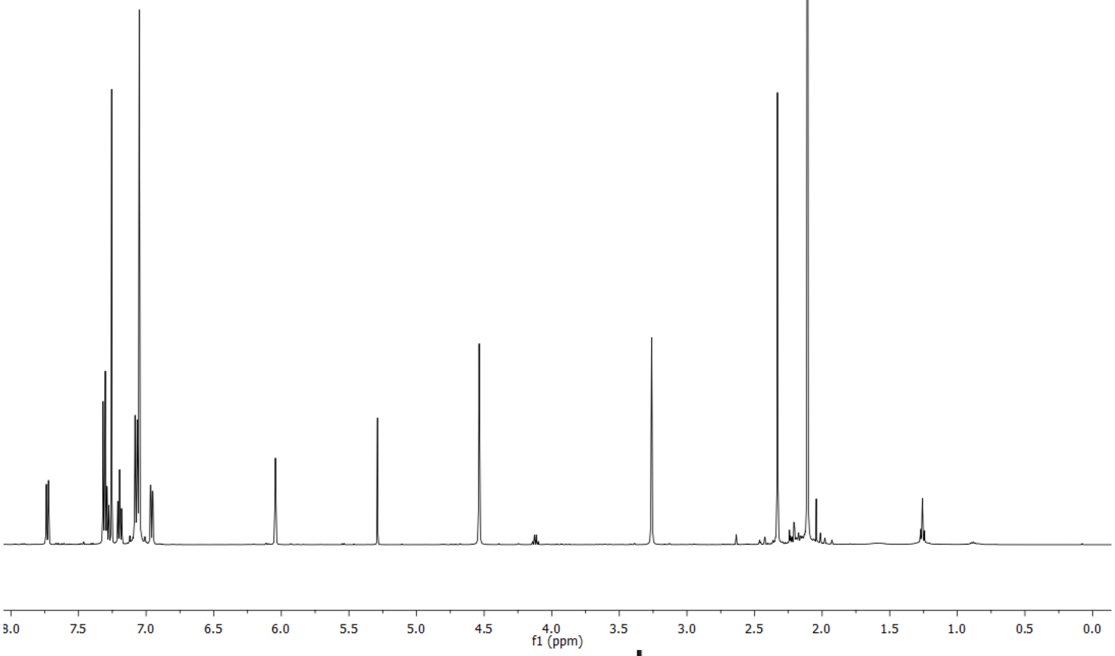

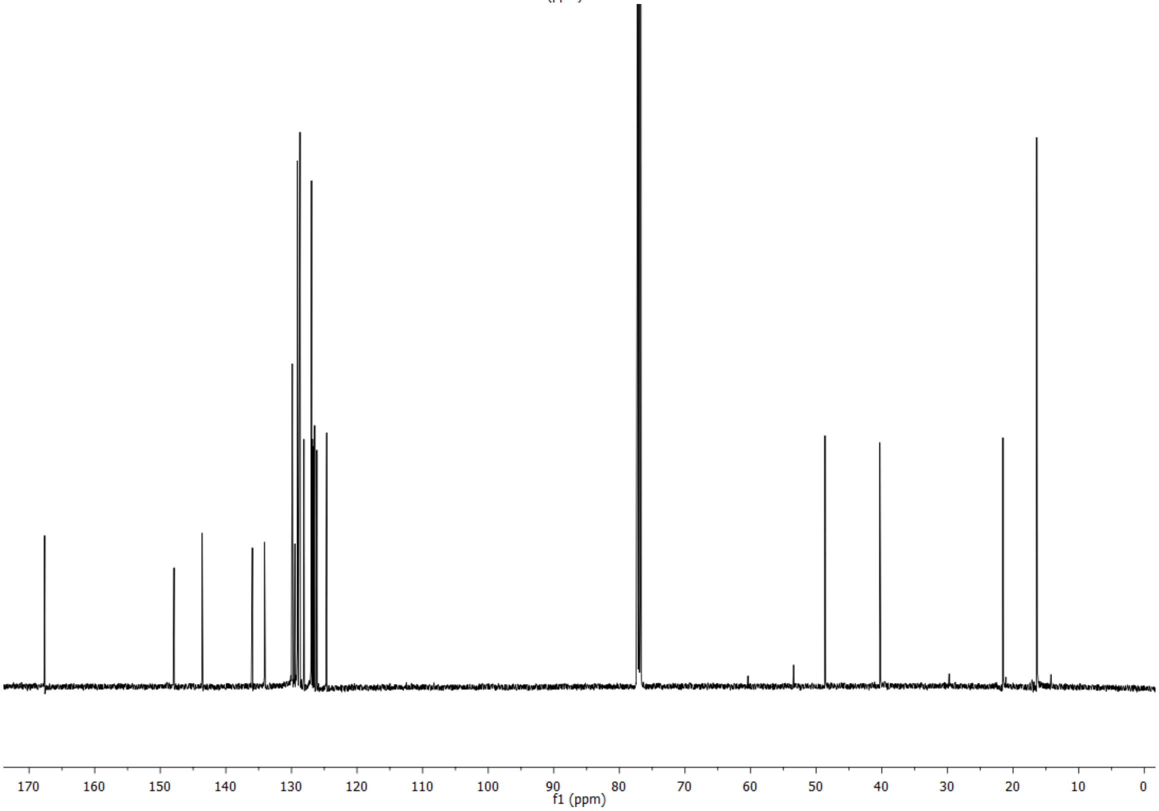

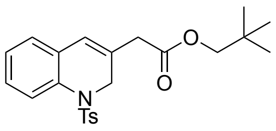

3j

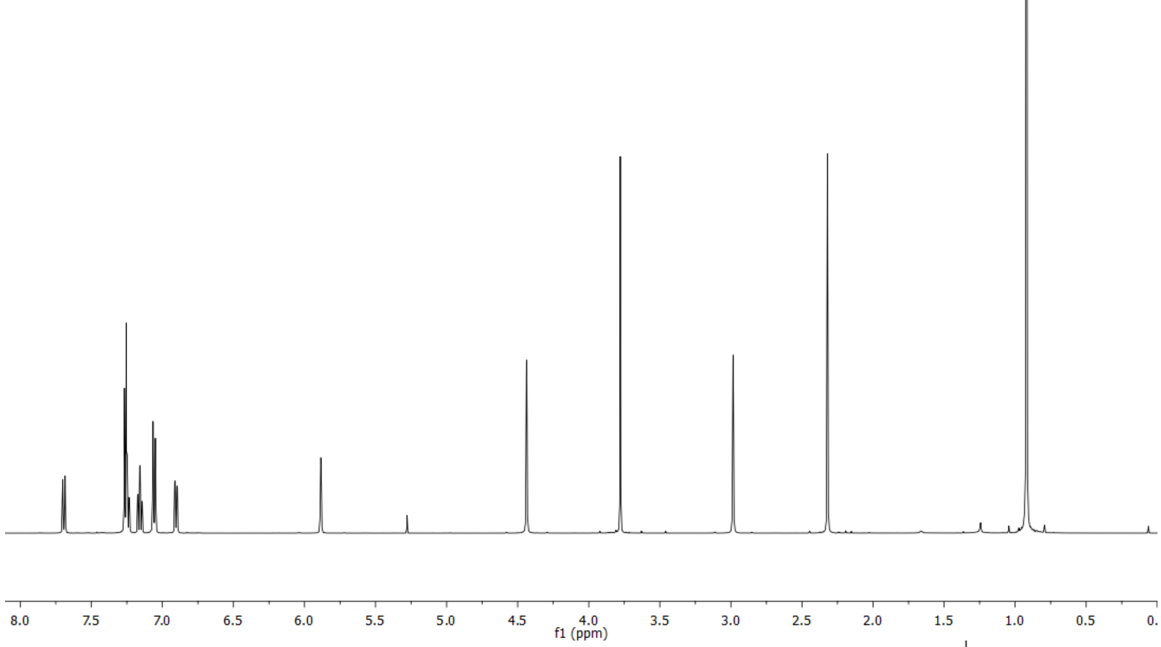

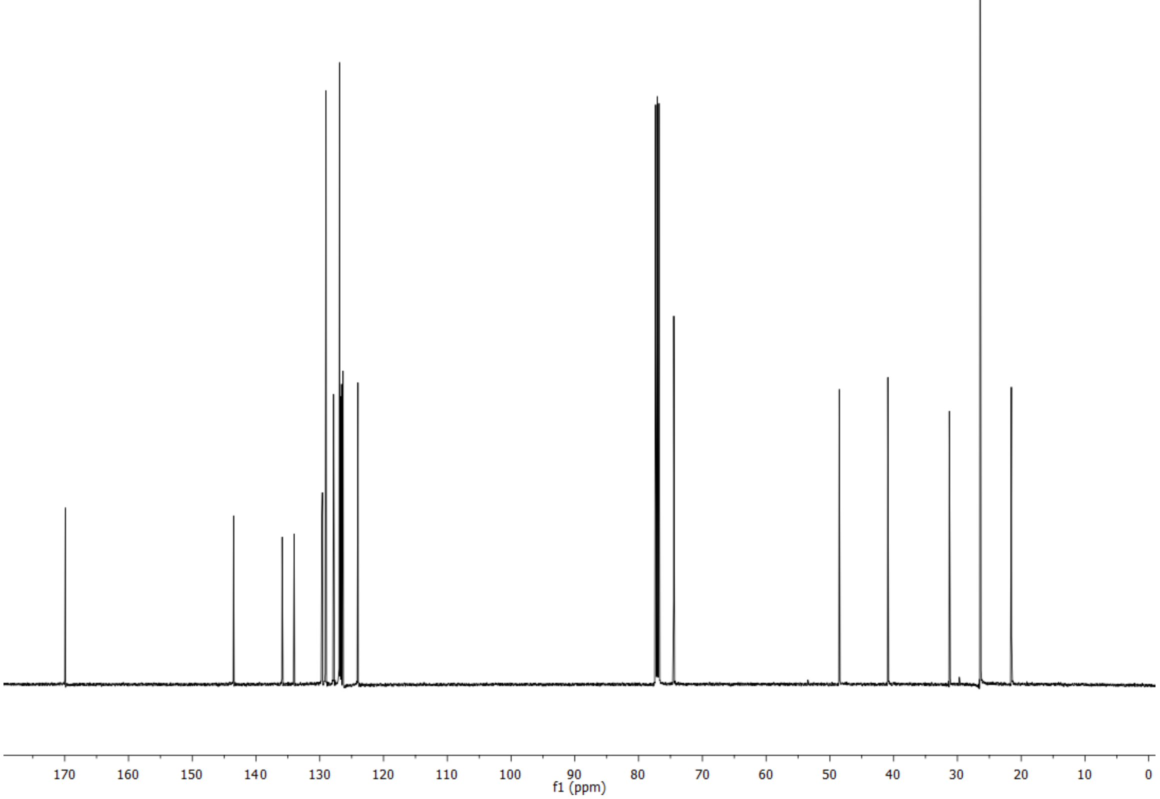

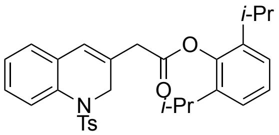

i-Pr
O
O
i-Pr
N
Ts

3k

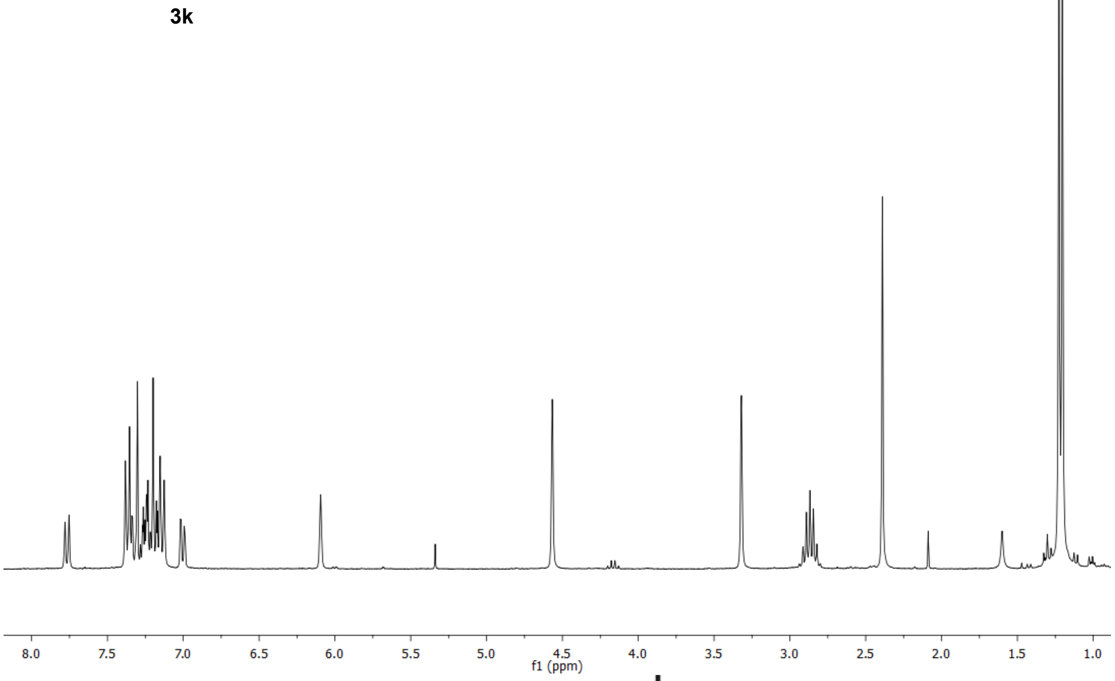

8.0
7.5
7.0
6.5
6.0
5.5
5.0
4.5
4.0
3.5
3.0
2.5
2.0
1.5
1.0
f1 (ppm)

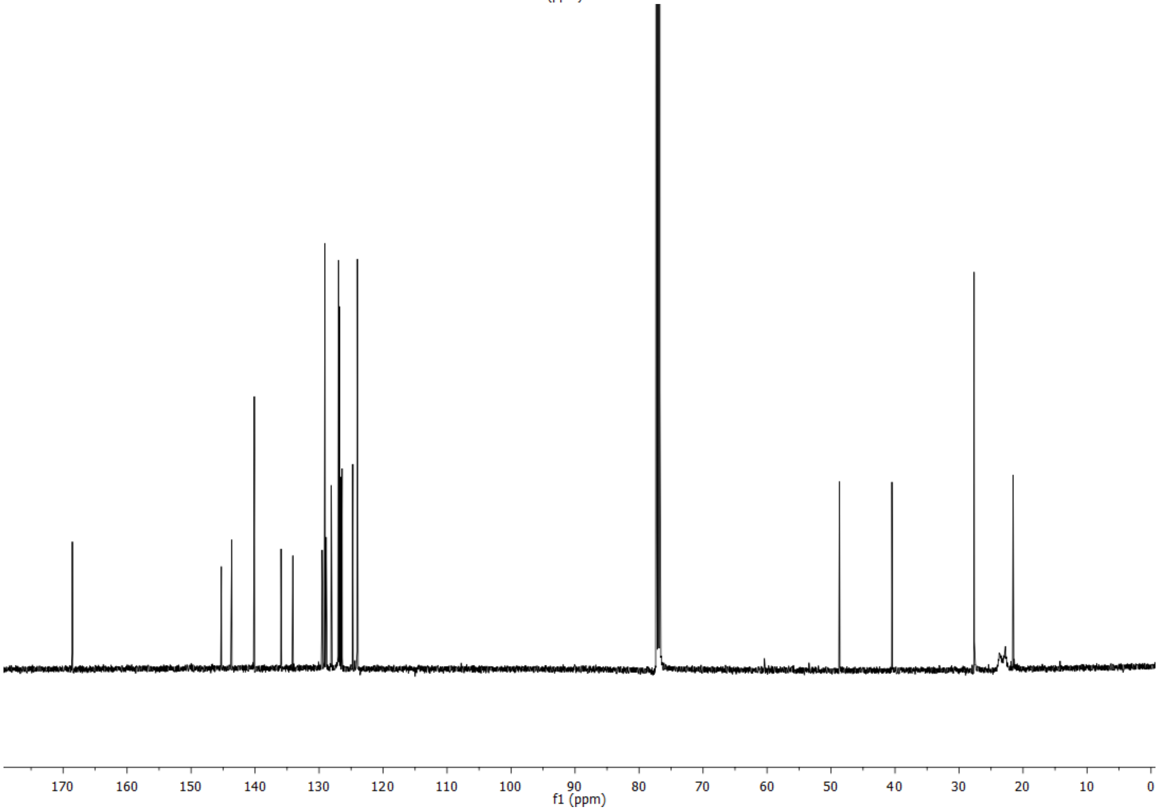

170
160
150
140
130
120
110
100
90
80
70
60
50
40
30
20
10
0
f1 (ppm)

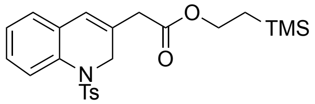
O
TMS
O
N
Ts

3l

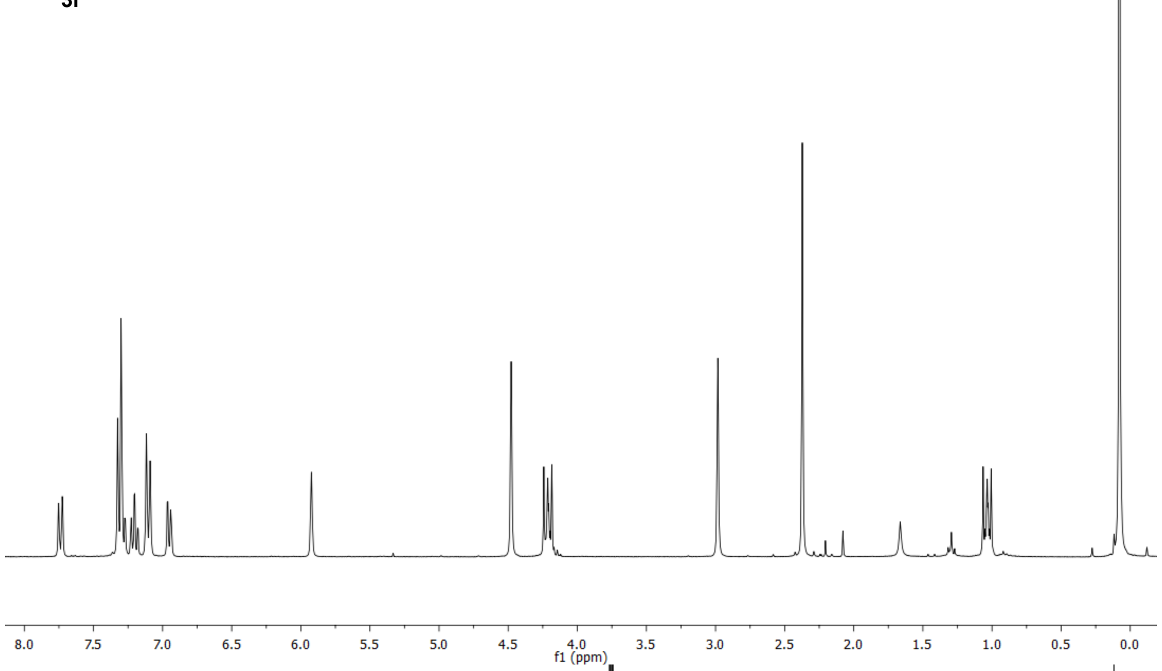
8.0 7.5 7.0 6.5 6.0 5.5 5.0 4.5 4.0 3.5 3.0 2.5 2.0 1.5 1.0 0.5 0.0
f1 (ppm)

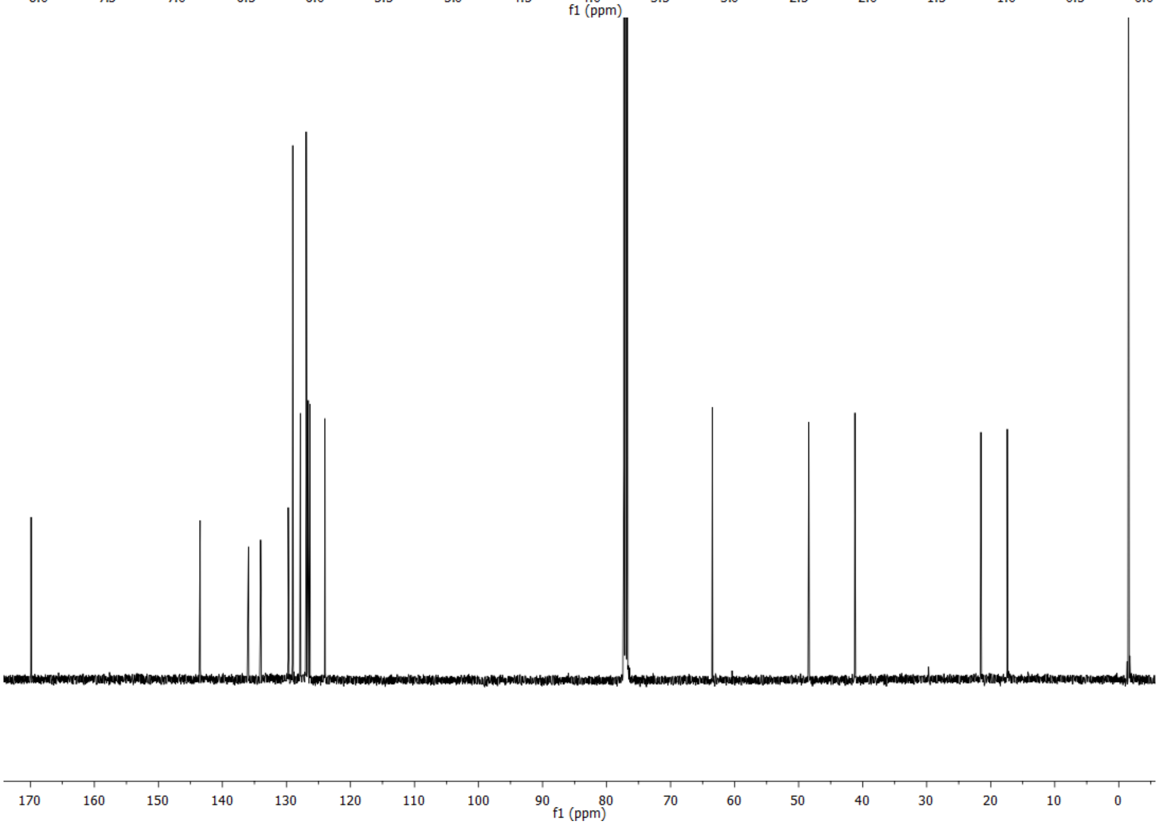
170 160 150 140 130 120 110 100 90 80 70 60 50 40 30 20 10 0
f1 (ppm)

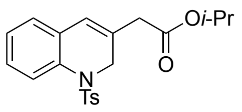
Oi-Pr
O
N
Ts

3m

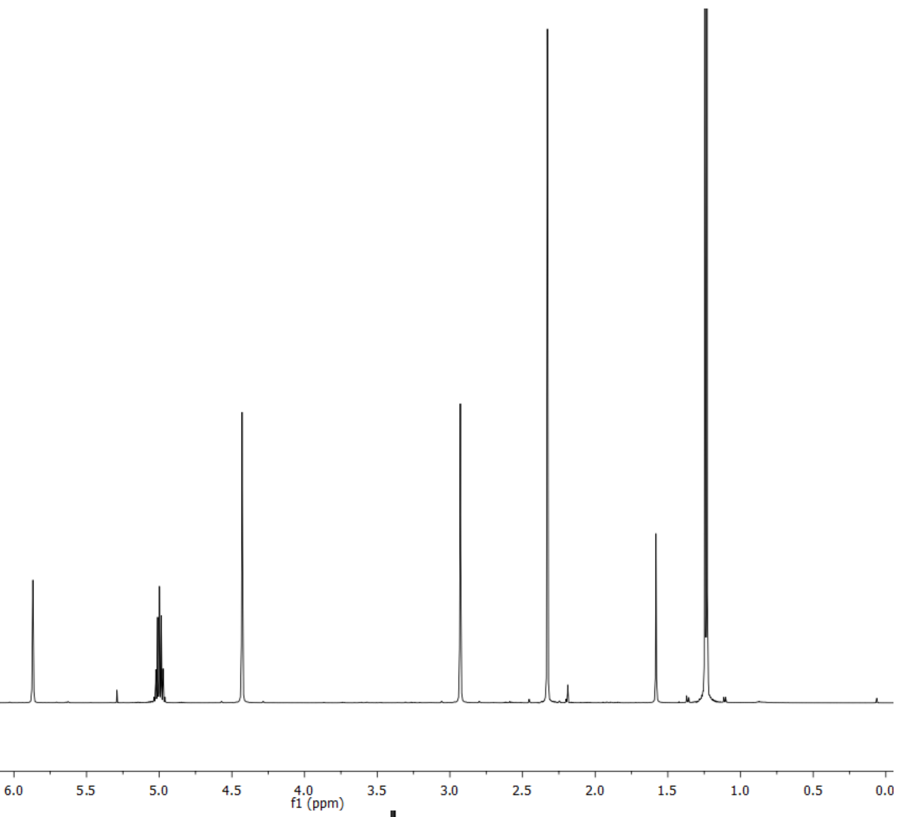
7.5 7.0 6.5 6.0 5.5 5.0 4.5 4.0 3.5 3.0 2.5 2.0 1.5 1.0 0.5 0.0
f1 (ppm)

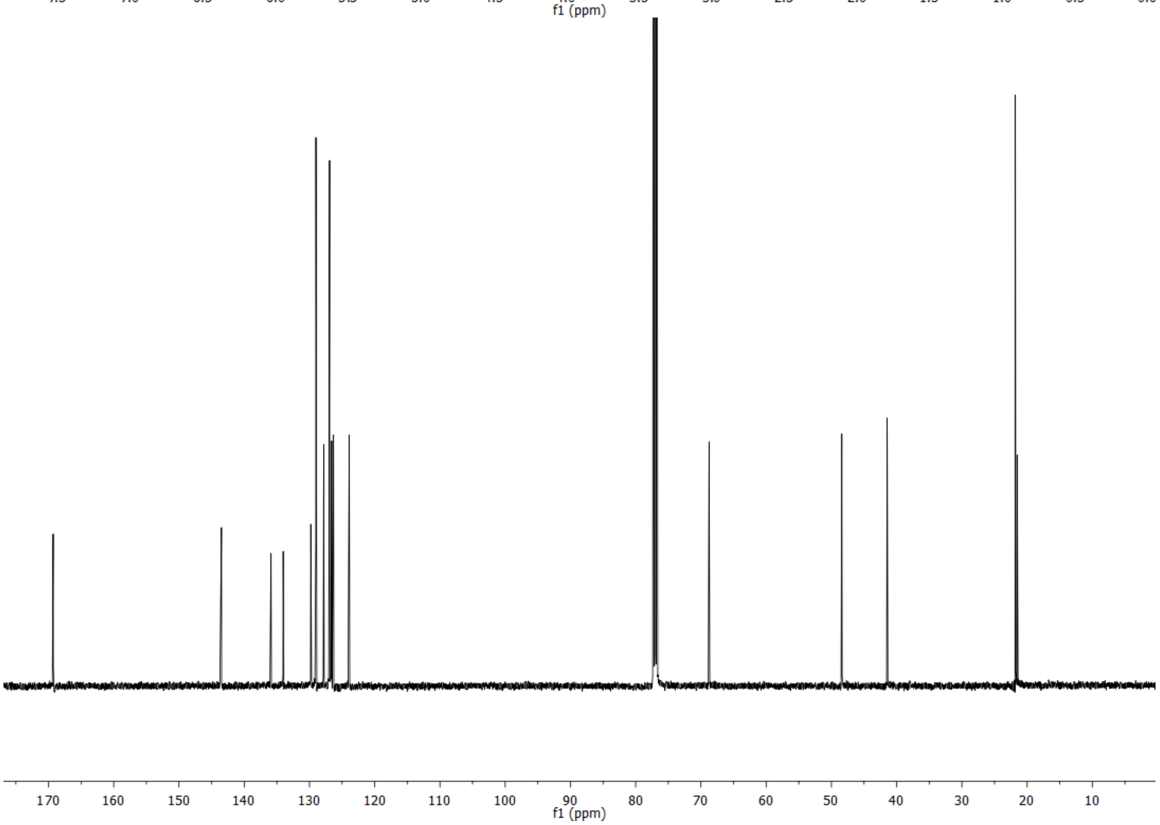
170 160 150 140 130 120 110 100 90 80 70 60 50 40 30 20 10
f1 (ppm)

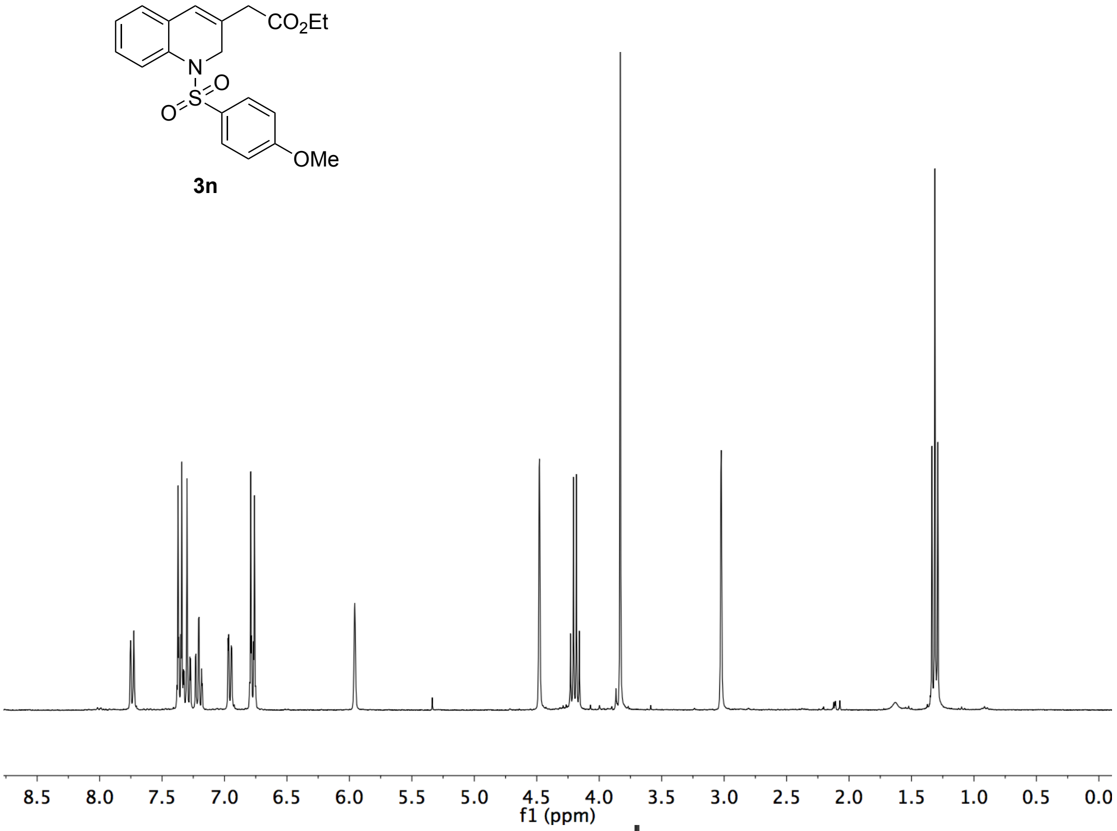
CO2Et
N
S
O
O
OMe
3n
8.5 8.0 7.5 7.0 6.5 6.0 5.5 5.0 4.5 4.0 3.5 3.0 2.5 2.0 1.5 1.0 0.5 0.0
f1 (ppm)

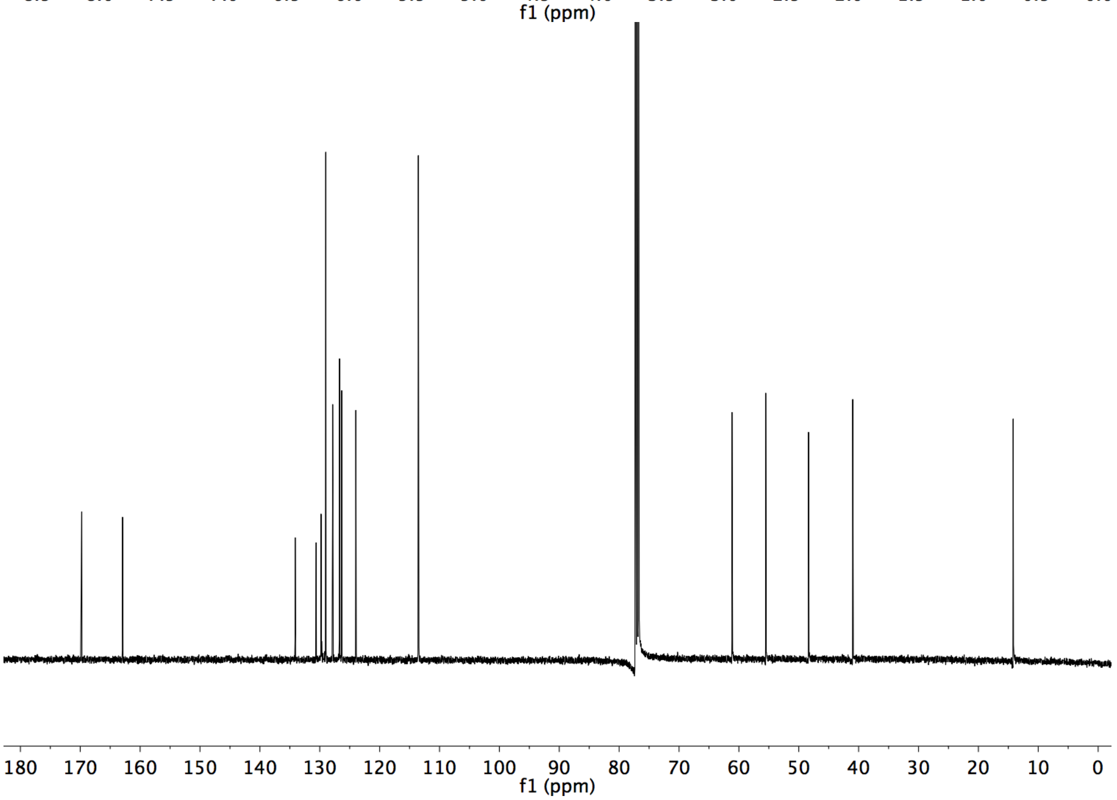
180 170 160 150 140 130 120 110 100 90 80 70 60 50 40 30 20 10 0
f1 (ppm)

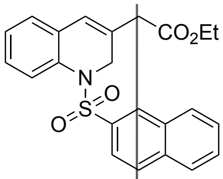
CO2Et
N
S
O
O

3o

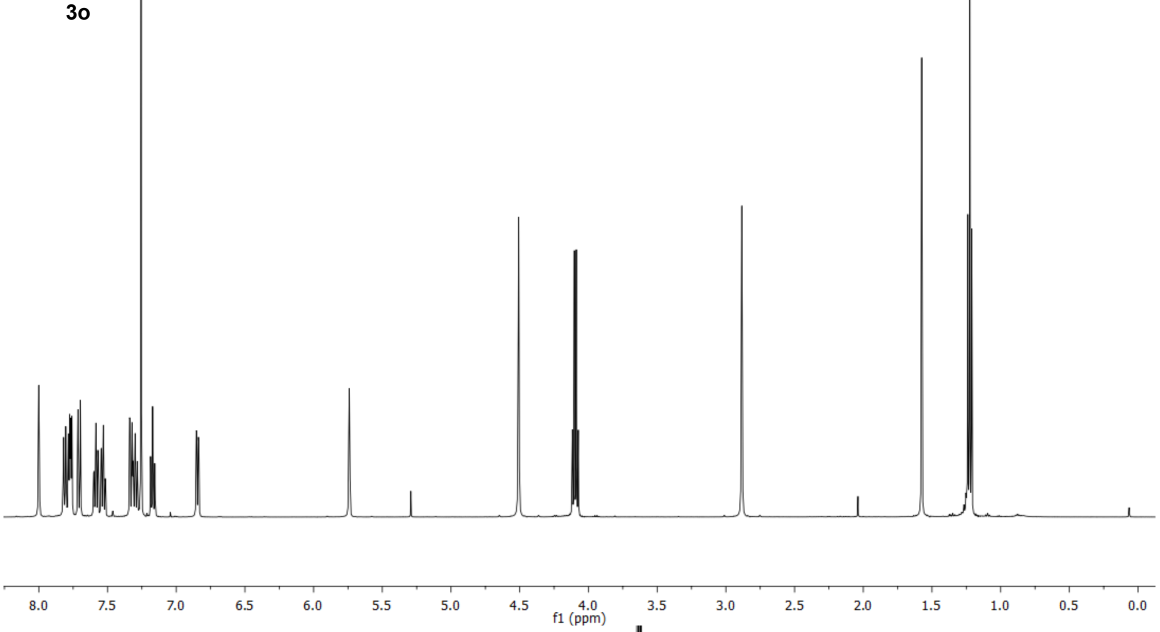
8.0 7.5 7.0 6.5 6.0 5.5 5.0 4.5 4.0 3.5 3.0 2.5 2.0 1.5 1.0 0.5 0.0
f1 (ppm)

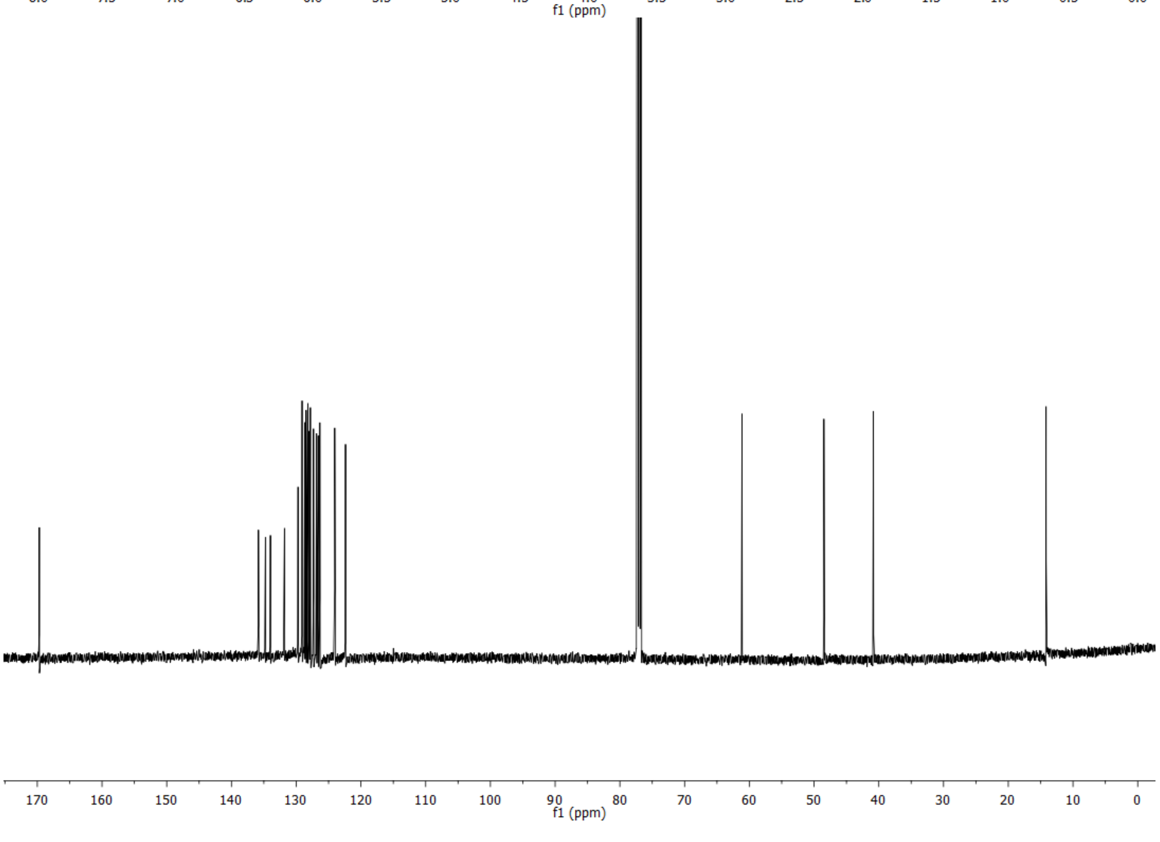
170 160 150 140 130 120 110 100 90 80 70 60 50 40 30 20 10 0
f1 (ppm)

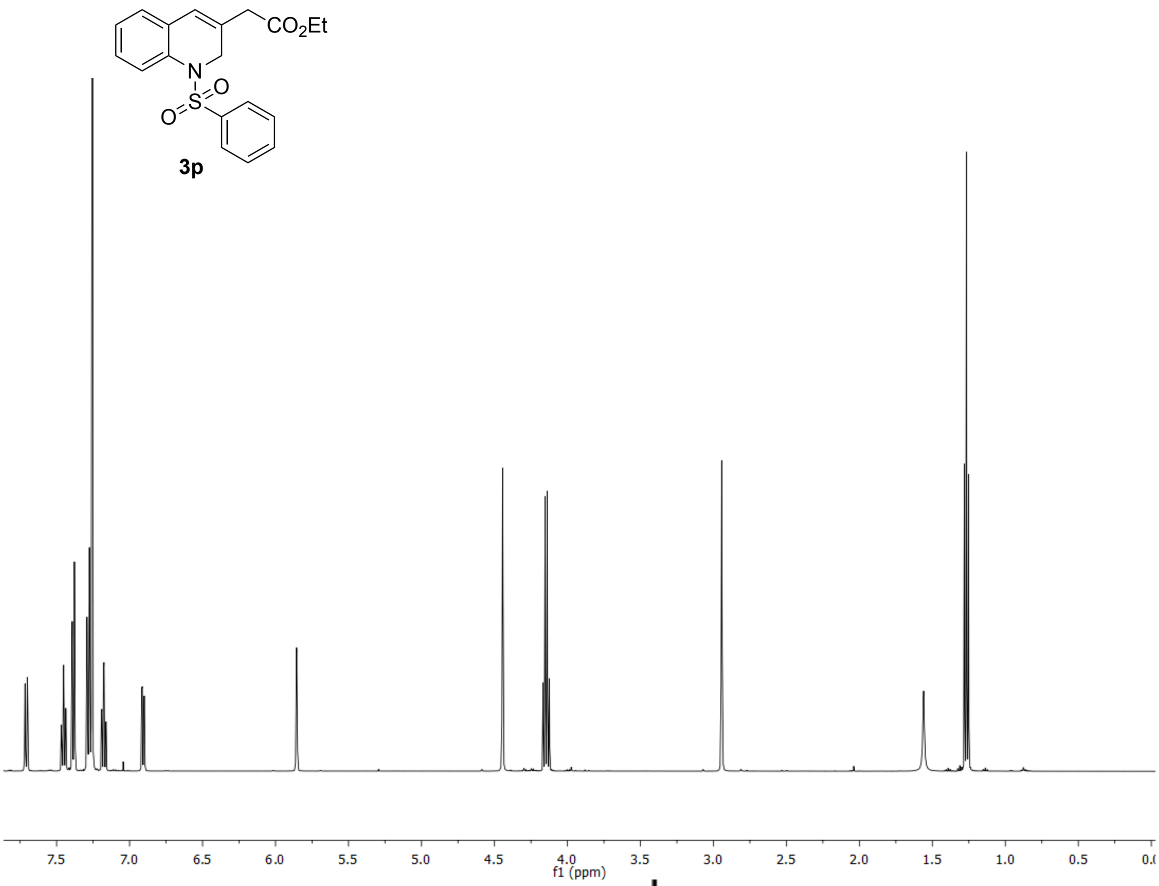
CO2Et
N
O
S
O
3p
7.5
7.0
6.5
6.0
5.5
5.0
4.5
4.0
3.5
3.0
2.5
2.0
1.5
1.0
0.5
f1 (ppm)

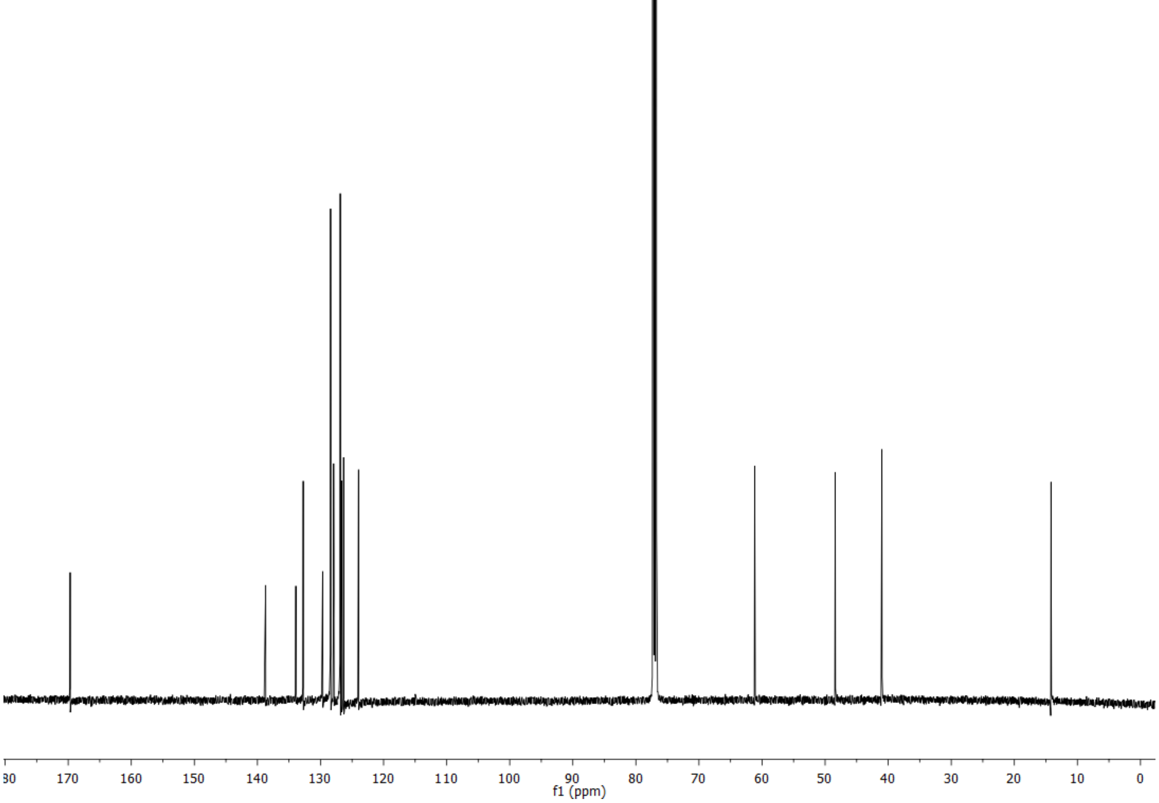
170
160
150
140
130
120
110
100
90
80
70
60
50
40
30
20
10
0
f1 (ppm)

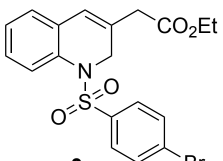

3q

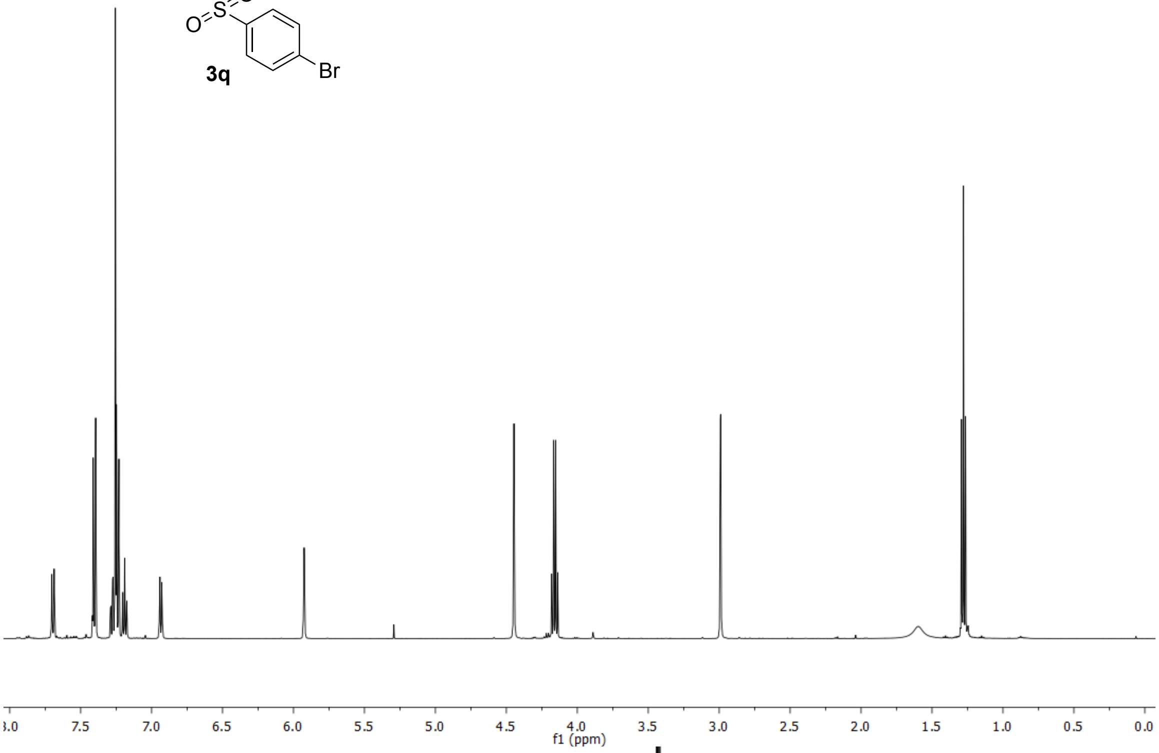

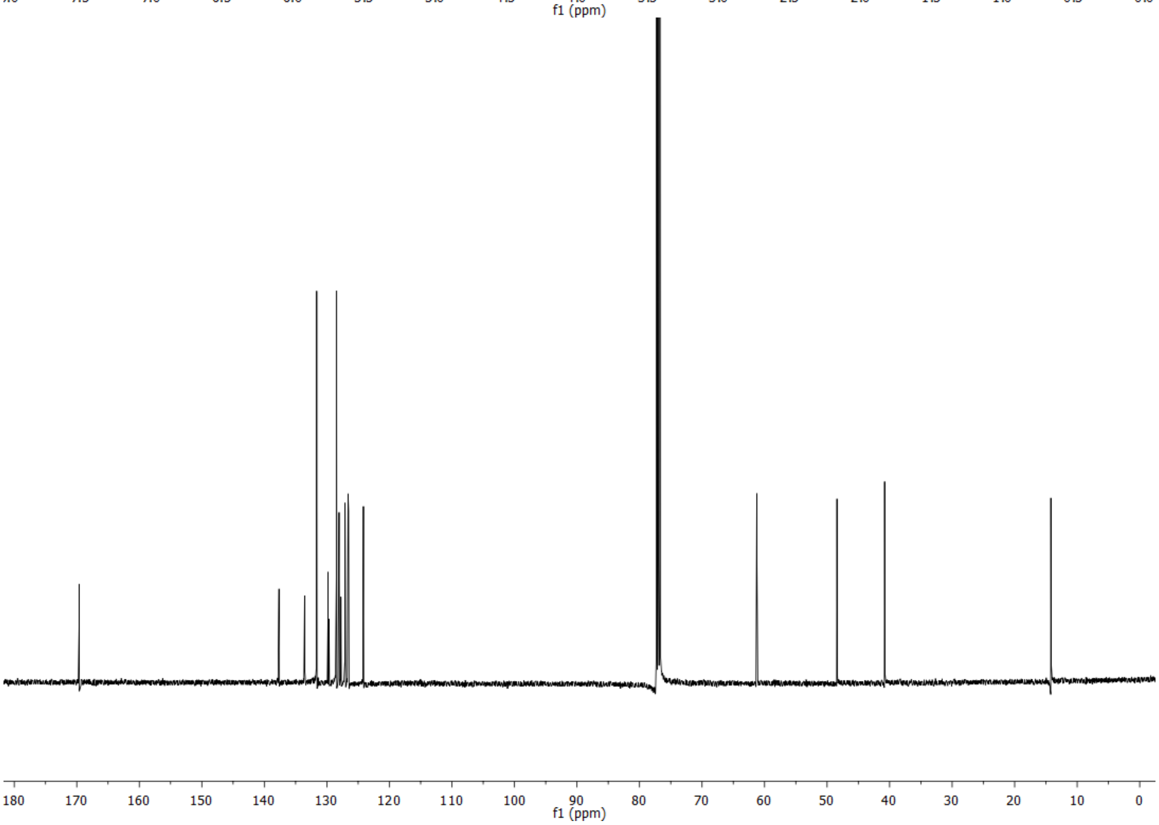

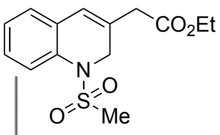
CO2Et
N
S
O
O
Me

3r

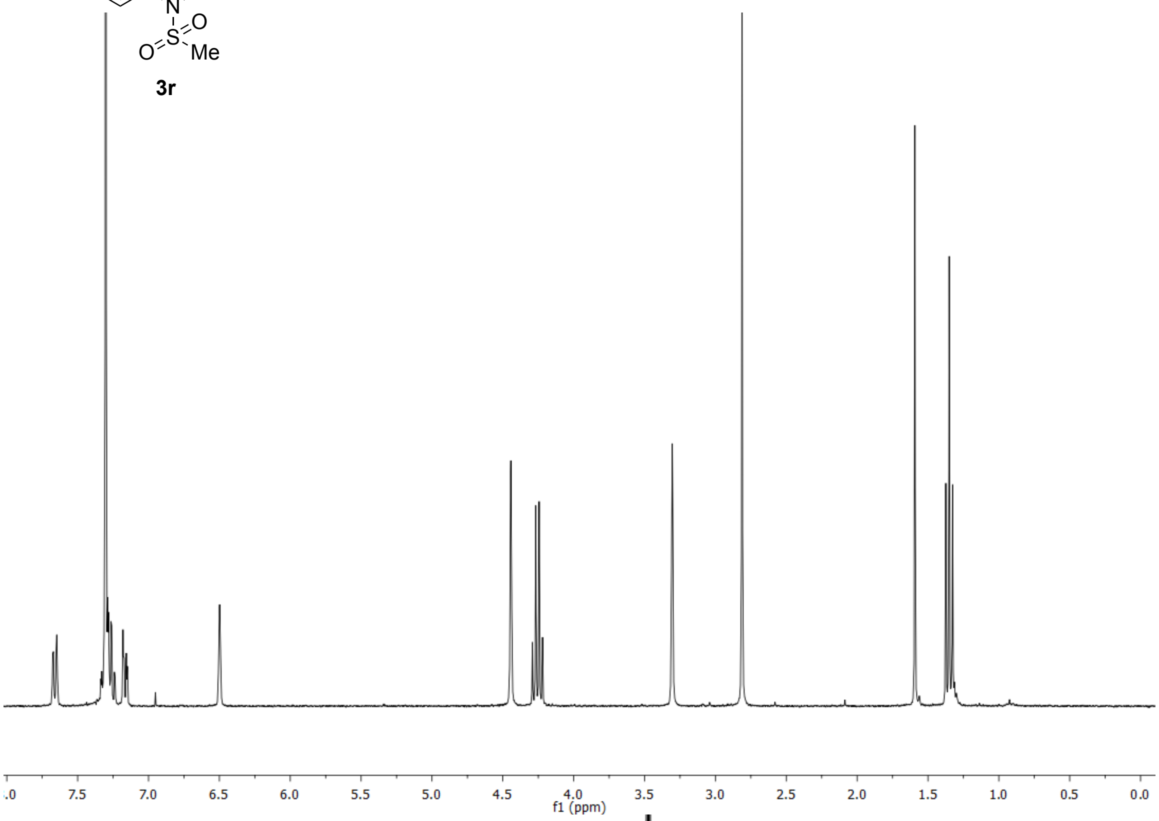
7.5
7.0
6.5
6.0
5.5
5.0
4.5
4.0
3.5
3.0
2.5
2.0
1.5
1.0
0.5
0.0
f1 (ppm)

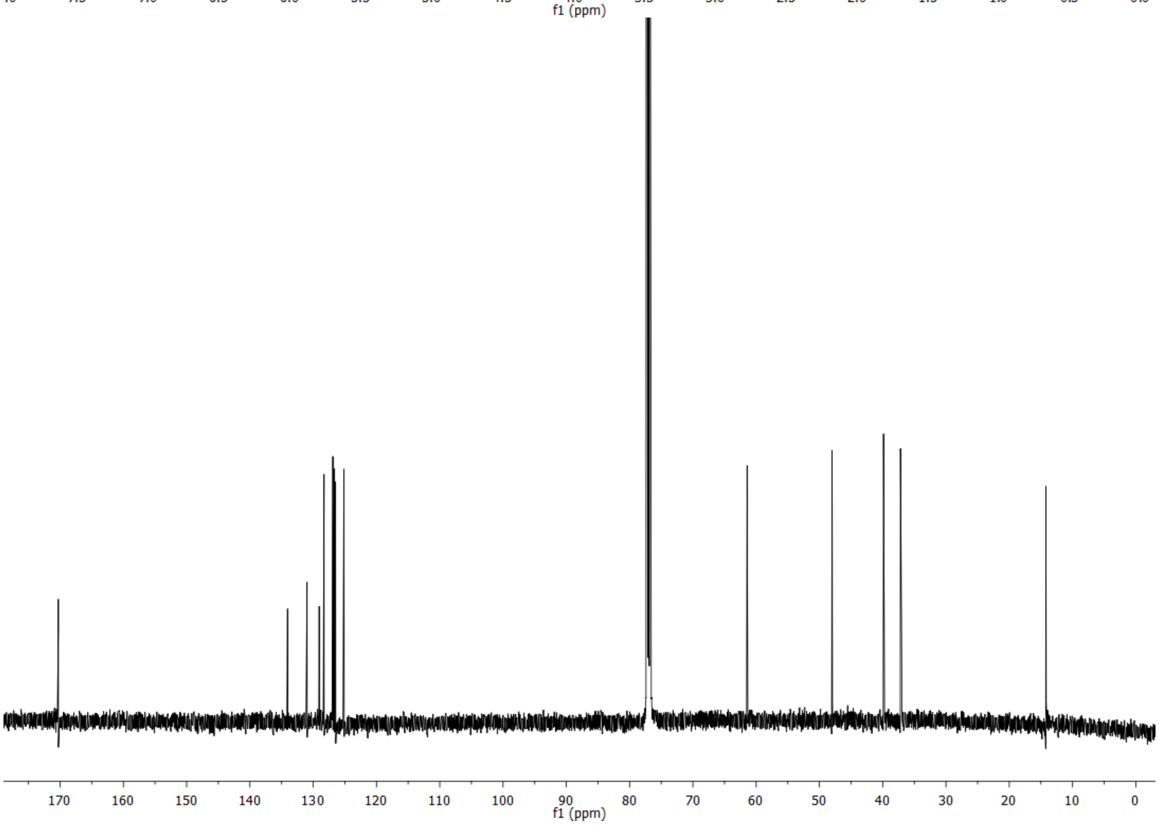
170
160
150
140
130
120
110
100
90
80
70
60
50
40
30
20
10
0
f1 (ppm)

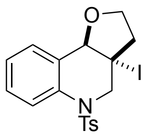
O
I
N
Ts

9

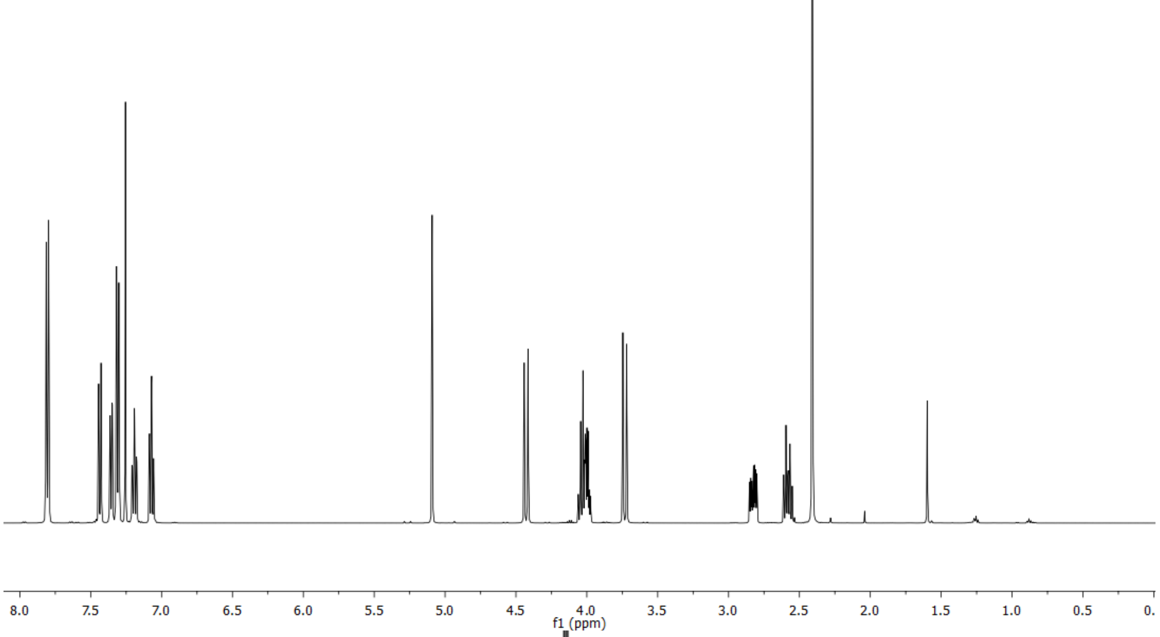
8.0 7.5 7.0 6.5 6.0 5.5 5.0 4.5 4.0 3.5 3.0 2.5 2.0 1.5 1.0 0.5 0.
f1 (ppm)

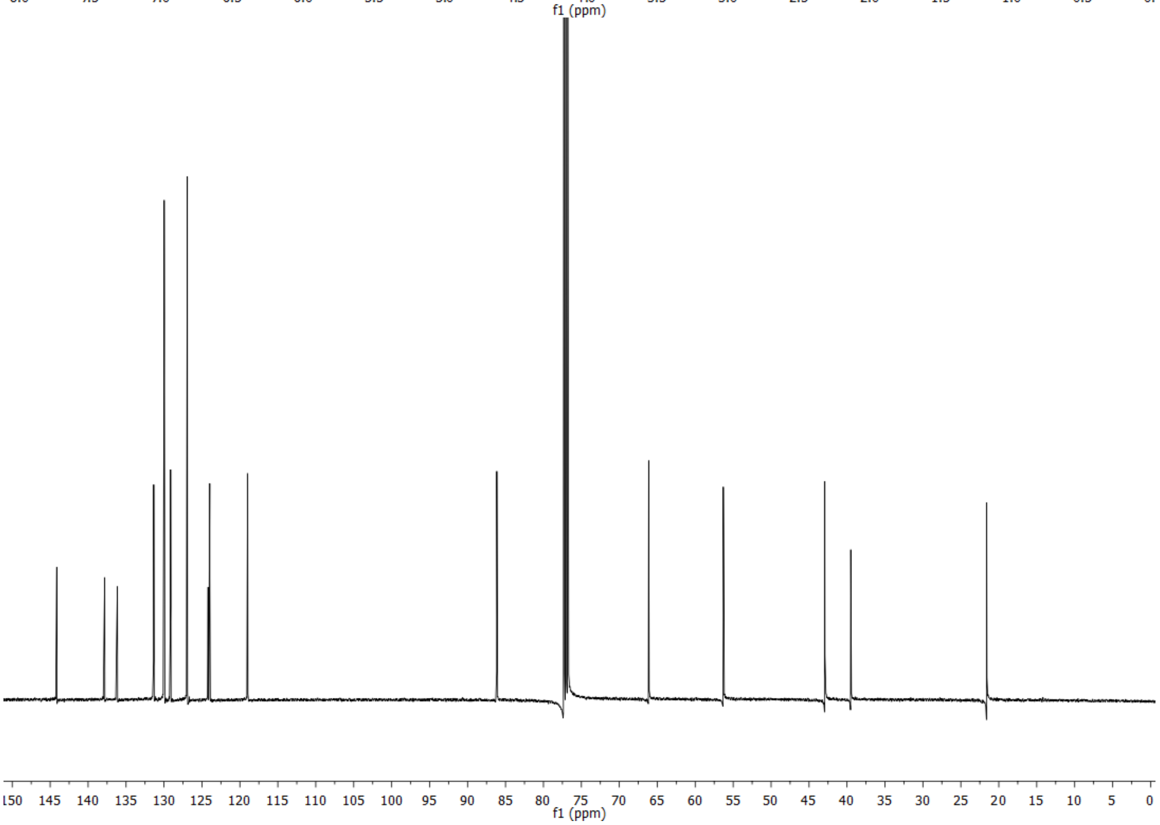
150 145 140 135 130 125 120 115 110 105 100 95 90 85 80 75 70 65 60 55 50 45 40 35 30 25 20 15 10 5 0
f1 (ppm)

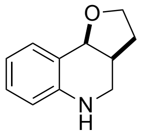

11

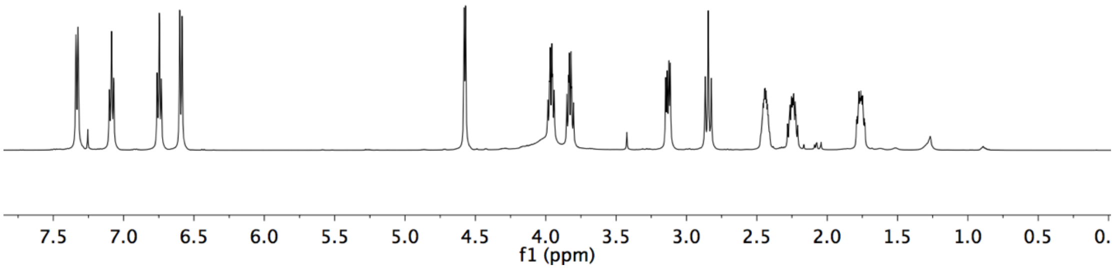
7.5
7.0
6.5
6.0
5.5
5.0
4.5
4.0
3.5
3.0
2.5
2.0
1.5
1.0
0.5
0.
f1 (ppm)

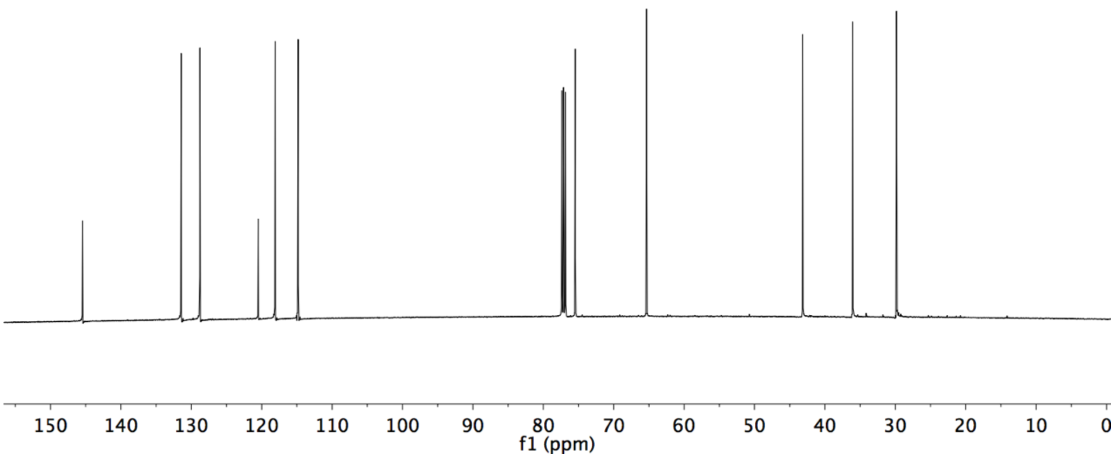
150
140
130
120
110
100
90
80
70
60
50
40
30
20
10
0
f1 (ppm)

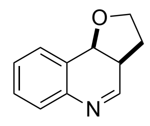
O
N

12

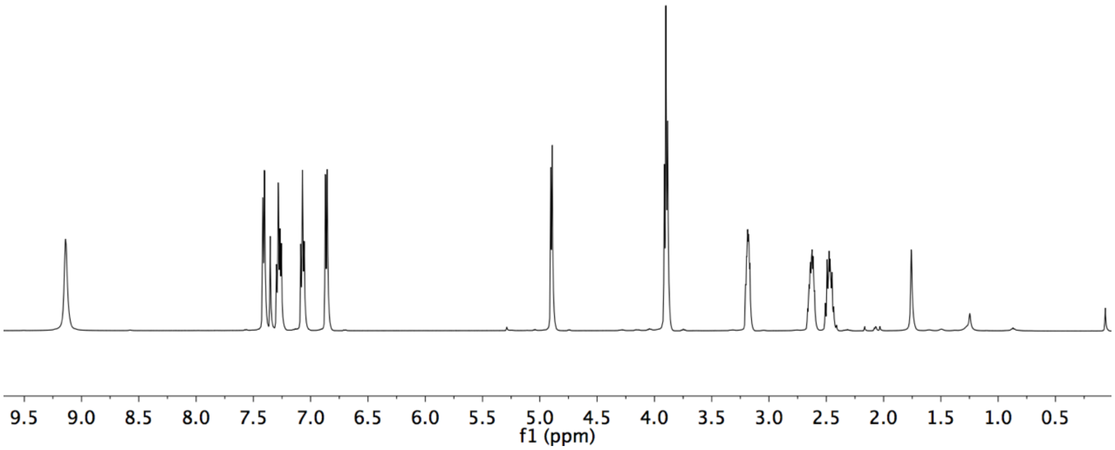
9.5 9.0 8.5 8.0 7.5 7.0 6.5 6.0 5.5 5.0 4.5 4.0 3.5 3.0 2.5 2.0 1.5 1.0 0.5
f1 (ppm)

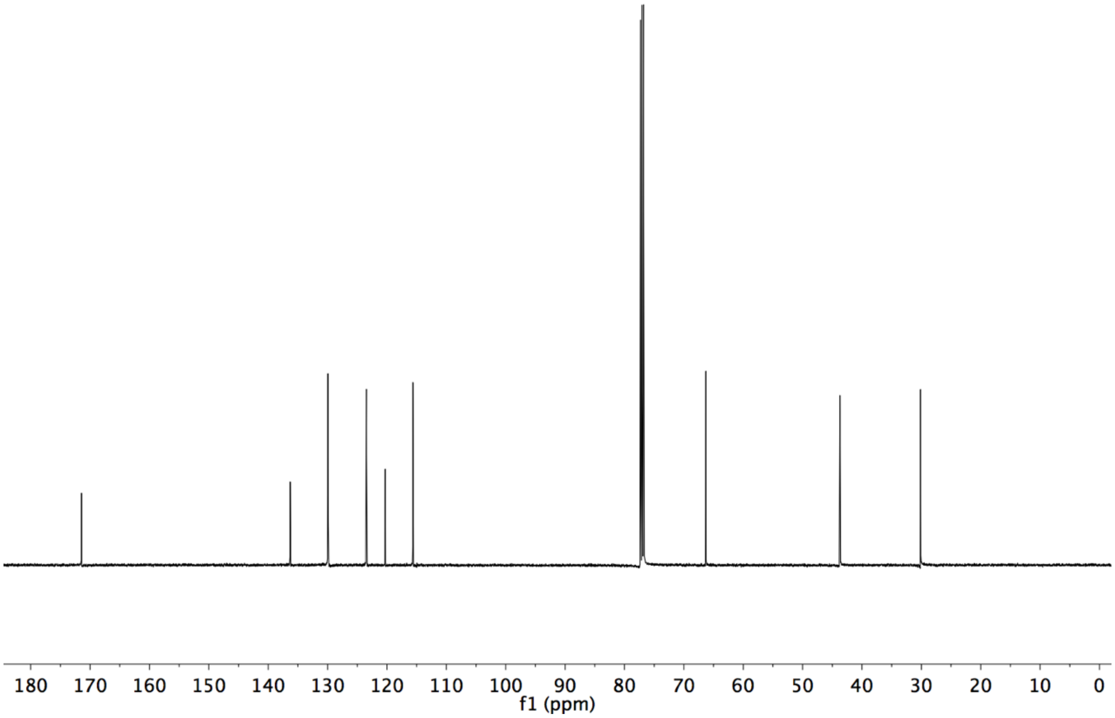
180 170 160 150 140 130 120 110 100 90 80 70 60 50 40 30 20 10 0
f1 (ppm)

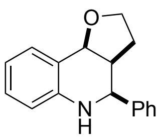

13-*cis*

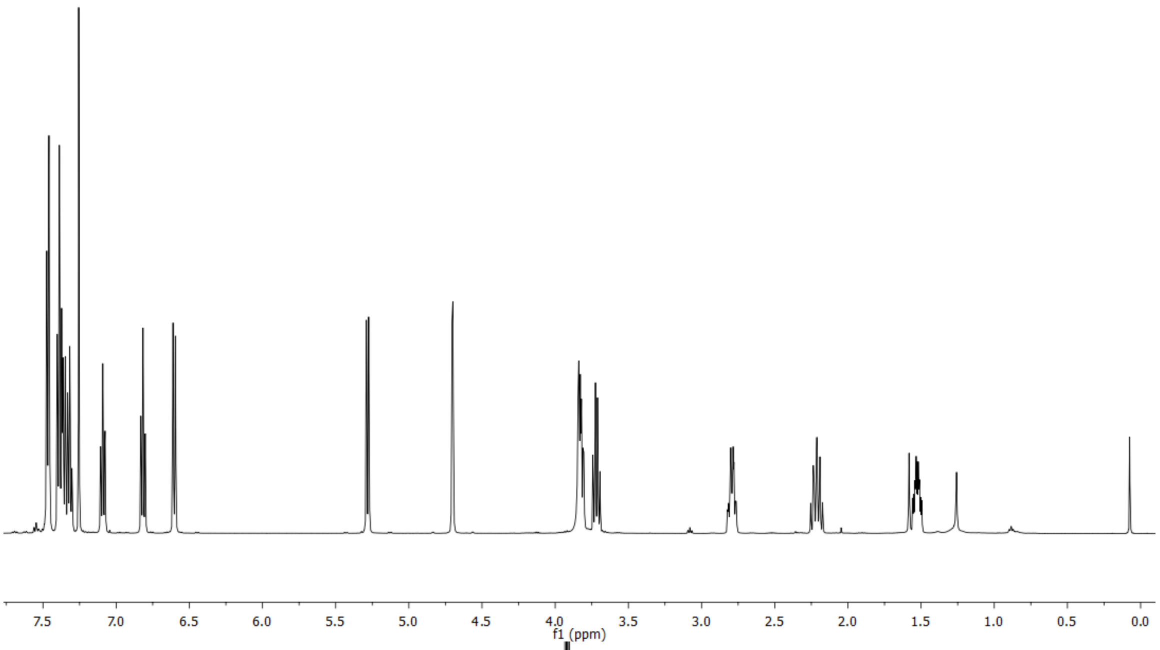

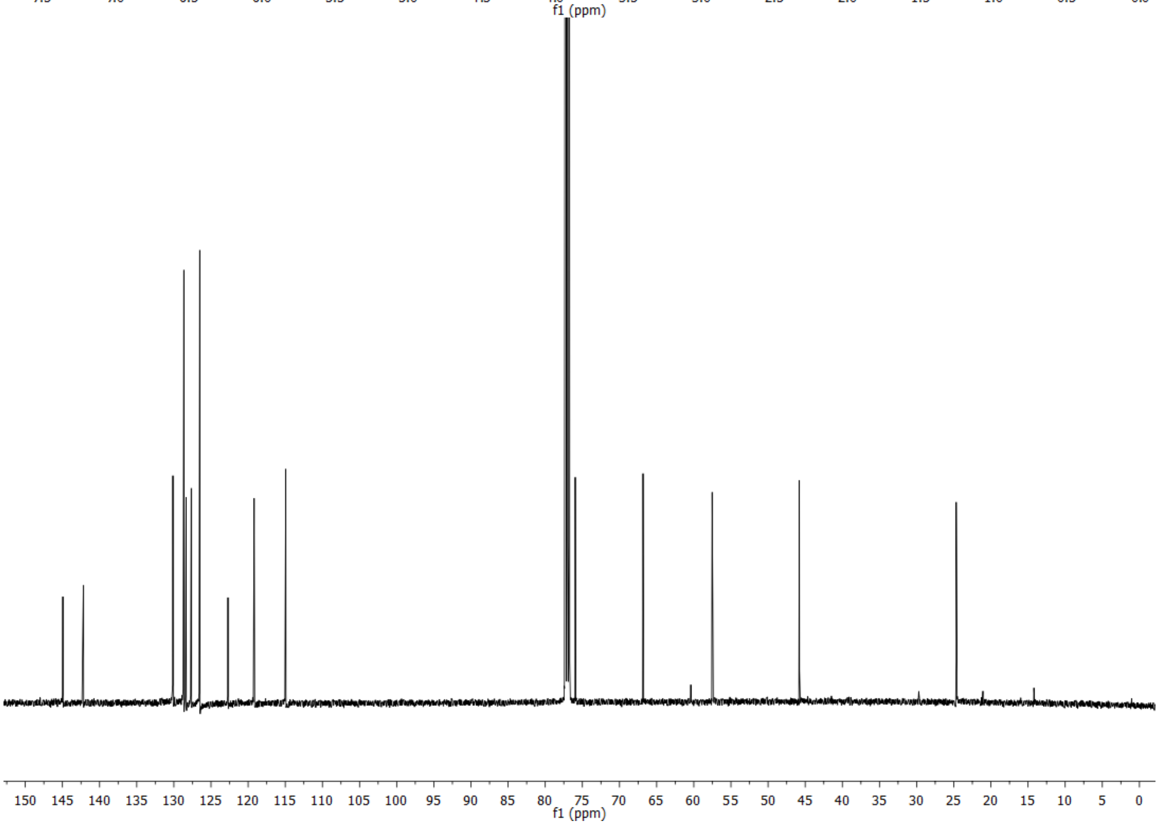

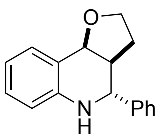

13-*trans*

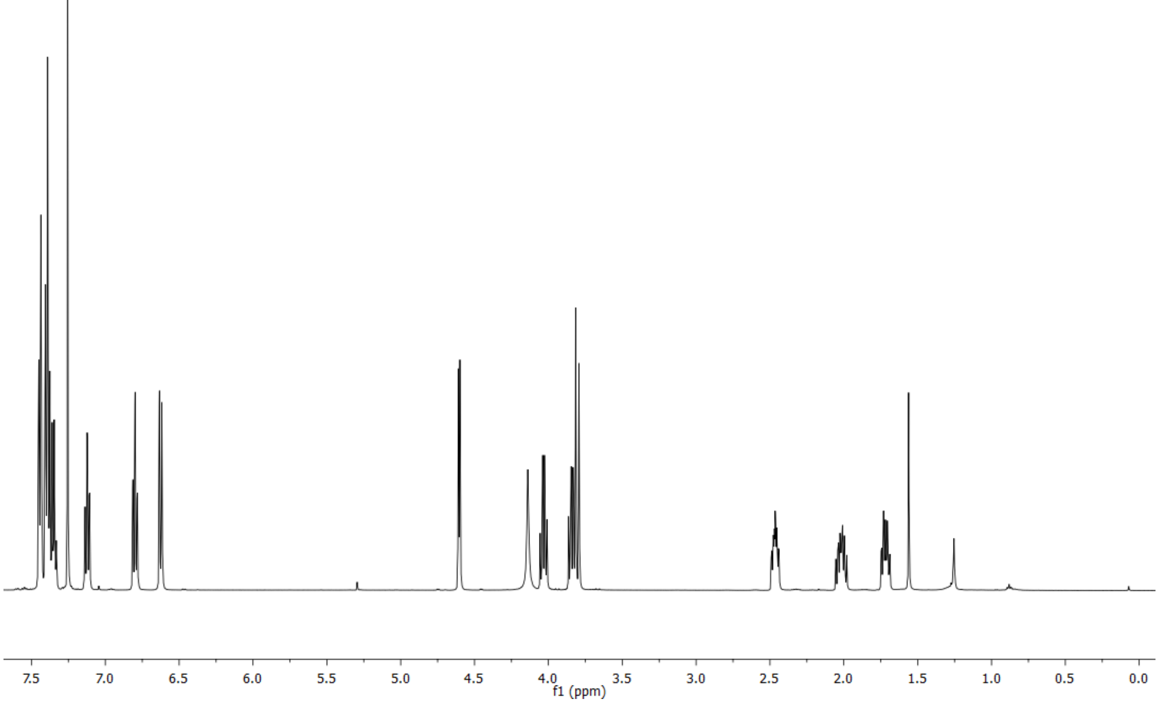

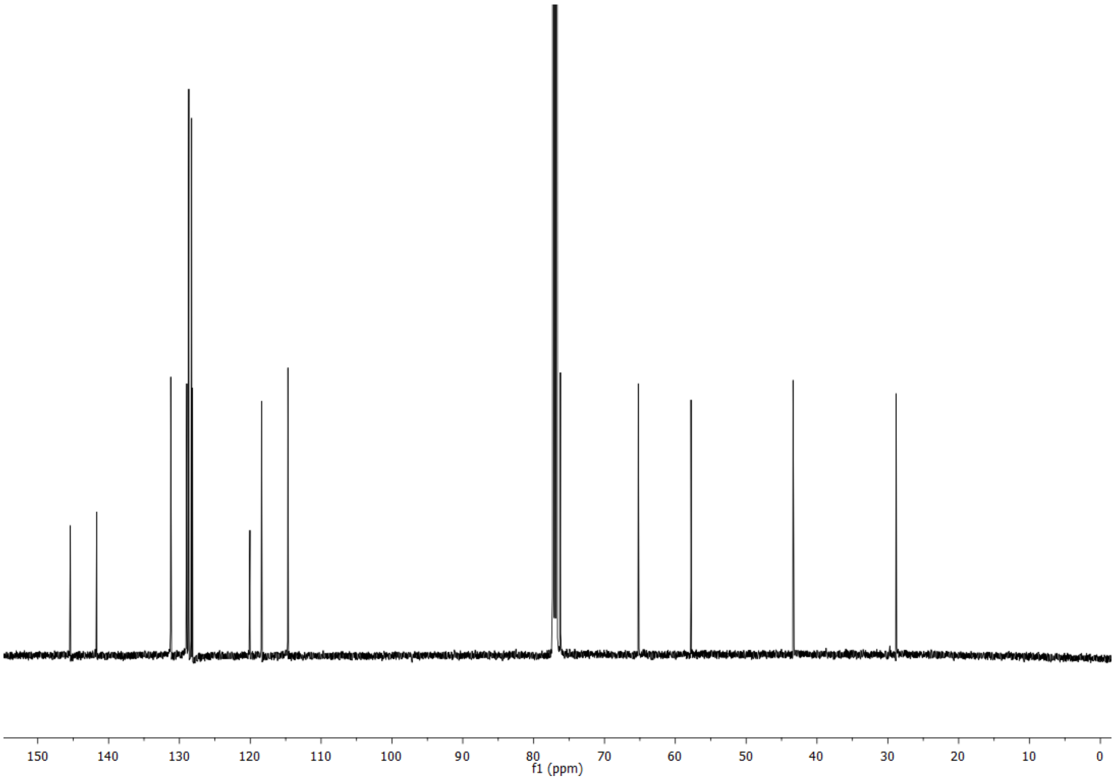

Supplement: Supplementary file 1 [file SC-009-C7SC04381C-s001.pdf]
